# Protein Structure Inspired Discovery of a Novel Inducer of Anoikis in Human Melanoma

**DOI:** 10.3390/cancers16183177

**Published:** 2024-09-17

**Authors:** Fangfang Qiao, Thomas Andrew Binkowski, Irene Broughan, Weining Chen, Amarnath Natarajan, Gary E. Schiltz, Karl A. Scheidt, Wayne F. Anderson, Raymond Bergan

**Affiliations:** 1 Eppley Institute for Research in Cancer and Allied Diseases, University of Nebraska Medical Center, Omaha, NE 68105, USA; 2 Department of Computer Science, University of Chicago, Chicago, IL 60637, USA; 3 Department of Medicine, Northwestern University, Chicago, IL 60611, USA; 4 Department of Chemistry, Northwestern University, Evanston, IL 60208, USA; 5 Department of Biochemistry and Molecular Genetics, Northwestern University, Chicago, IL 60611, USA

**Keywords:** protein structure, drug discovery, anoikis, computational biology

## Abstract

**Simple Summary:**

Drugs work by binding to a specific 3D structure on a protein. Drug discovery has historically been driven by prior knowledge of function, either of a protein or chemical. This knowledge of function then drives investigations to probe chemical/protein interactions. We undertook a different approach. We first identified unique 3D structures, agnostic of function, and investigated whether they could lead us to innovative therapeutics. Using a synchrotron-based X-ray source, we first determined high-resolution structures of hundreds of proteins. With a supercomputer running analytical programs created by us, we identified novel 3D structures and screened for chemicals binding them. We then tested their ability to inhibit cancer growth without damaging normal cells. We identified a potent inhibitor of a deadly cancer, melanoma. It was not toxic to normal cells even at 2100-fold higher doses. It worked by inducing anoikis, a fundamental process of known importance for cancer. Therapeutics that selectively induce anoikis are needed. In summary, we demonstrate the power of using a 3D protein structure as the starting point to discover new biology and drugs.

**Abstract:**

Drug discovery historically starts with an established function, either that of compounds or proteins. This can hamper discovery of novel therapeutics. As structure determines function, we hypothesized that unique 3D protein structures constitute primary data that can inform novel discovery. Using a computationally intensive physics-based analytical platform operating at supercomputing speeds, we probed a high-resolution protein X-ray crystallographic library developed by us. For each of the eight identified novel 3D structures, we analyzed binding of sixty million compounds. Top-ranking compounds were acquired and screened for efficacy against breast, prostate, colon, or lung cancer, and for toxicity on normal human bone marrow stem cells, both using eight-day colony formation assays. Effective and non-toxic compounds segregated to two pockets. One compound, Dxr2-017, exhibited selective anti-melanoma activity in the NCI-60 cell line screen. In eight-day assays, Dxr2-017 had an IC50 of 12 nM against melanoma cells, while concentrations over 2100-fold higher had minimal stem cell toxicity. Dxr2-017 induced anoikis, a unique form of programmed cell death in need of targeted therapeutics. Our findings demonstrate proof-of-concept that protein structures represent high-value primary data to support the discovery of novel acting therapeutics. This approach is widely applicable.

## 1. Introduction

It is established that protein structures determine function. The formation of specific three-dimensional (3D) structures serves to mediate specific functions. However, the analysis of 3D structures has inherent limitations. They relate to its complexity and cost, especially in the context of how one 3D structure may interact with a different 3D structure. The latter situation is typical of when one is considering how a protein may interact with drug [1,2,3,4]. For this reason, studies of structure are typically undertaken only after there is some evidence supporting the potential functional relevance [5].

Prior evidence of function underlies structure-based drug design approaches [5]. Its central focus is understanding the structure for the purpose of designing a drug that can interact with it. In reality, however, the initial steps in this process are based on prior evidence that the target structure is considered to be biologically important [6]. Such evidence may stem from the primary protein sequence and its association with function, the function of the whole protein, or a host of other sources of information indicative of function. However, efforts that involve first considering the 3D structure as a type of primary source of information are not well represented. We undertook a series of investigations to examine the hypothesis that 3D structures represent an important source of primary information, and that unique structures represent unique functions that, in turn, can inform downstream applications.

The process of drug discovery is well suited to such an application. This is because therapeutic agents mediate their effects by binding to specific sites on proteins. Such sites, by definition, are functionally relevant. The binding of a given therapeutic agent alters that function in a desirable manner. Target identification in drug discovery constitutes a central focus, and there are two general approaches: targeted drug discovery (TDD) and phenotype-based drug discovery (PDD). While TDD begins the drug discovery process with knowledge of the protein target, ideally its 3D structure, it is driven by prior evidence supporting the role of the target in disease [7]. An important limitation with TDD relates to the complexity of disease and that a pre-identified single target may fail to address that complexity. A PDD approach addresses this limitation, but is associated with the highly difficult task of deconvolution of complex biology and ultimate target identification [8]. Additional reasons for focusing on drug discovery relate to its overall importance, its longstanding inherent limitations, and the need for new approaches better tailored to identifying novel acting drugs. Longstanding limitations relate to failure rates of over 96%, spiraling costs, with current average estimates around USD 2 billion, and a development timeframe of over 10 years [6,9,10,11,12].

To address the hypothesis that structure may serve as an important entry point into the process of drug discovery, we conducted a series of proof-of-concept investigations. We first created an experimentally determined, high-resolution protein crystal structure library. Using an integrated suite of analytics, designed by us to probe dynamic protein structures and scripted to run on a supercomputer, we then screened the library for unique structures. We thereby identified 3D protein structures whose function was not otherwise characterized and whose structural properties were compatible with the potential to bind drug-like compounds. Using this analytic suite, we accessed a library of 60 million compounds and conducted an in silico screen for compounds predicted to bind the identified 3D structures. We then acquired a panel of compounds and evaluated them in a panel of cell-based experimental screens for efficacy and toxicity. One compound, Dxr2-017, inhibited human melanoma cell growth at low nanomolar concentrations, but also exhibited little toxicity on human bone marrow stem cells even at concentrations over 2100-fold higher. Of high interest, Dxr2-017 was shown to induce anoikis, a type of programmed cell death that occurs upon cell detachment. Cell detachment is necessary for cancer cells to form metastasis. To survive after detachment, cancer cells must be resistant to anoikis. Therefore, anoikis and its regulation are fundamental to cancer biology, and effective therapeutics that induce it are needed [13,14,15,16]. These investigations provide proof-of-concept that protein structures represent a powerful untapped source of primary information that can be used across several applications, inclusive of discovering novel acting drugs.

## 2. Materials and Methods

### 2.1. Chemicals and Cell Lines

Compounds were purchased from manufacturers (Figure S1). The following human cells were obtained from American Type Culture Collection (ATCC): breast cancer, MCF-7 and MDA-MB-231; lung cancer, H226 and A549; colon cancer, HT116 and HT29; prostate cancer, LNCaP; melanoma, M14 and SK-MEL-5. The origin and characteristics of human prostate cancer PC3-M cells were previously described by us [17]. Cells were cultured as previously described by us [18,19]. Briefly, cells were drawn from frozen stocks. Stocks were created by expanding original cells for 2 passages and creating up to 20 frozen stock vials. Cell stocks were maintained in the vapor phase of liquid nitrogen, and once thawed they were not used for more than 20 passages. When a given cell line stock was depleted to 3 vials, the expansion process was repeated. When the stock passage number approached 20, new cells were acquired from ATCC, except for PC3-M cells. PC3-M cells were obtained from the originator of this cell line, and associated stocks were below 30 passages. Cells were cultured in RPMI 1640 media for MCF-7, H226, LNCaP, PC3-M, and M14, in DMEM for MDA-MB-231, in Ham’s F-12K for A549, in McCoy’s 5a for HT29 and HT116, and in EMEM for SK-MEL-5, with the addition of 10% heat-inactivated fetal bovine serum and 1% penicillin–streptomycin. For all cells except M14 and SK-MEL-5, 1% L-glutamine was added to the purchased media. All cells were maintained at 37 °C in 5% CO_2_ under sub-confluent exponential growth conditions and passaged 1–2 times weekly. All cells were manipulated one cell line at a time, with complete sterilization of working surfaces in between. Testing for Mycoplasma (PlasmoTest™, InvivoGen, San Diego, CA, USA) was performed bi-monthly.

All cell lines were authenticated. All were acquired through ATCC or the originator, as above. After acquisition, cells were grown and expanded under quarantine conditions (i.e., maintained in a dedicated incubator). They were then expanded, stored as primary stocks, and not used until confirmed mycoplasma-negative. Further, morphologic examination under light microscopy was conducted and was required to match the phenotype of that published by the originator. In the case of estrogen- and androgen-responsive MCF-7 and LNCaP cells, respectively, studies confirmed their growth responsiveness to the respective hormones. This was performed under hormone-free culture conditions (i.e., use of charcoal-stripped fetal bovine serum and phenol-red-free growth media), as previously described by us [19,20]. Specifically, for LNCaP cells, androgen-mediated induction of the androgen-responsive gene, prostate-specific antigen (PSA), was confirmed by qRT-PCR, using R1881 as the androgen [19]. For MCF-7 cells, estradiol was used to confirm estrogen-mediated induction of the estrogen-responsive genes, progesterone receptor (PGR), trefoil factor 1 (TFF1), and cathepsin D (CTSD) [20].

### 2.2. Development and Characterization of a Protein X-ray Crystallographic Library

Using the Argonne National Laboratory Advanced Proton Source, we generated and characterized a high-resolution X-ray crystallographic protein library, as described by us [21,22,23]. In brief, protein constructs with His tags were over-expressed in *E. coli*, followed by purification by affinity and size exclusion chromatography. Sitting drop crystallization screens were then performed, with temperature and solution conditions being particular to each protein. Crystals were cryopreserved in liquid nitrogen, and diffraction data were collected. The latter were conducted through the Life Sciences Collaborative Access Team or the Structural Biology Center at the Advance Photon Source, Argonne, Illinois. Protein structures were deposited by the Center for Structural Genomics of Infectious Diseases (CSGID). All structures were evaluated by the Protein Data Bank (PDB) crystal structure quality metrics. CSGID guidelines for deposition included a resolution of at least 2.9 Å and completeness of at least 90%, meeting parameters for the crystallographic R-factor (which varies as a function of resolution) and the number of waters defined. Further, the model should have >95% of residues in favored Ramachandran regions (with structural rationale required for outliers). Also, the structure should not contain any residues that violate the reported geometry, and the root mean square deviation from ideal values should approximate 0.015 for bond lengths and 1.5 degrees for angles. The rationale for target protein selection, early examples of target protein structures, and an overview of the high-throughput structure determination methods have been published [21,23]. The CSGID target proteins whose structures were deposited in the PDB, the PDB codes for access to those structures, and those that were utilized in the computational screens are provided at: https://csgid.org/screenings. All final structures were deposited in the PDB [24].

### 2.3. Development and Validation of a Physics-Based Protein Structure Analysis Platform

We developed a physics-based protein structure analysis platform, allowing consideration of dynamic protein flexibility and the influence of solvent, that was scaled to run on a supercomputer, thus providing the capability to probe large protein libraries for novel structural motifs. Specifically, we integrated a suite of methodologies, i.e., Computed Atlas of Surface of Proteins (CASTp), pocket and void Surfaces Of Amino acid Residues (pvSOAR), SurfaceAlign, and SurfaceScreen, scaled to operate on the 163,840 core Intrepid BlueGene/P (Intrepid BG/P) supercomputer of the Argonne National Laboratory Advanced Leadership Computing Facility (ALCF), and we organized them into a computational pipeline designed to identify, characterize, and compare protein surfaces [25,26,27,28,29,30,31].

### 2.4. In Silico Compound Screening

We constructed a computational pipeline for docking and scoring compound interactions with protein pockets [29,30,31,32,33,34,35]. It created a lead generation tool that is unbiased to *a priori* functional knowledge annotations. This agnostic approach aims to discover conserved three-dimensional shapes and atomic patterns that may provide the underlying foundation for protein–ligand interactions. This information can then be exploited for novel investigations.

Protein surfaces were identified using the CASTp methodology. It identifies solvent-accessible voids and concavities through an analysis of three-dimensional protein structure coordinate files [31]. This approach is strictly based on geometry and does not utilize any biochemical information to determine pockets or voids. As such, there are many surfaces identified on a target protein that will not be susceptible to ligand binding and need to be removed from consideration. To ascertain which surfaces may be candidates for small-molecule interactions, a series of screening techniques was applied such that the shape, volume, and physicochemical texture of each surface were leveraged in a manner of increasing scrutiny (and computational complexity).

**Figure 1:**
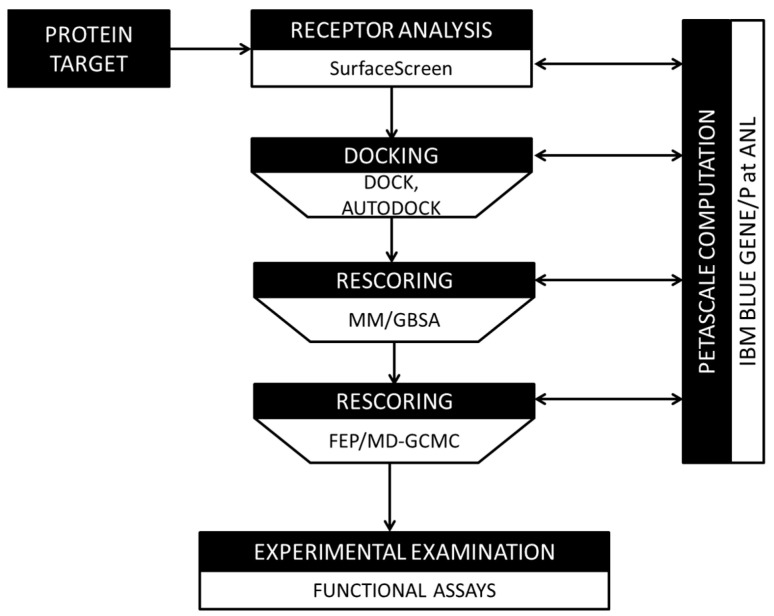
Computational pipeline schema for evaluating compound binding.

Each identified protein surface was first modeled as a probability distribution, referred to as a ShapeSignature, representing a set of interatomic distances between atoms [36]. Similar ShapeSignatures have been shown to share geometric shape properties and can be efficiently compared using a Kolmogorov–Smirnov test [37]. All target protein surfaces were compared against the Global Protein Surface Survey (GPSS), an annotated library of protein surfaces that have been curated from the Protein Databank (PDB). Those surfaces that share similarities to known binding pockets were identified as candidates to propagate through the pipeline (Figure 1).

Further, recognizing that biochemical function relies on the combination of shape and chemical compatibility, the latter, in the context of shape, was considered with SurfaceAlign. SurfaceAlign compares coordinate combination sets of two surfaces, formed by the different chemical properties of its constituent amino acid groups. It does so through decomposition of single-value coordinates to identify the least square rotational matric and translation vector. Taking into consideration these parameters it performs a superimposition of the structures that minimizes the root mean square distance (RMSD) between them [38]. This approach considers both the reported three-dimensional coordinates of each amino acid’s atoms, the coordinate RMSD (cRMSD), and a flexible orientation vector coordinate structure, the orientation RMSD (oRMSD), that accounts for flexibility in the pocket [28]. Statistical Monte Carlo simulation techniques were applied, evaluating randomly generated surface alignments, to assess the significance of these scores. This metric ensures that similar atom types are in the same orientation between the surfaces, eliminating surfaces that may have similar geometry but lack key atoms for ligand binding. Finally, the global surface volume overlap Tanimoto (gSVOT) score was calculated between the superimposed surfaces to evaluate the volume that their shapes occupy. This presumes that surfaces with similar volumes should provide space to bind similar molecules. In this manner, the geometry, atomic composition and orientation, and volumes are composited in the generation of a composite SurfaceScreen Score, representing the similarity of surfaces and their potential to bind similar ligands [28,39,40,41,42].

These candidate surfaces, representing novel target sites, were used as receptor input into a docking pipeline that incorporates the AutoDock [32] and DOCK [33] docking applications. Distinct docking software packages were used in tandem to provide broad coverage of protein–small molecule docking strategies. This included accounting for protein residue, compound flexibility, and scoring schemes. At this stage, the speed and throughput of molecules were prioritized. Top-ranking docking poses from each method were then ‘funneled’ into increasingly sophisticated and computationally complex approximations of free energy of interaction using the molecular-mechanics-generalized Born surface area (MM-GBSA) method [34]. It provides a more accurate, albeit computationally expensive, estimation of binding affinities. All subsequent rescoring efforts utilized the top-scoring poses, as obtained from the docking results. The raw docking scores were treated as relative to the dataset for a given protein target, and only the ranked positions were used to determine the threshold for propagation. The top hits were finally rescored using the molecular dynamics free-energy perturbation grand canonical Monte Carlo (FEP/MD-GCMC) method [35]. The result of the screening pipeline presented a list of lead compounds that were filtered, scored, and sorted using increasingly complex methods in an effort to balance accuracy and efficiency.

### 2.5. Growth Assays

Initial colony formation screening assays were conducted on all acquired compounds and examined at five different compound concentrations. Those meeting efficacy criteria underwent a second-level screen examining nine different compound concentrations. Eight-day colony formation assays of cancer cells were performed as described by us [43]. Briefly, 0.5–1.0 × 10^3^ cells were seeded into each well of a 12-well plate. Twenty-four hours later, the compound was added at the following final concentrations: 0 (DMSO-only control), 0.017, 0.12, 0.82, 5.7, and 40 µM. For each concentration, there were N = 2 replicates, i.e., 2 wells. Eight days later, plates were stained with trypan blue, visualized under light microscopy, and groups of three or more cells were scored as colonies. The mean number of colonies was then determined and expressed as the percentage of controls. Second-level screens were performed on compounds inhibiting colony formation by greater than 50% at 40 µM in at least two cell lines. Second-level assays used the following final concentrations of compound: 0 (DMSO only), 0.0061, 0.018, 0.05, 0.16, 0.49, 1.48, 4.44, 13.33, and 40 µM. All compounds were stored under desiccated conditions in a sealed container at minus 20 °C upon arrival. One day prior to use, they were dissolved in DMSO, stored at minus 20 °C as stock solutions, thawed just prior to use, and not reused.

In addition to screens, eight-day colony formation assays were conducted for certain experiments, as denoted. When measuring the effect of 5-fluorouracil (5FU) on HT29 colon cancer cells, the following final 5FU concentrations were examined: 0 (DMSO only), 0.0012, 0.012, 0.12, 0.82, 5.7, 20, and 40 µM. When measuring the effect of Dxr2-017 on melanoma cell growth, the following concentrations were examined: 0 (DMSO only), 0.001, 0.01, 0.1, 1.0, and 10.0 µM. Melanoma studies were performed on M14 and SK-MEL-5 human melanoma cell lines. For each Dxr2-017 concentration for each cell line, there were N = 3 replicates, and experiments were performed a total of 3 separate times (each with N = 3 replicates). The IC50 values were calculated using nonlinear regression analysis. Data from individual experiments were log-transformed and fitted to a dose–response curve using the four-parameter logistic model of GraphPad Prism 10 software. The IC50 values were determined using the dose–response inhibition function. IC50 values from each of three different experiments were used to calculate mean ± SEM.

### 2.6. Bone Marrow Stem Cell Colony Formation Assays

Human hematopoietic stem cell trilineage colony formation assays were performed as previously described by us [20]. Briefly, human cord blood CD34+ cells (catalog #: 70008.5) were purchased from StemCell Technology Inc. and seeded at 1 × 10^4^ cells per 35 mm dish. Cells were cultured in MethoCult™ Express (StemCell Technologies, Inc. (Vancouver, BC, Canada)) semisolid media, per the manufacturer’s instructions, in the presence of compound or vehicle (for controls). Compounds were added at final concentrations matching those used in the initial growth assay screens. At eight days, colonies (i.e., groups of ≥10 cells) were counted. At fourteen days, individual colonies of erythroid burst-forming units (BFU-E), colony-forming unit granulocyte–macrophage (CFU-GM), and colony-forming unit granulocyte, erythrocyte, monocyte, and megakaryocyte (CFU-GEMM) were identified based on the manufacturers’ morphologic characterization criteria. Assays were run in replicates of N = 2, and some were run in replicates of N = 4, as denoted. The mean number of colonies was then determined and expressed as the percentage of controls. The effect of each compound on inhibiting the growth of cancer cells relative to its toxic effects on normal bone marrow was evaluated. This was carried out by comparing colonies at 8 days for each cancer cell line to that of bone marrow. This information was used to calculate the efficacy ratio, as follows: efficacy ratio = (percent of remaining cancer cell colonies)/(percent of remaining bone marrow colonies). This was performed for all compounds at 40 µM and for selected compounds at 5.7 µM, as denoted. For compounds nominated for second-level screens, bone marrow colony formation assays were also repeated. Compounds were added at final concentrations matching those used in the initial screens.

### 2.7. Cell Cycle Analysis

A total of 1 × 10^4^ M14 cells and 1 × 10^4^ SK-MEL-5 cells were seeded into 10 cm culture dishes. Twenty-four hours later, cells were treated in replicates of N = 3, with Dxr2-017 at 20 nM for M14 and 50 nM for SK-MEL-5, or vehicle for controls for a total of eight days, with replacement of media, along with Dxr2-017 or vehicle, at day five. Cells were then collected, filtered to obtain single-cell suspensions, equal numbers of cells were fixed in 1 mL of 70% ethanol at 4 °C for 1 h, washed with PBS, and resuspended in 1 mL of Telford reagent (1 mM EDTA disodium salt, 2.5 U/mL RNAse A, 75 µM Propidium Iodide, and 0.1% Triton X-100 in PBS), and then incubated at 4 °C overnight. Cells were analyzed by flow cytometry (FACSCalibur analyzer, BD Biosciences). Cells were gated on forward scatter (FSC) versus side scatter (SCC) to remove debris, followed by gating of single cells using PI (FL2)-Area versus PI (FL2)-Width. Cell cycle analysis was performed on the PI (FL2)-Area parameter, and the percentages of cells within G0/G1, S, and G2/M phases were determined using Modfit LT 4.0.5.

### 2.8. Western Blot and Image Acquisition

After treatment of M14 and SK-MEL-5 cells with different concentrations of Dxr2-017 or vehicle for 3 or 8 days, as denoted, cell images were captured at 20X in a transmitted light channel using the EVOS™ M5000 Imaging System (Invitrogen (Waltham, MA, USA)). Western blots were performed as previously described by us [19]. Briefly, cells were collected and lysed using RIPA lysis buffer (25 mM Tris-HCl pH 7.6, 150 mM NaCl, 1% NP-40, 1% sodium deoxycholate, and 0.1% SDS; Thermo Scientific^TM^ (Waltham, MA, USA), #89900), supplemented with protease and phosphatase inhibitor cocktail (Thermo Scientific^TM^, #78442) and 1 mM of PMSF. Resultant Western blots were probed using antibodies against cleaved caspase 3 (Cell Signaling Technology (Danvers, MA, USA), #9664), pan-cadherin (Cell Signaling Technology, #4068), and β-actin (Santa Cruz (Santa Cruz, CA, USA), #sc-47778).

## 3. Results

### 3.1. Identification of Unique Structural Motifs of High Potential Pharmacologic Value

We are seeking to address the fundamental hypothesis that the protein structure in-and-of-itself constitutes powerful information that can serve as a starting point from which to initiate the process of discovering novel therapeutics. This requires access to well-annotated protein structure information and an ability to analyze it in an in-depth and meaningful manner.

Using the Argonne National Laboratory Advanced Proton Source Synchrotron (APS), our group includes members of consortia that have constructed and maintained several beam lines. This allowed the acquisition of high-resolution protein structure information. Through the Midwest Center for Structural Genomics, the Protein Structure Initiative, and the Center for Structural Genomics of Infectious Diseases (CSGID) initiative, funded, respectively, by the National Institute of General Medical Sciences (NIGMS) and the National Institute for Allergy and Infectious diseases (NIAID), our group used the APS as a platform to define a library of 3D protein structures [22]. Protein structures were determined using state-of-the-art equipment and facilities, were well annotated, and were made accessible to the public through the Protein Data Bank (PDB).

For the proof-of-concept goals of the current study, we considered protein structures deposited in the PDB through the CSGID initiative. The CSGID targets proteins that are from a priority list of bacterial pathogens. As one of many possible sampling approaches, we considered the first group of 220 deposited structures (Figure S2).

**Figure 2:**
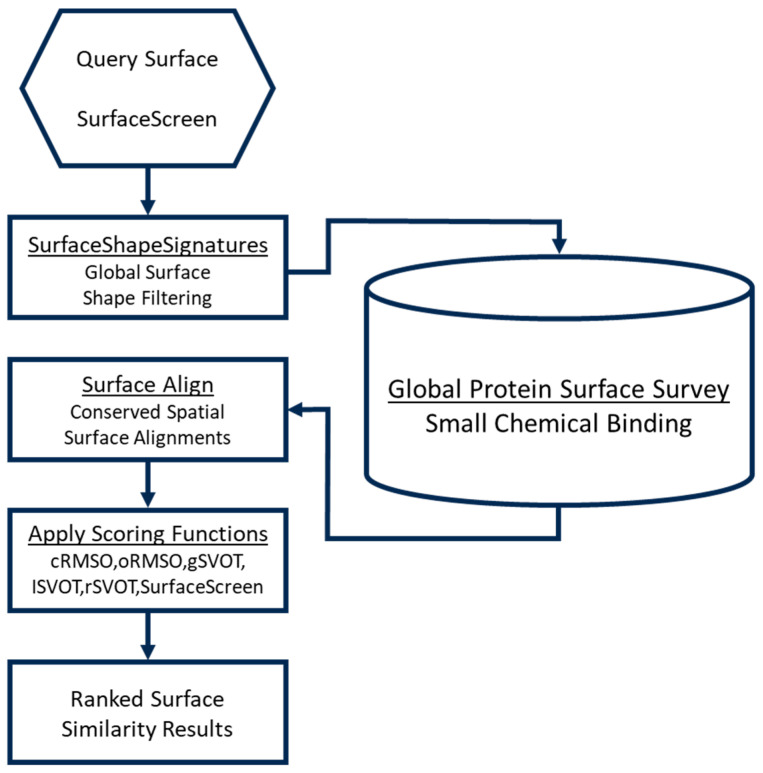
Schema for identification and characterization of 3D protein structures with the potential to bind therapeutically active small molecules.

From the proteins in Figure S2, we used the SurfaceScreen methodology to identify and consider potential binding sites for small molecules [26,29,30,31]. We have previously demonstrated that functional surfaces are the most highly conserved regions of a protein, that they exhibit strong ligand specificity, and that ligand binding preferences can be assessed in proteins lacking sequence and/or structural similarity [25,26,27,28,44]. SurfaceScreen allows for surface comparisons by decomposing them into global shape and local physicochemical texture (Figure 2). There are several subcomponents of SurfaceScreen. It uses ShapeSignature to characterize the global shape of a CASTp-identified [31] solvent-accessible surface as a probability distribution [36], and performs comparisons between the probability distributions of two surfaces, conducted using Kolmogorov–Smirnov test methods [37]. Further, recognizing that biochemical function relies on the combination of shape and chemical compatibility, the latter, in the context of shape, is considered with SurfaceAlign. SurfaceAlign compares coordinate combination sets of two surfaces, formed by the different chemical properties of its constituent amino acid groups, through decomposition of single-value coordinates to identify the least square rotational matric, translation vector, and RMSD [38]. This approach considers both the reported three-dimensional coordinates and a flexible orientation vector coordinate structure that accounts for flexibility in the pocket [28]. Statistical methods incorporate random surface alignments, the global surface volume overlap Tanimoto coefficient, and lead to the generation of a composite SurfaceScreen score [28,39,40,41,42].

We then sought to identify pockets on individual proteins that had the potential to serve as biologically important functional sites and that also had the potential to serve as target sites for small-molecule therapeutics. We first sought pockets of 500 Å^2^, a value based on lower-limit default settings with docking programs in common use [45]. Further, we sought the presence of at least two polar residues within a pocket. A value that was derived by considering that of commercially available drugs, from the Comprehensive Medicinal Chemistry (CMC) database, most side chains were polar, and the modal number of side chains was two [46]. We gave priority to pockets with a single mouth, based on findings that pockets for drug-like ligands are almost always one mouthed [47].

In performing the final selection of sites for further investigation, we considered several additional factors. We prioritized sites whose function was not otherwise characterized. We also prioritized sites exhibiting conservation of structure. During evolution, highly conserved proteins are often required for basic cellular function, stability, and folding kinetics [48,49,50,51,52,53,54,55]. Functional sites, such as enzyme-active sites, are associated with the convergence of spatially conserved amino acid residues. Such conserved functional surfaces exhibit strong specificity and selectivity for compatible ligands [25,26,27,28]. When residues that are conserved across species in a large sequence family come together in space, it indicates an evolutionary bias toward important functionality. This phylogenetic conservation of structure and function supports the hypothesis that important structural elements identified in bacterial proteins have functionally relevant counterparts in human cells. Surfaces with higher degrees of conservation were assigned higher priority, with those conserved into eukaryotes having the highest priority. Another factor we considered was the nature of the protein on which the pocket is located. We assigned higher priority to proteins that, to our knowledge, did not have cognate small-molecule modulators of their function that were advancing into clinical trials.

**Figure 3:**
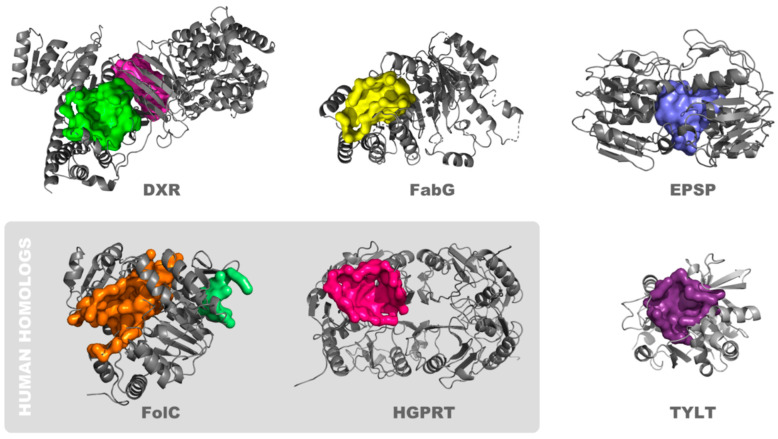
Proteins that contain pockets structurally suited to binding drug-like small molecules. The surfaces of potential binding pockets are depicted, as are ribbon structures of proximal portions of the protein. The proteins are: 1-deoxy-D-xylulose 5-phosphate reductoisomerase (Dxr; Dxr1 (magenta) and Dxr2 (green) binding pockets), ß-ketoacyl acyl carrier protein reductase (FabG), 3-phosphoshikimate 1-carboxyvinyltransferase (EPSP synthase), dihydrofolate synthase (FolC; FolC1 (orange) and FolC2 (light green) binding pockets), hypoxanthine-guanine phosphoribosyltransferase (HGPRT), and glucose-1-phosphate thymidylyltransferase (TYLT).

Taking the above factors into consideration, we selected eight potential binding clefts on six different proteins for further investigation (Figure 3). Two proteins are expressed in bacteria through humans: (1) Hypoxanthine-guanine phosphoribosyltransferase (HGPRT). This enzyme catalyzes the conversion of hypoxanthine or guanine to inosine or guanosine monophosphate and is important in generating purine nucleotides through the purine salvage pathway [56]. Prior reports describe targeting HGPRT with tailored synthesized nucleoside analogs [57], as well as by pentamidine, 1,3-dinitroadamantane, acyclovir, and acyclovir analogs [58,59]. The focus of these reports is on the treatment of parasitic diseases, and higher activity is reported in HGPRT from parasites compared to that from humans. (2) Dihydrofolate synthase (FolC). There are two clefts on this protein, which we designate as FolC1 and FolC2. FolC catalyzes two reactions: its dihydrofolate synthetase activity catalyzes the addition of L-glutamate to dihydropteroate (7,8-dihydropteroate), forming dihydrofolate (7,8-dihydrofolate monoglutamate), and its folylpolyglutamate synthetase activity catalyzes subsequent additions of L-glutamate to tetrahydrofolate, forming folylpolyglutamate derivatives [60]. There are many inhibitors of other enzymes in the folate pathway in widespread clinical use, but those that target FolC are not among them. While FolC is expressed in humans, its dihydrofolate synthetase activity is not present in humans, and efforts to therapeutically target FolC appear to be limited, primarily focused on parasites, especially *P. falciparum* [61].

The other four proteins are primarily expressed in bacteria, and not in humans. (3) 1-deoxy-D-xylulose 5-phosphate reductoisomerase (Dxr), also known as DXP reductoisomerase. There are two pockets on this protein (Dxr1 and Dxr2). Dxr catalyzes the NADP-dependent rearrangement and reduction of 1-deoxy-D-xylulose-5-phosphate (DXP) to 2-C-methyl-D-erythritol 4-phosphate [62]. It is involved in the methylerythritol phosphate pathway and its synthesis of isopentenyl compounds. Several compounds have been shown to inhibit Dxr, and their use is geared toward treatment of bacterial and parasitic diseases, inclusive of tuberculosis and malaria [63]. (4) ß-ketoacyl acyl carrier protein reductase (FabG), also known as 3-oxoacyl-[acyl-carrier-protein] reductase. FabG catalyzes the reduction of beta-ketoacyl-acyl carrier (ACP) protein substrates by NADPH to beta-hydroxyacyl-ACP products, can also catalyze the reduction of acetoacetyl-CoA, albeit less efficiently than paralog proteins, and appears to play a role in fatty acid synthesis [64,65]. Initially characterized in *E. coli*, FabG1-4 orthologs are present in *M. tuberculosis* [66], and some have been shown to be inhibited by isoniazid [67], which is widely used in the treatment of tuberculosis. (5) Glucose-1-phosphate thymidylyltransferase (TYLT). TYLT catalyzes condensation of substrates glucose-1 phosphate and deoxy-thymidine triphosphate (dTTP), producing pyrophosphate and dTDP-d-glucose [68]. This is the first step in l-rhamnose synthesis, which in turn is a cell wall component of bacteria, inclusive of *M. tuberculosis* and *P. aeruginosa*. Thymine analogs developed against TYLT were surprisingly found to act through binding of an allosteric site [69], where current discovery efforts are focused [70], and not the active site, which is the pocket of interest in the current study. (6) 3-phosphoshikimate 1-carboxyvinyltransferase (EPSP synthase). EPSP catalyzes the condensation of 3-phosphoshikimate + phosphoenolpyruvate, producing 5-O-(1-carboxyvinyl)-3-phosphoshikimate + phosphate [71]. This constitutes an essential step in the shikimate pathway [72], present in bacteria through plant cells, but not animal cells, is used in the synthesis of folates and aromatic amino acids, and is targeted by the widely used herbicide, glyphosate [73].

### 3.2. Identification of Small-Molecule Ligands with the Potential to Interact with Binding Pockets

We next sought to identify small-molecule ligands that have the potential to interact with identified pockets using our computational pipeline for docking and scoring (Figure 1). Docking simulations were performed against a library of 60 million compounds, which were optimized for use in this pipeline and represent a subset of the ZINC compound library [74]. The library was first filtered for properties that have been associated with oral FDA-approved small-molecule therapeutics, inclusive of molecular weight between 160 and 480 g/mol and total polar surface area < 140 Å^2^ (i.e., <5 and <10 hydrogen-bond donors and acceptors, respectfully) [75]. These properties play a central role in defining a compound’s adsorption, distribution, metabolism, and excretion (ADME) characteristics, and thus its desirable pharmacokinetic profile [75]. Using the SurfaceScreen methodology, our pipeline initially incorporated AutoDock [32] and DOCK [33] docking applications, together providing a broad coverage of protein–small-molecule docking strategies, including accounting for protein residue and compound flexibility. Top-ranking initial docking poses were then ‘funneled’ into increasingly more sophisticated and computationally complex approximations of free energy of interaction using the MM-GBSA [34] and FEP/MD-GCMC [35] methods, with rescoring of poses conducted in a hierarchical fashion. In this manner, we created a ranked list of the top 100 compounds for each pocket (Figure S3).

**Figure 4:**
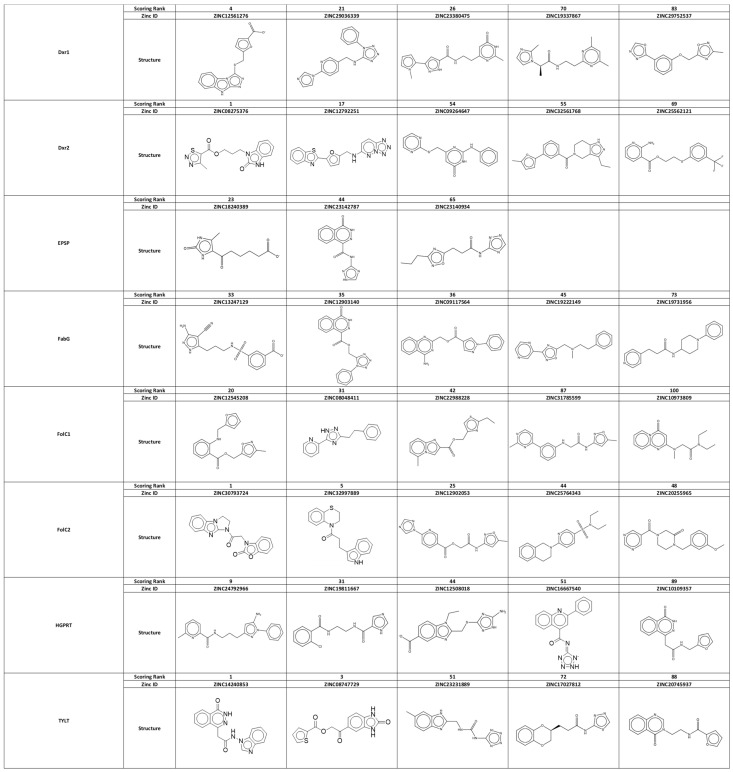
Structures of the acquired compounds.

From this ranked list, we selected three to five compounds for further testing. Compounds were then reviewed by experts in medicinal chemistry (K.S.) and in cancer experimental therapeutics (R.B.). Factors favoring selection included availability of compound for purchase (with purity > 90%), higher docking rank, absence of structural characteristics of concern for a therapeutic, such as liable under acid conditions and/or susceptibility to phase I metabolism, especially that due to cytochrome P450, and chemical diversity across compounds for each site. Factors contributing to exclusion included apparent close analogs to known anticancer agents and the possibility that the compound may interfere with a basic and critical biological process (i.e., would have general systemic toxicity). Close analogs of DNA bases represented a not uncommon example for exclusion. The resultant 38 acquired compounds are listed in Figure 4, with 5 each directed against Dxr1, Dxr2, FolC1, FolC2, PRT, TYLT, and FabG, and 3 against EPSP.

### 3.3. Screening for Therapeutic Efficacy

We sought to identify compounds that would inhibit the growth of cancer cells, but that also did so selectively. Inhibition of cancer cell growth is a highly sought after attribute of anticancer agents. This was assessed by measuring the ability of a compound to inhibit cell growth in eight-day colony formation assays and examining concentrations ranging from 0.017 to 40 µM. Breast, colon, lung, and prostate cancer are four of the most common types of cancer in the United States, are major causes of cancer cell death, and were examined [76]. Two cell lines for each cancer type were assessed: A549 (adenocarcinoma) and H226 (squamous carcinoma) for lung, MCF-7 (estrogen receptor (ER)-positive) and MDA-MB-231 (ER-negative) for breast, LNCaP (androgen receptor (AR)-positive) and PC3-M (AR-negative) for prostate, and HCT116 (wildtype p53, mutated KRAS) and HT29 (p53 mutant) for colon [20].

In parallel with measuring growth inhibition of cancer cell growth, we measured the effect on human hematopoietic stem cells. Hematopoietic stem cells reside in the bone marrow and are the source of mature cells present in blood [77]. Bone marrow is one of the most sensitive organs in the body to drug toxicity, and this is especially so for anticancer drugs, where bone marrow toxicity can approach 100% [78,79,80]. Bone marrow toxicity was measured by performing hematopoietic colony formation assays on human CD34+ stem cells. The stem cell nature of this assay permitted the assessment of trilineage colony formation: granulocytic (white blood cells), erythrocytic (red blood cells), and megakaryocytic (platelets), measured by the formation of CFU-GM, BFU-E, and CFU-GEMM colonies, respectively. Two measurements were taken. First, total colonies at eight days were measured, thereby matching the eight-day timeframe of cancer cell colony formation assays. Second, at fourteen days, individual colonies had differentiated, permitting quantification of each lineage.

**Figure 5:**
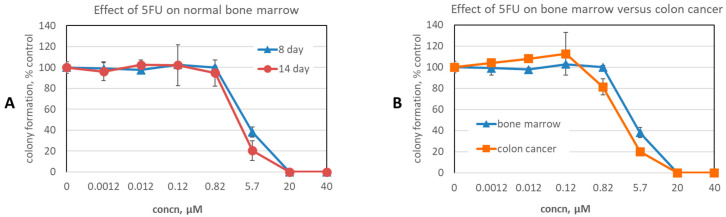
Effect of 5FU on normal bone marrow. (**A**) Effect of 5FU on eight- and fourteen-day human stem cell hematopoietic colony formation. (**B**) Effect of 5FU on eight-day colony formation for human stem cells and HT29 colon cancer cells. Data are the mean ± SEM of N = 4 and N = 2 replicates for stem and HT29 cells, respectively.

Data for all cancer cell and hematopoietic colony formation assays, across all compound concentrations tested, are provided in Figure S4. We first considered activity at 40 µM, the highest concentration tested. The effects on normal bone marrow, as measured by the eight-day stem cell colony formation assay, demonstrated that only six (16%) compounds inhibited colony formation by more than 10%, and none by more than 30% (Table 1). Bone marrow colony formation assays provide a measure of toxicity to stem cells and can be extended to fourteen days. If a compound was toxic to stem cells, expanded differences would be expected in the fourteen-day assay as compared to the eight-day assay. Interestingly, of the six compounds exerting >10% inhibitory effects at eight days, at fourteen days, recovery was observed with four compounds and partial recovery with one compound. The overall minimal effects of these compounds on bone marrow function were highlighted by considering the contrasting effect of 5-fluorouracil (5FU): 5FU is an antimetabolite anticancer agent in widespread clinical use and is considered to have only moderate bone marrow suppressive effects clinically [81]. However, 5FU completely suppressed colony formation at 40 µM, and still exerted profound suppressive effects even at concentrations 7-fold lower, i.e., 5.7 µM (Figure 5).

In considering the effect on cancer cells, for each compound and each cell line tested we calculated the efficacy ratio, i.e., efficacy ratio = (percent of remaining cancer cell colonies)/(percent of remaining bone marrow colonies), at 8 days after treatment with 40 µM of compound (Figure S5). We considered those with an efficacy ratio below 0.5 as active and at least partially selective. The stringency of this is highlighted by considering the effect of 5FU on colon cancer, where it is frequently used clinically in proven curative settings, as well as proven life-prolonging settings [81,82]. At a concentration where 5FU had no effect on bone marrow, i.e., 0.82 µM, the efficacy ratio for human colon cancer HT29 cells was 0.82 (Figure 5B). Even at 5.7 µM, where 5FU is toxic to bone marrow, with hematopoietic colony formation at 38% of the control, the efficacy ratio for HT29 cells only dropped to 0.52. In Table 2, we list all compounds exhibiting an efficacy ratio below 0.5 in at least one cell line. It can be seen that compounds segregated to protein sites. Specifically, of the 10 compounds meeting this activity criteria (i.e., efficacy ratio < 0.5), 3 were directed at the Dxr2 site, 3 at FolC2, 2 at FolC1, 1 at Dxr1, and 1 at TYLT.

Considered from the perspective of individual protein sites, our findings at 40 µM highlighted the importance of Dxr2 and FolC2 sites. As shown in Table 3 for Dxr2, 60% of tested compounds were considered active against at least one cell line. Of those rated active, the efficacy ratio was 0.06 ± 0.022 (mean ± SEM), and for active compounds, the percentage of cell lines exhibiting an efficacy ratio below 0.5 ranged from 71 to 100. For FolC2, 60% of tested compounds were active, the efficacy ratio was 0.15 ± 0.040 (mean ± SEM), and the percentage of susceptible cell lines ranged from 63 to 100.

**Figure 6:**
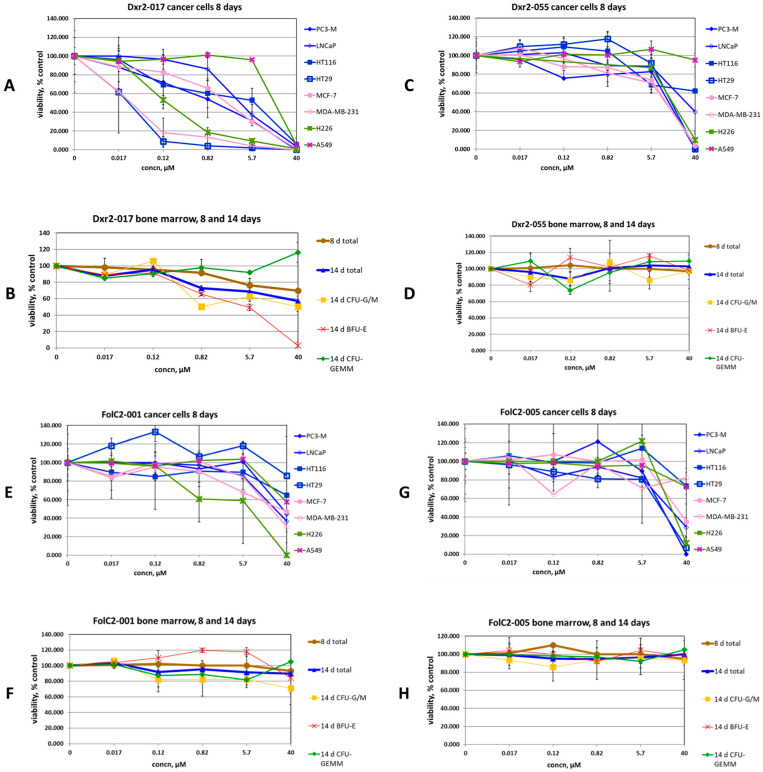
Effect of compounds on cancer cell and bone marrow colony formation. The effect of denoted compounds on eight-day cancer cell colony formation (**A**,**C**,**E**,**G**). The effect of denoted compounds on eight- and fourteen-day bone marrow colony formation (**B**,**D**,**F**,**H**). Data are mean ± SD (N = 2 replicates), with similar findings in separate experiments (also N = 2 replicates).

Examination of compound activity at the next lowest tested concentration of 5.7 µM further supported the importance of Dxr2-directed compounds, and of Dxr2-017 in particular (Table 4). Dxr2-017 at 5.7 µM exhibited an efficacy ratio below 0.5 in 75% of cells tested, with those below this cutoff having a 0.25 ± 0.091 (mean ± SEM) efficacy ratio. The activity of Dxr2-017 across all tested concentrations and all cells tested is shown in Figure 6A,B, as is its effect on eight- and fourteen-day bone marrow stem cell trilineage colony formation. At 0.12 µM, Dxr2-017 significantly inhibited HT29 colon cancer and MDA-MB-231 breast cancer colony formation by 91.1 ± 4.8% (mean ± SD) and 81.7 ± 15.5%, respectively, compared to the control. At concentrations of over 300-fold higher (i.e., 40 µM), Dxr2-017 only inhibited normal bone marrow eight-day colony formation by 30.2 ± 0.7%, and fourteen-day total colony formation by 42.6 ± 5.8%. The impact on trilineage colony formation could be measured at fourteen days. It demonstrated that at 40 µM, Dxr2-017 increased CFU-GEMM by 16.1 ± 12.0% compared to the control, while CFU-GM and BFU-E both decreased by 49.8 ± 0.0% and 97.1 ± 4.1%, respectively.

Depicted in Figure 6C–H are comprehensive data for three additional illustrative compounds, while the data for all compounds are shown in Figure S4. Compound Dxr2-055 (Figure 6C,D) was directed toward the Dxr2 site and, similar to Dxr2-017 at 40 µM, it demonstrated an efficacy ratio below 0.5 against several cancer cells, i.e., six cell lines, and had no inhibitory effect on normal bone marrow. However, unlike Dxr2-017, below 40 µM Dxr2-055 efficacy was completely lost. A similar phenomenon was observed with FolC2-005 (Figure 6G,H). With FolC2-001 (Figure 6E,F), it had the advantage of demonstrating a more dynamic dose–response profile, with five cell lines exhibiting an efficacy ratio below 0.5 at 40 µM and some degree of efficacy observed at concentrations below 40 µM. However, FolC2-001’s overall activity was not considered high. At 40 µM for cells where the efficacy ratio was below 0.5, FolC2-001 had an efficacy ratio of 0.29 ± 0.09 (mean ± SEM), which is 9.7-fold higher than that of Dxr2-017, which was 0.03 ± 0.01 (*p* = 0.001). Further, the activity of FolC2-001 rapidly dropped off below 40 µM. The profiles of other compounds (Figure S4) were of even lower interest, mostly based on low efficacy ratios. It is important to consider that this general finding in fact provides additional supportive data related to the selective efficacy of Dxr2-017.

### 3.4. Expansion of Efficacy Studies

**Figure 7:**
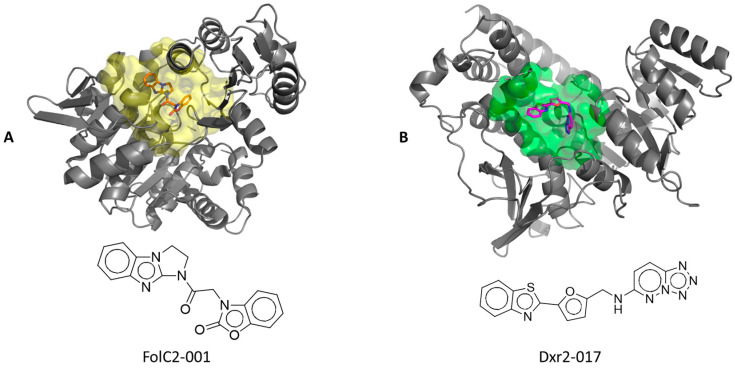
The predicted poses of bound Dxr2-017 and FolC2-001. The poses of FolC2-001 (**A**) and Dxr2-017 (**B**) bound to their respective FolC2 (yellow) and Dxr2 (green) binding surfaces are depicted, as are ribbon structures of proximal portions of the protein.

The above findings support the importance of Dxr2 and FolC2 sites, and of Dxr2-017 and FolC2-001 compounds, respectively. The predicted poses of Dxr2-017 bound to the Dxr2 site and FolC2-001 bound to the FolC2 site are shown in Figure 7. The NCI-60 cell line screen evaluates compound growth inhibition and cell toxicity in a highly standardized manner across 60 different cell lines. Dxr2-017 and FolC2-001 were evaluated in this assay (Figure S6). NCI-60 screens evaluate cells treated for 48 h at 10 µM with a given compound. Findings for both Dxr2-017 and FolC2-001 corroborated those in Figure 6, indicating Dxr2-017’s activity against HT29, MDA-MB-231, and H226, and FolC2-001 against H226. Of high interest, in the NCI-60 screen, Dxr2-017 had relatively high activity against melanoma cell lines, inducing cell death in five cell lines ranging from 25% to 65%, and inhibiting growth in four cell lines ranging from 76% to 96%. Its impact on cell death represented the most striking relative difference in response across cell lines.

**Figure 8:**
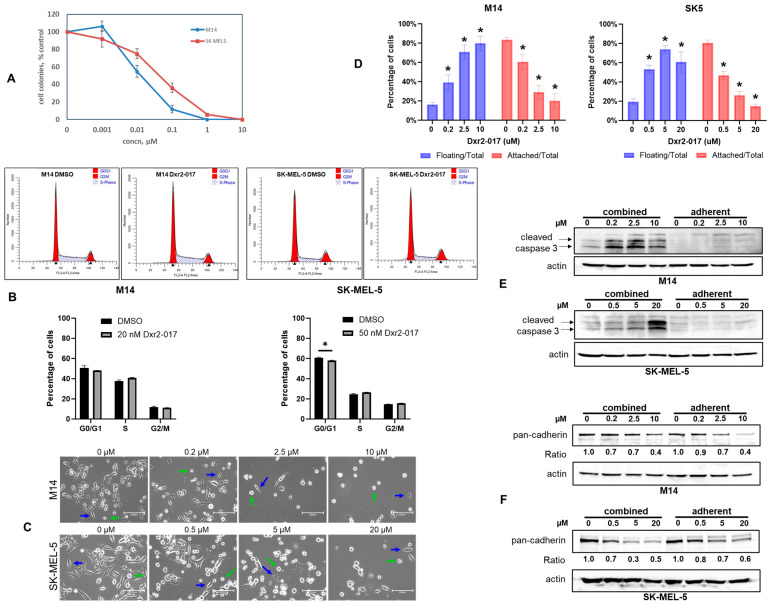
Dxr2-017 inhibits melanoma cell growth through induction of anoikis. (**A**) Inhibition of melanoma cell growth. Cells were treated with different concentrations of Dxr2-017, and formation of colonies at eight days is depicted. Data are expressed as the percent of untreated control and are the mean ± SEM of three separate experiments. Each experiment was conducted with N = 3 replicates. (**B**) No effect on cell cycle progression. M14 and SK-MEL-5 cells were treated for eight days with 20 nM and 50 nM of Dxr2-017, respectively, and the phase of the cell cycle was determined by flow cytometry. Control cells were treated with DMSO vehicle only. Representative histograms are depicted. Graphical data are the mean ± SEM (N = 3). * Denotes *p*-value ≤ 0.05. (**C**,**D**) Transition to floating cells. Cells were treated for three days with different Dxr2-017 concentrations. (**C**) Depicted are representative light photomicrographs at 20X. The scale bar is 150 µm. Blue and green arrows denote adherent and floating cells, respectively. (**D**) Adherent and floating cells were quantified from captured images. Data are the mean ± SEM (N = 4), expressed as a percentage to total cells. * Denotes *p*-value ≤ 0.05 compared to respective floating or attached cells. (**E**) Cleaved caspase 3 is induced in floating cells. Cells were treated for 3 days, and cell lysate from only adherent cells, or from adherent and floating cells combined, was probed by Western blot for cleaved caspase 3. (**F**) Dxr2-017 decreases cadherin. Cell lysate from only adherent cells or from adherent and floating cells combined was probed by Western blot for pan-cadherin. All experiments were repeated at separate times, yielding similar results. Original western blots are presented in File S2.

We next evaluated the effect of Dxr2-017 on human melanoma M14 and SK-MEL-5 cell lines in eight-day assays (Figure 8A). The IC50 values for Dxr2-017 were 12.0 ± 1.5 nM and 51.0 ± 11.0 nM (mean ± SEM) for M14 and SK-MEL-5, respectively. Compared to the eight-day treatment of bone marrow at 40 µM, where colony formation was only decreased by 30%, this translated to greater than 2100-fold efficacy for M14 and 750-fold for SK-MEL-5 cells.

We next investigated the mechanism by which Dxr2-017 inhibited cell growth. We first examined whether Dxr2-017 inhibited cell cycle progression (Figure 8B). Treatment of M14 and SK-MEL-5 cells with Dxr2-017 for eight days at concentrations closely matching their respective IC50 values demonstrated little-to-no change in cell cycle parameters.

We then assessed whether Dxr2-017 induced apoptosis. We treated M14 and SK-MEL-5 cells with Dxr2-017 for three days at concentrations of up 10 and 20 µM, respectively, and measured cleaved caspase 3 using Western blot. We did not see any induction of cleaved caspase 3 (Figure S7). In this assay, cells were grown in six-well plates and washed prior to lysis, and thus only adherent cells remained. These findings suggested that Dxr2-017 may induce anoikis, a process in which programmed cell death occurs upon cell detachment [13,14,15]. This was supported by demonstrating that Dxr2-017 induced cell detachment in a concentration- and time-dependent manner, while there were no morphological differences in adherent cells between treatment and control (Figure 8C,D and Figure S8). Adherent cells are spread out and fusiform shaped, while detached/floating cells are round, as previously reported by us [83]. Changes in this morphology provide a measure of a cell’s state of attachment. Specifically, under conditions of the 3-day treatment (Figure 8D), Dxr2-017 significantly increased the floating cell population in M14 cells from 16.3 ± 2.8% in controls up to 79.9 ± 7.4% in cells treated with 10 µM, and in SK-MEL-5 cells from 19.5 ± 3.2% in controls up to 60.8 ± 10.4% in cells treated with 20 µM. Effects were observed at all lower concentrations tested in both cell lines. Under conditions of the eight-day treatment, similar effects were evident at low nanomolar concentrations (Figure S8).

These findings led us to compare cleaved caspase 3 between adherent cells and floating cells. Cleaved caspase 3 is an indicator of programmed cell death, and its triggering by loss of cell attachment is an established means of detecting anoikis [84]. Adherent cells were prepared as above. To examine effects on floating cells, cells were grown under the exact same conditions as that for adherent cells, except that cells that washed away were collected, isolated by centrifugation, and the resultant lysate was combined with that of adherent cells. While there was no formation of cleaved caspase 3 with up to 10 µM or 20 µM Dxr2-017 in M14 or SK-MEL-5 cells, respectively, when only the adherent fraction was probed, in contrast, clear formation was observed when the floating cell fraction was included (Figure 8E). Several groups have implicated loss of cadherin expression with induction of anoikis across several different cancer types, inclusive of melanoma [16,85,86]. It can be seen in Figure 8F that Dxr2-017 decreased cadherin expression in a concentration-dependent manner in M14 and SK-MEL-5 cells. Together, these findings demonstrated that Dxr2-017 decreased cadherin expression, increased cell detachment, and induced programmed cell death upon cell detachment. All these processes are seen with induction of anokis.

## 4. Discussion

We demonstrated a proof-of-concept that 3D protein structures represent an important source of primary information that can be used to begin the process of drug discovery. We elected to use a protein structure library created by us, as well as a suite of analytic tools created by us. Finally, we elected to apply this strategy to the task of discovering anticancer agents that selectively inhibit cancer cell growth. The basic concept of recognizing that novel protein structures represent an information portal to novel function represents a rich strategy that is broadly adaptable. Compared to the library and analytic tools used herein by us, it is recognized that larger libraries are available and increasingly accessible and that exponential advances in protein computational analytics continue to emerge [87,88,89,90,91,92]. These libraries and emerging tools can be used to apply this proof-of-concept to applications that range across the spectrum of the most basic biology to an array of given end-use functions.

Our demonstration that compounds selected as active in the current study segregated to specific protein pockets is of high importance. Our current strategy used a physically defined protein structure, and this was coupled to experimentally determined measures of function. However, the intermediate process of compound screening was virtual. That our functional measures of activity segregated to protein pockets provides an important measure of the real-world utility of our computational approaches, as applied to virtual screening.

Further, we consider our integrated and layered screening approach to be of high value. The first layer in this approach used upfront sets of computational analytics. The second layer transitioned to experimental analytics and involved in-parallel selection and de-selection screens. The latter is a strategy previously employed by us, which was also highly successful [20]. At this point, our analytics yielded priority compounds, with Dxr2-017 emerging as a clear lead. Our incorporation at this stage of a broader screening layer, i.e., the NCI-60 cell line screen, further solidified the lead and served to redirect our disease focus to melanoma.

Our demonstration that Dxr2-017 was potent, specific, and acting through a novel mechanism represented three individual factors that independently, and certainly together, supported our hypothesis that unique protein structures provide a high-value entry point for the downstream discovery of novel therapeutics. Specifically, IC50 values in the low nanomolar range highlighted the potency of Dxr2-017. That concentrations over 2100-fold higher had little effect on bone marrow stem cells provides a powerful measure of specificity. A comprehensive examination of all organ systems in the context of a systemic model over an extended time is necessary to provide a more exhaustive assessment of specificity. However, considering the almost universally high sensitivity of bone marrow to anticancer agents, and the magnitude of an over 2100-fold therapeutic window, high specificity is well supported. The novelty of Dxr2-017 is supported by its induction of anoikis. Although anoikis has long been recognized as an important cellular process, there is a limited understanding of its regulation, and a great need for therapeutics that specifically induce it [13,14,15,16]. Findings from the current study provide a new means to specifically target anoikis. Further, the small chemical nature of Dxr2-017 also represents a new tool that can be used as a probe to interrogate biology. It is recognized that large knowledge gaps exist with respect to further advancing Dxr2-017 along a drug development pathway. A major one relates to its actual target(s). In this regard, it should be noted that the Dxr2 pocket is part of the protein 1-deoxy-D-xylulose 5-phosphate reductoisomerase, also known as DXP reductoisomerase, and represents a protein not expressed in humans.

There are several limitations in the current study. Our selection methodology of proteins to be analyzed relied heavily on human-based selection factors. Our virtual analytics did not employ newer artificial-intelligence-based techniques. While such cutting-edge techniques have clear potential to add, they do need to be viewed with caution [87,93]. Further, the number of chemicals subjected to experimental analysis was small. While this latter factor could also be considered a measure of efficiency, high-throughput methods would likely serve to be at least additive. Although our approach of considering phylogenetic preservation of structure has a justifiable rational basis, it was not possible within the current study to make any strong attributive statements as to its overall importance. Our findings could be considered as providing additional evidence to support the hypothesis that such considerations are important, at least as they relate to identifying sought after therapeutic effects. Current findings do support future investigations probing this notion.

This study provided a proof-of-concept that 3D protein structures represent an important starting point from which to launch the drug discovery process. The basic nature of this strategy has wider implications related to application and biology. Dxr2-017 was identified as a highly active compound that inhibits the growth of human melanoma cells, has a minimal impact on human bone marrow, and induces anoikis. It holds high promise as a therapeutic, or for informing the development of such.

## 5. Conclusions

We provided a proof-of-concept demonstration that 3D protein structures, agnostic of function, can be probed to identify structures deemed unique, and that such unique structures constitute high-value information that can then drive discovery of novel acting compounds. In this manner, we identified one compound, Dxr2-017, that inhibited growth of human melanoma cells at low nanomolar concentrations. We further demonstrated that concentrations of over 2100-fold higher had minimal effects on human bone marrow stem cells. Considering that bone marrow stem cells are highly susceptible to anticancer drug toxicity and that a 2100-fold therapeutic index is very high, these findings portend a high impact potential for Dxr2-017 as an effective therapeutic in humans. Dxr2-017 was shown to induce anoikis. This is an important mechanism for a cancer therapeutic because resistance to anoikis permits cell survival after cell detachment, facilitating movement of cancer cells throughout the body.

## Figures and Tables

**Table 1:** Effects of compounds on normal bone marrow.

	Mean Number of Colonies, % of Control *									
Compound	Dxr2-001	Dxr2-017	Dxr2-054	Dxr2-055	Dxr2-069	FolC2-001	FolC2-005	FolC2-025	FolC2-044	FolC2-048
8 days	109	70	79	99	108	108	105	93	96	87
14 days	93	57	112	115	75	87	101	109	73	120
Compound	FolC1-020	FolC1-031	FolC1-042	FolC1-087	FolC1-100	Dxr1-005	Dxr1-021	Dxr1-026	Dxr1-070	Dxr1-083
8 days	107	80	105	107	98	106	101	100	96	97
14 days	97	85	102	94	96	90	80	128	97	93
Compound	TYLT-001	TYLT-003	TYLT-051	TYLT-072	TYLT-088	FabG-033	FabG-035	FabG-036	FabG-045	FabG-073
8 days	117	97	84	88	101	102	113	106	97	97
14 days	103	94	103	104	112	94	105	100	103	92
Compound	PRT-009	PRT-031	PRT-044	PRT-051	PRT-P89	EPSP-023	EPSP-044	EPSP-065		
8 days	111	106	101	93	111	94	98	99		
14 days	101	97	93	119	93	114	98	98		

* Values are mean (N = 2 replicates). Blue font denotes >10% decrease compared to control.

**Table 2:** Compounds with desirable efficacy ratios at 40 µM.

	Ratio of Cancer Cell Colonies to Bone Marrow Colonies at 8 Days *									
Cell Line	Dxr2-017	Dxr2-055	Dxr2-069	FolC2-001	FolC2-005	FolC2-044	FolC1-042	FolC1-020	Dxr1-021	TYLT-072
HT116	0.08	0.63	0.93	0.60	0.70	0.03	0.51	0.23	0.95	1.18
HT29	0.00	0.00	0.01	0.79	0.06	0.00	0.10	0.12	0.59	2.05
MCF-7	0.02	0.04	0.03	0.43	0.33	0.01	0.64	0.01	0.81	0.98
MDAMB231	0.00	0.05	0.59	0.28	0.78	0.16	0.65	0.65	0.62	0.97
H226	0.02	0.10	0.03	0.00	0.11	0.00	0.01	0.26	1.26	0.14
A549	0.07	0.96	0.15	0.53	0.69	0.15	0.96	0.89	0.72	1.17
PC3-M	0.00	0.00	0.00	0.41	0.00	0.00	0.18	0.13	0.38	0.99
LNCaP **	0.05	0.40	-	0.34	0.28	-	-	-	-	-
Relative activity for a given compound, % ¥	100	75	71	63	63	100	43	73	14	14

* Efficacy ratios of <0.5 are denoted in blue font. ** LNCaP cells were only examined as determined by additional supporting data, as denoted. ¥ denotes the % of cells tested where the ratio was <0.5.

**Table 3:** Compound characteristics at each site *.

		Activity **		
Site	Active Compounds, %	Mean	SEM	Range, % ¥
Dxr2	60	0.06	0.02	71–100
FolC2	60	0.15	0.04	63–100
FolC1	40	0.13	0.03	43–73
Dxr1	20	0.38	0	14
TYLT	20	0.14	0	14
PRT	0	NA	NA	NA
EPSP	0	NA	NA	NA
FabG	0	NA	NA	NA

* Compounds at 40 µM. ** For compounds with efficacy ratio < 0.5, the mean ± SEM ratio is shown. ¥ For compounds with efficacy ratio < 0.5 for a given site, the percentage of cell lines it was active against was calculated and used to determine the range.

**Table 4:** Efficacy ratios at 5.7 µM of compound.

	Ratio of Cancer Cell Colonies to Bone Marrow Colonies at 8 Days *			
Cell Line	Dxr2-017	FolC2-001	FolC1-020	TYLT-072
HT116	0.69	0.85	0.88	0.83
HT29	0.03	1.13	0.69	1.22
MCF-7	0.39	0.65	0.08	0.94
MDAMB231	0.05	0.81	0.96	0.95
H226	0.12	0.56	0.96	0.26
A549	1.26	0.99	0.84	0.99
PC3-M	0.41	0.96	0.76	1.02
LNCaP **	0.49	0.82	-	-
Relative activity for a given compound, % ¥	75	13	14	14

* Ratios of <0.5 are denoted in blue font. ** LNCaP cells were only examined as determined by additional supporting data, as denoted. ¥ denotes the % of cells tested where the ratio was <0.5.

## Data Availability

Protein structure data presented in this study are available from the public domain resource, the Protein Data Bank (PDB), at: wwpdb.org. All other data are contained within the article or Supplementary Materials.

## Supplementary Materials

The following supporting information can be downloaded at: https://www.mdpi.com/article/10.3390/cancers16183177/s1. Figure S1. Source of compounds. Figure S2. List of Protein Crystal Structures Examined. The first 220 crystal structures deposited into the Protein Database (PDB) from the Center for Structural Genomics of Infectious Diseases (CSGID) are listed. Figure S3. List of Top 100 Compounds Predicted to Bind in Each Pocket. Figure S4. Effect of Compounds on Cancer Cell Growth and Hematopoietic Stem Cell Toxicity. For human cancer cell lines, eight-day colony formation assays were conducted. For CD34+ human cord blood hematopoietic stem cells, at eight days the total number of colonies were counted. At fourteen days, the number of colonies in the following hematopoietic lineages were counted: granulocytic (white blood cells), erythrocytic (red blood cells) and megakaryocytic (platelets), measured by the formation of CFU-GM, BFU-E and CFU-GEMM colonies, respectively. All assays were run in replicates of N = 2, data expressed as the mean ± SD percentage of control. Initial findings meeting stipulated criteria were repeated, also in replicates of N = 2. For initial screening, cells were treated with 0 (vehicle), 0.017, 0.12, 0.82, 5.7 and 40 μM of the denoted compound. For replicate experiments, cancer cells were treated with 0 (vehicle), 0.0061, 0.018, 0.05, 0.16, 0.49, 1.48, 4.4, 13.3 and 40 μM of the denoted compound. For cancer cells, compound was added one day after plating. For hematopoietic assays, compound was introduced into semisolid media on the day of plating. Figure S5. The Efficacy Ratio of All Compounds and All Cancer Cell Lines Tested. Cancer cells and human bone marrow stem cells were treated with the indicated compound at 40 μM, and the resultant efficacy ratio is shown. The efficacy ratio = (percent of remaining cancer cell colonies)/(percent of remaining bone marrow colonies), at 8 days after treatment. Figure S6. NCI-60 cell line screens. COMPARE plots for Dxr2-017 and FolC2-001 are shown. Figure S7. Dxr2-017 did not induce apoptosis in adherent cells. M14 or SK-MEL-5 cells were plated onto 6 well plates, and 24 hours later they were treated with the denoted concentrations of Dxr2-017. After 3 days, cells were washed with PBS, the remaining adherent cells were lysed and resultant lysate was probed by Western blot for cleaved caspase 3. The depicted cleaved caspase 3 Western blots were exposed for a long period of time, as denoted by the evident high background signal. Figure S8. Dxr2-017 increases floating cells and does not alter adherent cell morphology. Cells were treated for eight days with Dxr2-017 at concentrations close to the IC50 value for each cell line examined. Depicted are representative light photomicrographs at 20X; scale bar is 150 μm. Green arrows denote floating cells. File S2: Original western blots.
