# Supplementary material for: Protein Structure Inspired Discovery of a Novel Inducer of Anoikis in Human Melanoma
Source: Cancers (Basel). 2024 Sep 17;16(18):3177. doi: 10.3390/cancers16183177 (PMC11429909; doi:10.3390/cancers16183177)
Supplement: Supplementary file 1 [file cancers-16-03177-s001.zip › cancers-3189012-File S2.pdf]

Pictures of the whole images for blots shown in manuscript Fig 8E: In conducting this Western blot, after transfer the nitrocellulose membrane was physically cut, allowing each cut section to be probed initially for cleaved caspase 3 or actin, as denoted. This was done for M14 cells, giving Membrane A, which was then cut as described. This was also done for SK-MEL-5 cells, giving Membrane B, which was then cut as described.

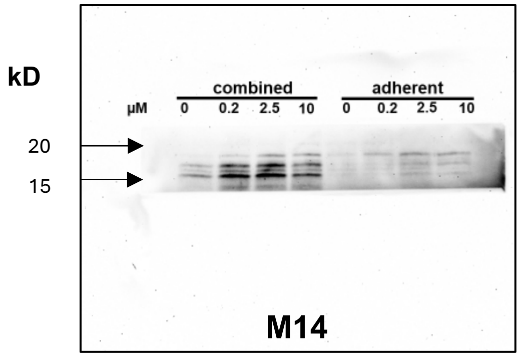

cleaved caspase 3
(17&19 kDa)

Membrane A

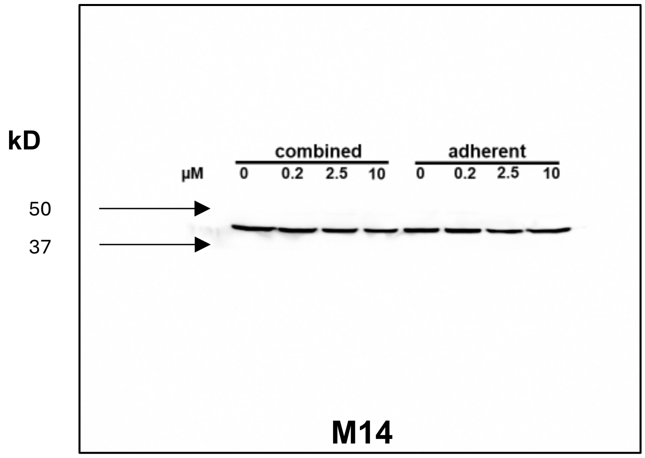

actin (43 kDa)

Membrane A

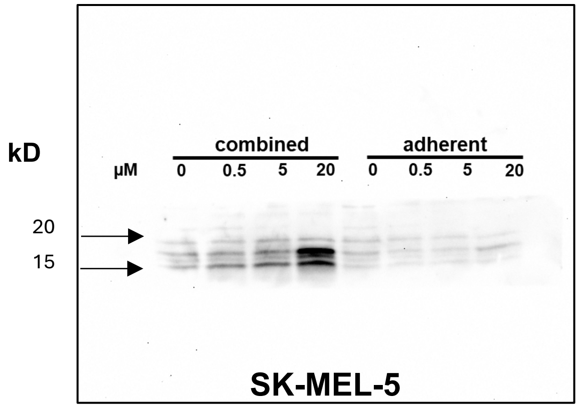

cleaved caspase 3
(17&19 kDa)

Membrane B

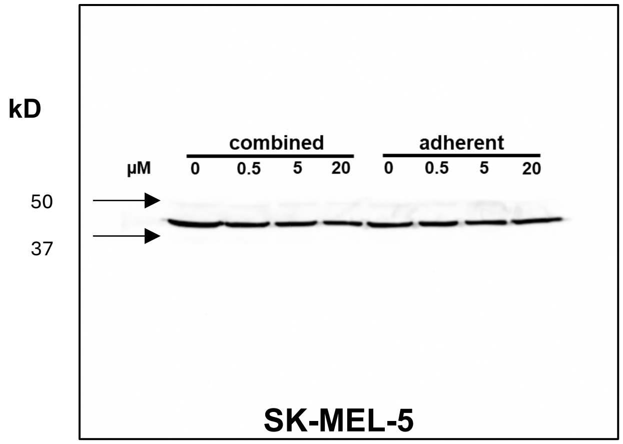

actin (43 kDa)

Membrane B

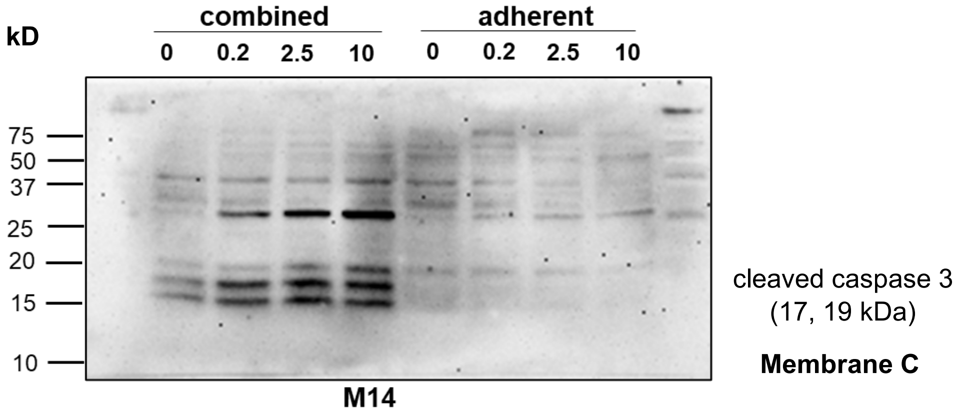

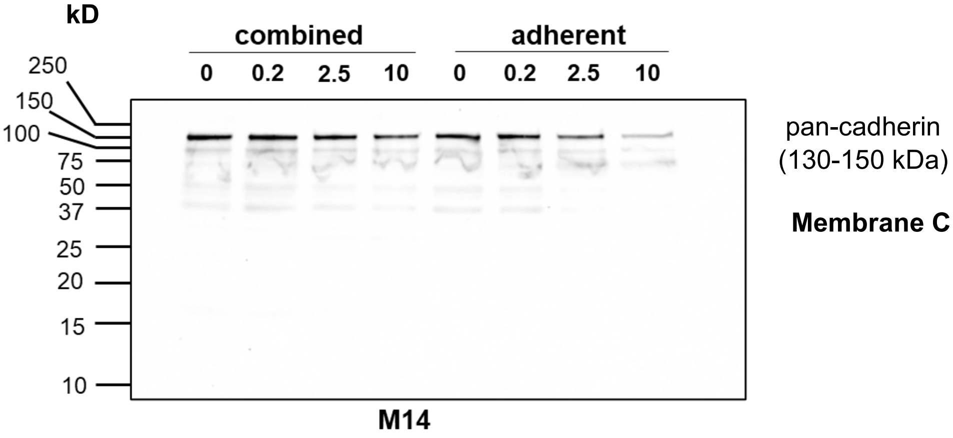

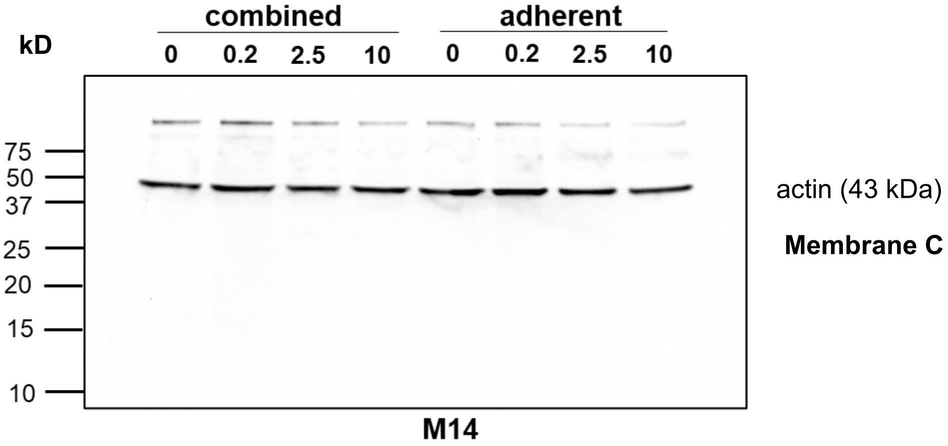

Pictures of whole blots for a repeat experiment of Figure 8E for M14 cells. This experiment was conducted as described for Fig 8E (above) except this time the membranes were not physically cut, as they were above. This allowed us to depict the entire, uncut, membrane. For M14 cells protein was transferred onto Membrane C, which was first probed for cleaved caspase 3 (CC3). Because the CC3 signal was so low, we did not strip the membrane before probing for pan-cadherin. Similarly, as actin signal was higher still, the membrane was then probed for actin.

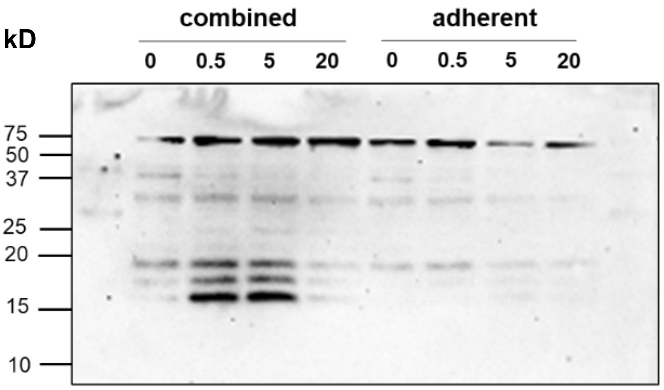

SK-MEL-5

cleaved caspase 3 (17, 19 kDa)

Membrane D

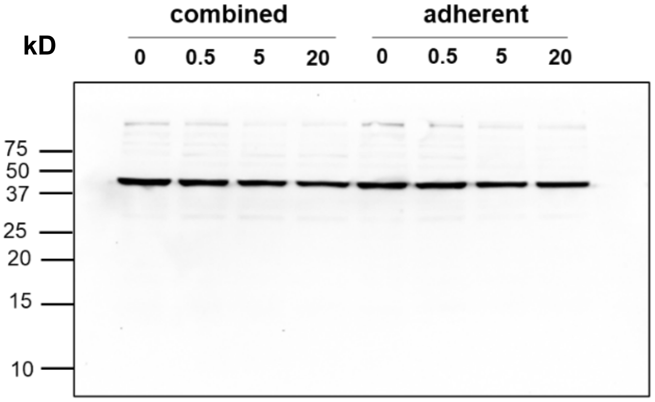

SK-MEL-5

actin (43 kDa)

Membrane D

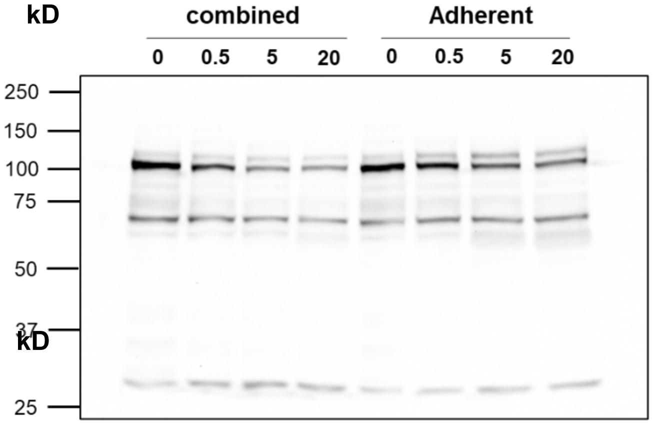

SK-MEL-5

pan-cadherin (130-150 kDa)

Membrane E

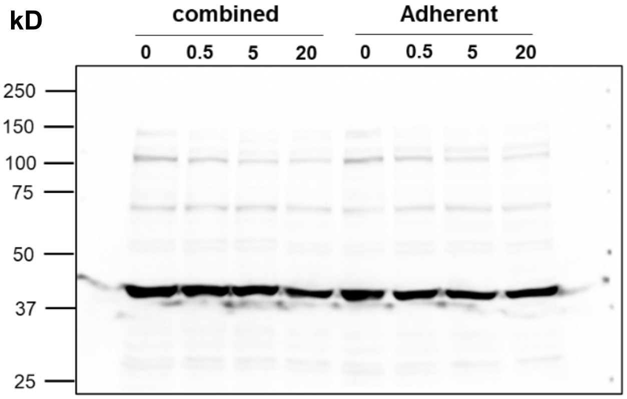

SK-MEL-5

actin (43 kDa)

Membrane E

Pictures of whole blots for a repeat experiment of Figure 8E for SK-MEL-5 cells. This experiment was conducted as described for Fig 8E (above) except this time the membranes were not physically cut, as they were above. This allowed us to depict the entire, uncut, membrane. For SK-MEL-5 cells, two separate gels were run, resulting in two separate membranes: Membrane D and Membrane E. Membrane D was first probed for cleaved caspase 3 (CC3), and was then probed for actin. Membrane E was first probed for pan-cadherin and was then probed for actin.

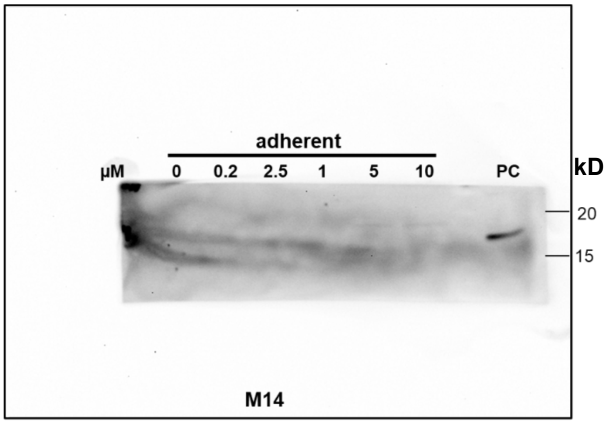

cleaved caspase 3 (17, 19 kDa)

Membrane F

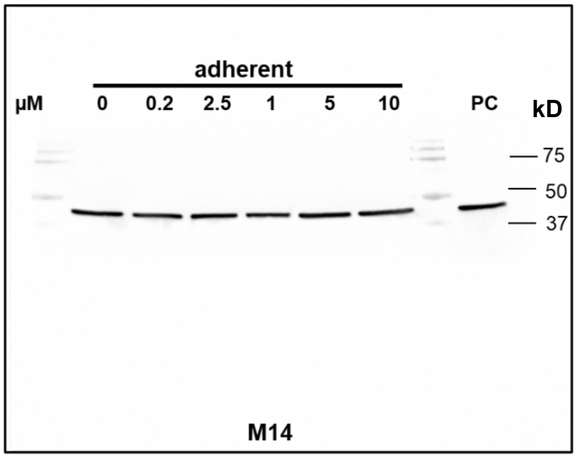

actin (43 kDa)

Membrane F

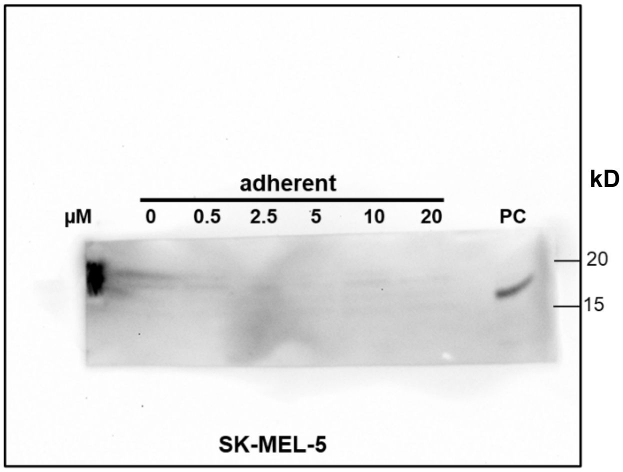

cleaved caspase 3 (17, 19 kDa)

Membrane G

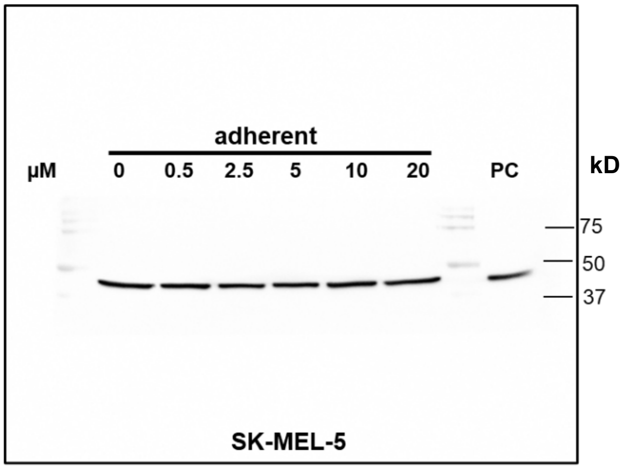

actin (43 kDa)

Membrane E

Pictures of whole images of blots for Supplementary figure 7 (S7). In conducting this Western blot, after transfer the nitrocellulose membrane was physically cut, allowing each cut section to be probed initially for cleaved caspase 3 or actin, as denoted. This was done for M14 cells, generating Membrane F, which was physically cut as described above. This was done for SK-MEL-5 cells, generating Membrane G, which was physically cut as described above.

NOTE: PC denotes positive control. This represents RAW264.7 cells treated with zoledronic acid for 72 hours. When RAW264.7 cells are treated with RANK ligand, as they were here, they differentiate into mature osteoclasts (OCs). ZA is known to induce cleaved caspase 3 in OCs. Thus, lysate from these cells was used as a positive control.
