# Supplementary material for: Protein Structure Inspired Discovery of a Novel Inducer of Anoikis in Human Melanoma
Source: Cancers (Basel). 2024 Sep 17;16(18):3177. doi: 10.3390/cancers16183177 (PMC11429909; doi:10.3390/cancers16183177)
Supplement: Supplementary file 1 [file cancers-16-03177-s001.zip › cancers-3189012-supplementary.pdf]

Supplementary Table. Source of compounds.		
ZINC ID	manufacturer	Supplier code
ZINC08275376	Enamine	T6279421
ZINC12561276	Enamine	T5944664
ZINC32561768	ChemBridge	57193933
ZINC09264647	ChemBridge	9131223
ZINC23380475	ChemBridge	54727150
ZINC19337867	ChemBridge	52123667
ZINC25562121	Enamine	T5809190
ZINC29752537	Enamine	T6206758
ZINC29036339	Enamine	T6497726
ZINC12792251	Enamine	T5999444
ZINC12903140	Enamine	T5953901
ZINC08048411	ChemBridge	9041543
ZINC19731956	Enamine	T6730351
ZINC19222149	ChemBridge	46963950
ZINC18240389	ChemBridge	5910078
ZINC09117564	Enamine	T5748403
ZINC23142787	Enamine	T6334948
ZINC23140934	Enamine	T6365385
ZINC12545208	Enamine	T6162524
ZINC22988228	Enamine	T6354799
ZINC13247129	Enamine	T6657138
ZINC31785599	Enamine	T6596653
ZINC10973809	Enamine	T6029204
ZINC20255965	ChemBridge	75179884
ZINC16667540	ChemBridge	5532994
ZINC19811667	ChemBridge	64400086
ZINC12508018	Enamine	T5250237
ZINC20745937	ChemBridge	9195953
ZINC23231889	ChemBridge	30678022
ZINC17027812	ChemBridge	7929726
ZINC32997889	Enamine	T6232937
ZINC25764343	Enamine	T5592228
ZINC10109357	Enamine	T5675064
ZINC14240853	Enamine	T5879322
ZINC30793724	Enamine	T6499332
ZINC12902053	Enamine	T6105504
ZINC24792966	Enamine	T6660640
ZINC08747729	Enamine	T5798238

Figure S1. Source of compounds.

Supplementary Table. List of protein crystal structures examined

order	protein database ID	CSGID	resolution	Title*
1	PDB 3E4F (PDBSUM) (Profunc) (GPSS) (MMDB)	Target IDP00044	Resolution: 2Å	Title: Crystal structure of BA2930 - a putative aminoglycoside N3-acetyltransferase from Bacillus anthracis
2	PDB 3DZC (PDBSUM) (Profunc) (GPSS) (MMDB)	Target IDP00152	Resolution: 2.35Å	Title: 2.35 Angstrom resolution structure of WecB (VC0917), a UDP-N-acetylglucosamine 2-epimerase from Vibrio cholerae
3	PDB 3DR6 (PDBSUM) (Profunc) (GPSS) (MMDB)	Target IDP00086	Resolution: 1.75Å	Title: Structure of YncA, a putative acetyltransferase from Salmonella typhimurium
4	PDB 3DR8 (PDBSUM) (Profunc) (GPSS) (MMDB)	Target IDP00086	Resolution: 1.95Å	Title: Structure of YncA, a putative acetyltransferase from Salmonella typhimurium with its cofactor acetyl-CoA
5	PDB 3DR3 (PDBSUM) (Profunc) (GPSS) (MMDB)	Target IDP00107	Resolution: 2Å	Title: Structure of IDP00107, a potential N-acetyl-gamma-glutamylphosphate reductase from Shigella flexneri
6	PDB 3DQQ (PDBSUM) (Profunc) (GPSS) (MMDB)	Target IDP00121	Resolution: 2.7Å	Title: The crystal structure of the putative tRNA synthase from Salmonella typhimurium LT2
7	PDB 3CWC (PDBSUM) (Profunc) (GPSS) (MMDB)	Target IDP00122	Resolution: 2.23Å	Title: Crystal structure of putative glycerate kinase 2 from Salmonella typhimurium LT2
8	PDB 3FPI (PDBSUM) (Profunc) (GPSS) (MMDB)	Target IDP00520	Resolution: 2.8Å	Title: CRYSTAL STRUCTURE OF 2-C-METHYL-D-ERYTHRITOL 2,4-CYCLODIPHOSPHATE SYNTHASE ISPF COMPLEXED WITH CYTIDINE TRIPHOSPHATE
9	PDB 3FPK (PDBSUM) (Profunc) (GPSS) (MMDB)	Target IDP01074	Resolution: 1.7Å	Title: CRYSTAL STRUCTURE OF FERREDOXIN-NADP REDUCTASE FROM SALMONELLA TYPHIMURIUM
10	PDB 3FOB (PDBSUM) (Profunc) (GPSS) (MMDB)	Target IDP00046	Resolution: 1.74Å	Title: Crystal Structure of Bromoperoxidase from Bacillus anthracis
11	PDB 3FF1 (PDBSUM) (Profunc) (GPSS) (MMDB)	Target IDP00736	Resolution: 1.65Å	Title: Structure of Glucose 6-phosphate Isomerase from Staphylococcus aureus
12	PDB 3F6M (PDBSUM) (Profunc) (GPSS) (MMDB)	Target IDP00520	Resolution: 2.96Å	Title: Crystal structure of 2-C-methyl-D-erythritol 2,4-cyclodiphosphate synthase IspF from Yersinia pestis
13	PDB 3F4N (PDBSUM) (Profunc) (GPSS) (MMDB)	Target IDP00439	Resolution: 2.4Å	Title: Crystal Structure of Pyridoxal Phosphate Biosynthetic Protein PdxJ from Yersinia pestis
14	PDB 3F0I (PDBSUM) (Profunc) (GPSS) (MMDB)	Target IDP01300	Resolution: 1.88Å	Title: Arsenate reductase from Vibrio cholerae
15	PDB 3ETF (PDBSUM) (Profunc) (GPSS) (MMDB)	Target IDP01530	Resolution: 1.85Å	Title: Crystal structure of a putative succinate-semialdehyde dehydrogenase from Salmonella typhimurium LT2
16	PDB 3ERP (PDBSUM) (Profunc) (GPSS) (MMDB)	Target IDP01002	Resolution: 1.55Å	Title: Structure of IDP01002, a putative oxidoreductase from and essential gene of Salmonella typhimurium
17	PDB 3EGJ (PDBSUM) (Profunc) (GPSS) (MMDB)	Target IDP01334	Resolution: 2.9Å	Title: N-acetylglucosamine-6-phosphate deacetylase from Vibrio cholerae
18	PDB 3EFV (PDBSUM) (Profunc) (GPSS) (MMDB)	Target IDP01530	Resolution: 1.9Å	Title: Crystal structure of a putative succinate-semialdehyde dehydrogenase from Salmonella typhimurium LT2 with bound NAD
19	PDB 3EFE (PDBSUM) (Profunc) (GPSS) (MMDB)	Target IDP01712	Resolution: 2.3Å	Title: The crystal structure of the thiJ/pfpI family protein from Bacillus anthracis
20	PDB 3EFB (PDBSUM) (Profunc) (GPSS) (MMDB)	Target IDP01531	Resolution: 2.001Å	Title: Crystal structure of probable sor operon regulator from Shigella flexneri
21	PDB 3EER (PDBSUM) (Profunc) (GPSS) (MMDB)	Target IDP01325	Resolution: 1.45Å	Title: High resolution structure of a putative organic hydroperoxide resistance protein from Vibrio cholerae O1 biovar eltor str. N16961
22	PDB 3EEV (PDBSUM) (Profunc) (GPSS) (MMDB)	Target IDP01350	Resolution: 2.61Å	Title: Crystal Structure of Chloramphenicol Acetyltransferase VCA0300 from Vibrio cholerae O1 biovar eltor
23	PDB 3EDN (PDBSUM) (Profunc) (GPSS) (MMDB)	Target IDP00051	Resolution: 1.5Å	Title: Crystal structure of the Bacillus anthracis phenazine biosynthesis protein, PhzF family
24	PDB 3ECT (PDBSUM) (Profunc) (GPSS) (MMDB)	Target IDP01515	Resolution: 2.51Å	Title: Crystal Structure of the Hexapeptide-Repeat Containing-Acetyltransferase VCA0836 from Vibrio cholerae
25	PDB 3EC6 (PDBSUM) (Profunc) (GPSS) (MMDB)	Target IDP01540	Resolution: 1.6Å	Title: Crystal structure of the General Stress Protein 26 from Bacillus anthracis str. Sterne
26	PDB 3E9A (PDBSUM) (Profunc) (GPSS) (MMDB)	Target IDP01337	Resolution: 1.8Å	Title: Crystal structure of 2-dehydro-3-deoxyphosphooctonate aldolase from Vibrio cholerae O1 biovar eltor str. N16961
27	PDB 3E7N (PDBSUM) (Profunc) (GPSS) (MMDB)	Target IDP00079	Resolution: 2.45Å	Title: Crystal structure of D-ribose high-affinity transport system from Salmonella typhimurium LT2
28	PDB 3GRI (PDBSUM) (Profunc) (GPSS) (MMDB)	Target IDP00795	Resolution: 2Å	Title: The Crystal Structure of a Dihydroorotase from Staphylococcus aureus
29	PDB 3GOS (PDBSUM) (Profunc) (GPSS) (MMDB)	Target IDP00571	Resolution: 1.8Å	Title: The crystal structure of 2,3,4,5-tetrahydropyridine-2-carboxylate N-succinyltransferasefrom Yersinia pestis CO92
30	PDB 3GOA (PDBSUM) (Profunc) (GPSS) (MMDB)	Target IDP01071	Resolution: 1.7Å	Title: Crystal structure of the Salmonella typhimurium FadA 3-ketoacyl-CoA thiolase
31	PDB 3GO9 (PDBSUM) (Profunc) (GPSS) (MMDB)	Target IDP00573	Resolution: 1.62Å	Title: Predicted insulinase family protease from Yersinia pestis.
32	PDB 3GJZ (PDBSUM) (Profunc) (GPSS) (MMDB)	Target IDP00038	Resolution: 2.1Å	Title: CRYSTAL STRUCTURE OF MICROCIN IMMUNITY PROTEIN MCCF FROM BACILLUS ANTHRACIS STR. AMES
33	PDB 3GIU (PDBSUM) (Profunc) (GPSS) (MMDB)	Target IDP00836	Resolution: 1.25Å	Title: 1.25 ANGSTROM CRYSTAL STRUCTURE OF PYRROLIDONE-CARBOXYLATE PEPTIDASE (PCP) FROM STAPHYLOCOCCUS AUREUS
34	PDB 3GHZ (PDBSUM) (Profunc) (GPSS) (MMDB)	Target IDP01038	Resolution: 2.03Å	Title: 2-C-methyl-D-erythritol 2,4-cyclodiphosphate synthase from Salmonella typhimurium.
35	PDB 3GEU (PDBSUM) (Profunc) (GPSS) (MMDB)	Target IDP00851	Resolution: 1.9Å	Title: Crystal structure of IcaR from Staphylococcus aureus, a member of the tetracycline repressor protein family
36	PDB 3GE1 (PDBSUM) (Profunc) (GPSS) (MMDB)	Target IDP00743	Resolution: 2.7Å	Title: 2.7 Angstrom Crystal Structure of Glycerol Kinase (glpK) from Staphylococcus aureus in Complex with ADP and Glycerol
37	PDB 3GC2 (PDBSUM) (Profunc) (GPSS) (MMDB)	Target IDP00994	Resolution: 1.85Å	Title: 1.85 Angstrom Crystal Structure of O-succinylbenzoate Synthase from Salmonella typhimurium in Complex with Succinic Acid
38	PDB 3GBX (PDBSUM) (Profunc) (GPSS) (MMDB)	Target IDP01011	Resolution: 1.8Å	Title: Serine hydroxymethyltransferase from Salmonella typhimurium
39	PDB 3GA7 (PDBSUM) (Profunc) (GPSS) (MMDB)	Target IDP00896	Resolution: 1.55Å	Title: 1.55 Angstrom Crystal Structure of an Acetyl Esterase from Salmonella typhimurium
40	PDB 3G48 (PDBSUM) (Profunc) (GPSS) (MMDB)	Target IDP01112	Resolution: 1.5Å	Title: Crystal structure of chaperone CsaA form Bacillus anthracis str. Ames
41	PDB 3G1Z (PDBSUM) (Profunc) (GPSS) (MMDB)	Target IDP01693	Resolution: 1.95Å	Title: STRUCTURE OF IDP01693/YJEA, POTENTIAL T-RNA SYNTHETASE FROM SALMONELLA TYPHIMURIUM
42	PDB 3G25 (PDBSUM) (Profunc) (GPSS) (MMDB)	Target IDP00743	Resolution: 1.9Å	Title: 1.9 ANGSTROM CRYSTAL STRUCTURE OF GLYCEROL KINASE (GLPK) FROM STAPHYLOCOCCUS AUREUS IN COMPLEX WITH GLYCEROL
43	PDB 3G0M (PDBSUM) (Profunc) (GPSS) (MMDB)	Target IDP00875	Resolution: 1.76Å	Title: Crystal structure of cysteine desulfuration protein SufE from Salmonella typhimurium LT2
44	PDB 3G0S (PDBSUM) (Profunc) (GPSS) (MMDB)	Target IDP01004	Resolution: 1.85Å	Title: Dihydrodipicolinate synthase from Salmonella typhimurium LT2.
45	PDB 3FWW (PDBSUM) (Profunc) (GPSS) (MMDB)	Target IDP00427	Resolution: 2.5Å	Title: The crystal structure of the bifunctional N- acetylglucosamine-1-phosphate Uridyltransferase/Glucosamine-1-Phosphate acetyltransferase from Yersinia pestis CO92
46	PDB 3FWX (PDBSUM) (Profunc) (GPSS) (MMDB)	Target IDP01357	Resolution: 2Å	Title: The crystal structure of the peptide deformylase from Vibrio cholerae O1 biovar El Tor str. N16961
47	PDB 3FTT (PDBSUM) (Profunc) (GPSS) (MMDB)	Target IDP00698	Resolution: 1.6Å	Title: Crystal structure of the galactoside O-acetyltransferase from Staphylococcus aureus
48	PDB 3HYL (PDBSUM) (Profunc) (GPSS) (MMDB)	Target IDP02454	Resolution: 2.16Å	Title: Crystal structure of Transketolase from Bacillus anthracis
49	PDB 3HYK (PDBSUM) (Profunc) (GPSS) (MMDB)	Target IDP01182	Resolution: 2.31Å	Title: 2.31 Angstrom resolution crystal structure of a holo-(acyl-carrier-protein) synthase from Bacillus anthracis str. Ames in complex with CoA (3',5'-ADP)
50	PDB 3HVU (PDBSUM) (Profunc) (GPSS) (MMDB)	Target IDP01892	Resolution: 1.95Å	Title: 1.95 Angstrom Crystal Structure of Complex of Hypoxantine-GuaninePhosphoribosyltransferase from Bacillus anthracis with2-(N-morpholino)ethanesulfonic acid (MES)
51	PDB 3HMQ (PDBSUM) (Profunc) (GPSS) (MMDB)	Target IDP00960	Resolution: 1.9Å	Title: 1.9 Angstrom resolution crystal structure of a NAD synthetase (nadE) from Salmonella typhimurium LT2 in complex with NAD(+)
52	PDB 3HL3 (PDBSUM) (Profunc) (GPSS) (MMDB)	Target IDP01254	Resolution: 2.76Å	Title: 2.76 Angstrom Crystal Structure of a Putative Glucose-1-Phosphate Thymidylyltransferase from Bacillus anthracis in Complex with a Sucrose.
53	PDB 3HJV (PDBSUM) (Profunc) (GPSS) (MMDB)	Target IDP01440	Resolution: 1.7Å	Title: 1.7 Angstrom resolution crystal structure of an acyl carrier proteinS-malonyltransferase from Vibrio cholerae O1 biovar eltor str. N16961
54	PDB 3HJJ (PDBSUM) (Profunc) (GPSS) (MMDB)	Target IDP02453	Resolution: 2.15Å	Title: Crystal Structure of Maltose O-acetyltransferase from Bacillus anthracis
55	PDB 3HJB (PDBSUM) (Profunc) (GPSS) (MMDB)	Target IDP01329	Resolution: 1.5Å	Title: 1.5 Angstrom Crystal Structure of Glucose-6-phosphate Isomerase from Vibrio cholerae.
56	PDB 3HID (PDBSUM) (Profunc) (GPSS) (MMDB)	Target IDP00422	Resolution: 1.6Å	Title: Crystal structure of the adenylosuccinate synthetase from Yersinia pestis CO92
57	PDB 3HFR (PDBSUM) (Profunc) (GPSS) (MMDB)	Target IDP00347	Resolution: 2.3Å	Title: Crystal structure of glutamate racemase from Listeria monocytogenes
58	PDB 3H83 (PDBSUM) (Profunc) (GPSS) (MMDB)	Target IDP01892	Resolution: 2.06Å	Title: 2.06 Angstrom resolution structure of a hypoxanthine-guanine phosphoribosyltransferase (hpt-1) from Bacillus anthracis str. 'Ames Ancestor'
59	PDB 3H5Q (PDBSUM) (Profunc) (GPSS) (MMDB)	Target IDP00692	Resolution: 1.94Å	Title: Crystal structure of a putative pyrimidine-nucleoside phosphorylase from Staphylococcus aureus
60	PDB 3H2Y (PDBSUM) (Profunc) (GPSS) (MMDB)	Target IDP90222	Resolution: 1.8Å	Title: Crystal Structure of YqeH GTPase from Bacillus anthracis with dGDP Bound
61	PDB 3H1S (PDBSUM) (Profunc) (GPSS) (MMDB)	Target IDP01808	Resolution: 1.9Å	Title: Crystal structure of superoxide dismutase from Francisella tularensis subsp. tularensis SCHU S4
62	PDB 3H0P (PDBSUM) (Profunc) (GPSS) (MMDB)	Target IDP00956	Resolution: 2Å	Title: 2.0 Angstrom Crystal Structure of an Acyl Carrier Protein S-malonyltransferase from Salmonella typhimurium.
63	PDB 3H02 (PDBSUM) (Profunc) (GPSS) (MMDB)	Target IDP00995	Resolution: 2.15Å	Title: 2.15 Angstrom Resolution Crystal Structure of Naphthoate Synthase from Salmonella typhimurium.
64	PDB 3H07 (PDBSUM) (Profunc) (GPSS) (MMDB)	Target IDP00479	Resolution: 1.95Å	Title: Crystal structure of 3,4-dihydroxy-2-butanone 4-phosphate synthase from Yersinia pestis CO92
65	PDB 3GVD (PDBSUM) (Profunc) (GPSS) (MMDB)	Target IDP00538	Resolution: 2.4Å	Title: Crystal Structure of Serine Acetyltransferase CysE from Yersinia pestis
66	PDB 3GSD (PDBSUM) (Profunc) (GPSS) (MMDB)	Target IDP00456	Resolution: 2.05Å	Title: 2.05 Angstrom structure of a divalent-cation tolerance protein (CutA) from Yersinia pestis
67	PDB 3GSE (PDBSUM) (Profunc) (GPSS) (MMDB)	Target IDP00582	Resolution: 2.28Å	Title: Crystal structure of menaquinone-specific isochorismate synthase from Yersinia pestis CO92
68	PDB 3IHS (PDBSUM) (Profunc) (GPSS) (MMDB)	Target IDP01131	Resolution: 1.15Å	Title: CRYSTAL STRUCTURE OF A PHOSPHOCARRIER PROTEIN HPR FROM BACILLUS ANTHRACIS STR. AMES
69	PDB 3IGX (PDBSUM) (Profunc) (GPSS) (MMDB)	Target IDP02095	Resolution: 1.85Å	Title: 1.85 Angstrom Resolution Crystal Structure of Transaldolase B (talA) from Francisella tularensis.
70	PDB 3IGS (PDBSUM) (Profunc) (GPSS) (MMDB)	Target IDP01861	Resolution: 1.5Å	Title: Structure of the Salmonella enterica N-acetylmannosamine-6-phosphate 2-epimerase
71	PDB 3IG4 (PDBSUM) (Profunc) (GPSS) (MMDB)	Target IDP01115	Resolution: 2.9Å	Title: Structure of a putative aminopeptidase P from Bacillus anthracis
72	PDB 3IGJ (PDBSUM) (Profunc) (GPSS) (MMDB)	Target IDP02453	Resolution: 2.6Å	Title: Crystal Structure of Maltose O-Acetyl transferase Complexed with Acetyl Coenzyme A from Bacillus anthracis
73	PDB 3IFS (PDBSUM) (Profunc) (GPSS) (MMDB)	Target IDP01650	Resolution: 2Å	Title: 2.0 Angstrom Resolution Crystal Structure of Glucose-6-phosphate Isomerase (pgi) from Bacillus anthracis.
74	PDB 3IFE (PDBSUM) (Profunc) (GPSS) (MMDB)	Target IDP02712	Resolution: 1.55Å	Title: 1.55 Angstrom Resolution Crystal Structure of Peptidase T (pepT-1) from Bacillus anthracis str. 'Ames Ancestor'.
75	PDB 3IEB (PDBSUM) (Profunc) (GPSS) (MMDB)	Target IDP01486	Resolution: 2.1Å	Title: Crystal structure of 3-keto-L-gulonate-6-phosphate decarboxylase from Vibrio cholerae O1 biovar El Tor str. N16961
76	PDB 3ICC (PDBSUM) (Profunc) (GPSS) (MMDB)	Target IDP02573	Resolution: 1.87Å	Title: Crystal structure of a putative 3-oxoacyl-(acyl carrier protein) reductase from Bacillus anthracis at 1.87 A resolution
77	PDB 3IB3 (PDBSUM) (Profunc) (GPSS) (MMDB)	Target IDP00832	Resolution: 2.05Å	Title: Crystal Structure of SACOL2612 - CocE/NonD family hydrolase from Staphylococcus aureus
78	PDB 3IAH (PDBSUM) (Profunc) (GPSS) (MMDB)	Target IDP00971	Resolution: 1.83Å	Title: Crystal Structure of Short Chain Dehydrogenase (YCIK) from Salmonella enterica subsp. Enterica serovar Typhimurium str. LT2 in Complex with NADP and Acetate.
79	PDB 3IAC (PDBSUM) (Profunc) (GPSS) (MMDB)	Target IDP02065	Resolution: 2.22Å	Title: 2.2 Angstrom Crystal Structure of Glucuronate Isomerase from Salmonella typhimurium.
80	PDB 3I99 (PDBSUM) (Profunc) (GPSS) (MMDB)	Target IDP01372	Resolution: 2.2Å	Title: The crystal structure of the UDP-N-acetylenolpyruvoylglucosamine reductase from the Vibrio cholerae O1 biovar E1 Tor
81	PDB 3I3W (PDBSUM) (Profunc) (GPSS) (MMDB)	Target IDP02164	Resolution: 2.3Å	Title: STRUCTURE OF A PHOSPHOGLUCOSAMINE MUTASE FROM FRANCISELLA TULARENSIS
82	PDB 3I3O (PDBSUM) (Profunc) (GPSS) (MMDB)	Target IDP02499	Resolution: 2.06Å	Title: 2.06 Angstrom resolution crystal structure of a short chain dehydrogenase from Bacillus anthracis str. 'Ames Ancestor' in complex with NAD-acetone
83	PDB 3I1I (PDBSUM) (Profunc) (GPSS) (MMDB)	Target IDP01610	Resolution: 2.44Å	Title: X-ray crystal structure of homoserine O-acetyltransferase from Bacillus anthracis.
84	PDB 3I12 (PDBSUM) (Profunc) (GPSS) (MMDB)	Target IDP00919	Resolution: 2.2Å	Title: The crystal structure of the D-alanyl-alanine synthetase A from Salmonella enterica subsp. enterica serovar Typhimurium str. LT2
85	PDB 3I07 (PDBSUM) (Profunc) (GPSS) (MMDB)	Target IDP01325	Resolution: 1.5Å	Title: CRYSTAL STRUCTURE OF A PUTATIVE ORGANIC HYDROPEROXIDE RESISTANCE PROTEIN FROM VIBRIO CHOLERAE O1BIOVAR ELTOR STR. N16961
86	PDB 3HZN (PDBSUM) (Profunc) (GPSS) (MMDB)	Target IDP00923	Resolution: 2.4Å	Title: Structure of the Salmonella typhimurium nfnB dihydropteridine reductase
87	PDB 3HYQ (PDBSUM) (Profunc) (GPSS) (MMDB)	Target IDP01970	Resolution: 1.53Å	Title: Crystal Structure of Isopentenyl-Diphosphate delta-Isomerase from Salmonella entericase
88	PDB 3K1S (PDBSUM) (Profunc) (GPSS) (MMDB)	Target IDP01100	Resolution: 2.3Å	Title: Crystal Structure of the PTS Cellobiose Specific Enzyme IIA from Bacillus anthracis
89	PDB 3JZE (PDBSUM) (Profunc) (GPSS) (MMDB)	Target IDP00873	Resolution: 1.8Å	Title: 1.8 Angstrom Resolution Crystal Structure of Dihydroorotase (pyrc) from Salmonella enterica subsp. enterica serovar Typhimurium str. LT2.
90	PDB 3JTJ (PDBSUM) (Profunc) (GPSS) (MMDB)	Target IDP02355	Resolution: 2.18Å	Title: 3-deoxy-manno-octulosonate cytidylyltransferase from Yersinia pestis.
91	PDB 3JR2 (PDBSUM) (Profunc) (GPSS) (MMDB)	Target IDP01486	Resolution: 1.8Å	Title: X-ray crystal structure of the Mg-bound 3-keto-L-gulonate-6-phosphatedecarboxylase from Vibrio cholerae O1 biovar El Tor str. N16961
92	PDB 3IWH (PDBSUM) (Profunc) (GPSS) (MMDB)	Target IDP00640	Resolution: 2Å	Title: Crystal Structure of Rhodanese-like Domain Protein from Staphylococcus aureus
93	PDB 3IV8 (PDBSUM) (Profunc) (GPSS) (MMDB)	Target IDP01334	Resolution: 2.53Å	Title: N-acetylglucosamine-6-phosphate deacetylase from Vibrio cholerae complexed with fructose 6-phosphate.
94	PDB 3IST (PDBSUM) (Profunc) (GPSS) (MMDB)	Target IDP00347	Resolution: 1.65Å	Title: Crystal structure of glutamate racemase from Listeria monocytogenes in complex with succinic acid
95	PDB 3ISV (PDBSUM) (Profunc) (GPSS) (MMDB)	Released: 2009-09-22	Resolution: 1.85Å	Title: Crystal structure of glutamate racemase from Listeria monocytogenes in complex with acetate ion
96	PDB 3IR4 (PDBSUM) (Profunc) (GPSS) (MMDB)	Target IDP00895	Resolution: 1.2Å	Title: 1.2 Angstrom Crystal Structure of the Glutaredoxin 2 (grxB) from Salmonella typhimurium in complex with Glutathione.
97	PDB 3IRC (PDBSUM) (Profunc) (GPSS) (MMDB)	Target IDP00272	Resolution: 2.2Å	Title: Crystal structure analysis of dengue-1 envelope protein domain III
98	PDB 3IQ1 (PDBSUM) (Profunc) (GPSS) (MMDB)	Target IDP01318	Resolution: 1.67Å	Title: CRYSTAL STRUCTURE OF DPS PROTEIN FROM VIBRIO CHOLERAE O1, A MEMBER OF A BROAD SUPERFAMILY OF FERRITIN-LIKE DIIRON-CARBOXYLATE PROTEINS
99	PDB 3INP (PDBSUM) (Profunc) (GPSS) (MMDB)	Target IDP02542	Resolution: 2.05Å	Title: 2.05 Angstrom Resolution Crystal Structure of D-ribulose-phosphate 3-epimerase from Francisella tularensis.
100	PDB 3IMF (PDBSUM) (Profunc) (GPSS) (MMDB)	Target IDP02325	Resolution: 1.99Å	Title: 1.99 Angstrom resolution crystal structure of a short chain dehydrogenase from Bacillus anthracis str. 'Ames Ancestor'

101	PDB 3IMI (PDBSUM) (Profunc) (GPSS) (MMDB)	Target IDP02451	Resolution: 2.01Å	Title: 2.01 Angstrom resolution crystal structure of a HIT family protein from Bacillus anthracis str. 'Ames Ancestor'
102	PDB 3IJW (PDBSUM) (Profunc) (GPSS) (MMDB)	Target IDP00044	Resolution: 1.9Å	Title: Crystal structure of BA2930 in complex with CoA
103	PDB 3IJR (PDBSUM) (Profunc) (GPSS) (MMDB)	Target IDP02499	Resolution: 2.05Å	Title: 2.05 Angstrom resolution crystal structure of a short chain dehydrogenase from Bacillus anthracis str. 'Ames Ancestor' in complex with NAD+
104	PDB 3IJ3 (PDBSUM) (Profunc) (GPSS) (MMDB)	Target IDP01962	Resolution: 1.8Å	Title: 1.8 Angstrom Resolution Crystal Structure of Cytosol Aminopeptidase from Coxiella burnetii
105	PDB 3IJ5 (PDBSUM) (Profunc) (GPSS) (MMDB)	Target IDP02274	Resolution: 1.95Å	Title: 1.95 Angstrom Resolution Crystal Structure of 3-deoxy-D-manno-octulosonate 8-phosphate Phosphatase from Yersinia pestis.
106	PDB 3III (PDBSUM) (Profunc) (GPSS) (MMDB)	Target IDP00832	Resolution: 1.95Å	Title: 1.95 Angstrom Crystal Structure of CocE/NonD family hydrolase (SACOL2612) from Staphylococcus aureus
107	PDB 3IIE (PDBSUM) (Profunc) (GPSS) (MMDB)	Target IDP00499	Resolution: 2.21Å	Title: 1-deoxy-D-xylulose 5-phosphate reductoisomerase from Yersinia pestis.
108	PDB 3LG3 (PDBSUM) (Profunc) (GPSS) (MMDB)	Target IDP02638	Resolution: 1.4Å	Title: 1.4A CRYSTAL STRUCTURE OF ISOCITRATE LYASE FROM YERSINIA PESTIS CO92
109	PDB 3LAY (PDBSUM) (Profunc) (GPSS) (MMDB)	Target IDP01826	Resolution: 2.7Å	Title: alpha-Helical barrel formed by the decamer of the zinc resistance-associated protein (STM4172) from Salmonella enterica subsp. enterica serovar Typhimurium str. LT2
110	PDB 3LAC (PDBSUM) (Profunc) (GPSS) (MMDB)	Target IDP02531	Resolution: 2Å	Title: Crystal structure of Bacillus anthracis pyrrolidone-carboxylate peptidase, pcP
111	PDB 3L4E (PDBSUM) (Profunc) (GPSS) (MMDB)	Target IDP02527	Resolution: 1.5Å	Title: 1.5A Crystal Structure of a hypothetical protein lmo0363 from Listeria monocytogenes EGD-e
112	PDB 3L44 (PDBSUM) (Profunc) (GPSS) (MMDB)	Target IDP02280	Resolution: 2.05Å	Title: Crystal structure of Bacillus anthracis HemL-1, glutamate semialdehyde aminotransferase
113	PDB 3L07 (PDBSUM) (Profunc) (GPSS) (MMDB)	Target IDP01849	Resolution: 1.88Å	Title: Methylenetetrahydrofolate dehydrogenase/methenyltetrahydrofolate cyclohydrolase, putative bifunctional protein from Francisella tularensis.
114	PDB 3KZW (PDBSUM) (Profunc) (GPSS) (MMDB)	Target IDP00662	Resolution: 2.7Å	Title: Crystal structure of cytosol aminopeptidase from Staphylococcus aureus COL
115	PDB 3KZL (PDBSUM) (Profunc) (GPSS) (MMDB)	Target IDP00044	Resolution: 2.1Å	Title: Crystal structure of BA2930 mutant (H183G) in complex with AcCoA
116	PDB 3KWO (PDBSUM) (Profunc) (GPSS) (MMDB)	Target IDP01964	Resolution: 1.99Å	Title: X-ray crystal structure of Putative Bacterioferritin from Campylobacter jejuni
117	PDB 3KWM (PDBSUM) (Profunc) (GPSS) (MMDB)	Target IDP02119	Resolution: 2.32Å	Title: Crystal structure of ribose-5-isomerase A
118	PDB 3KUX (PDBSUM) (Profunc) (GPSS) (MMDB)	Target IDP02458	Resolution: 2.75Å	Title: Structure of the YPO2259 putative oxidoreductase from Yersinia pestis
119	PDB 3KUU (PDBSUM) (Profunc) (GPSS) (MMDB)	Target IDP02536	Resolution: 1.4Å	Title: Structure of the PurE Phosphoribosylaminoimidazole Carboxylase Catalytic Subunit from Yersinia pestis
120	PDB 3KQF (PDBSUM) (Profunc) (GPSS) (MMDB)	Target IDP02329	Resolution: 1.8Å	Title: 1.8 Angstrom Resolution Crystal Structure of Enoyl-CoA Hydratase from Bacillus anthracis.
121	PDB 3KOM (PDBSUM) (Profunc) (GPSS) (MMDB)	Target IDP02310	Resolution: 1.6Å	Title: Crystal structure of apo Transketolase from Francisella tularensis
122	PDB 3KHY (PDBSUM) (Profunc) (GPSS) (MMDB)	Target IDP01739	Resolution: 1.98Å	Title: Crystal Structure of a Propionate Kinase from Francisella tularensis subsp. tularensis SCHU S4
123	PDB 3KD3 (PDBSUM) (Profunc) (GPSS) (MMDB)	Target IDP02448	Resolution: 1.7Å	Title: CRYSTAL STRUCTURE OF A PHOSPHOSERINE PHOSPHOHYDROLASE-LIKE PROTEIN FROM FRANCISELLA TULARENSIS SUBSP. TULARENSIS SCHUS4
124	PDB 3KBO (PDBSUM) (Profunc) (GPSS) (MMDB)	Target IDP00951	Resolution: 2.14Å	Title: 2.14 Angstrom Crystal Structure of Putative Oxidoreductase (ycdW) from Salmonella typhimurium in Complex with NADP.
125	PDB 3KB8 (PDBSUM) (Profunc) (GPSS) (MMDB)	Target IDP01892	Resolution: 2.09Å	Title: 2.09 Angstrom resolution structure of a hypoxanthine-guanine phosphoribosyltransferase (hpt-1) from Bacillus anthracis str. 'Ames Ancestor' in complex with GMP
126	PDB 3K96 (PDBSUM) (Profunc) (GPSS) (MMDB)	Target IDP01976	Resolution: 2.1Å	Title: 2.1 Angstrom resolution crystal structure of glycerol-3-phosphate dehydrogenase (gpsA) from Coxiella burnetii.
127	PDB 3K28 (PDBSUM) (Profunc) (GPSS) (MMDB)	Target IDP02781	Resolution: 1.95Å	Title: CRYSTAL STRUCTURE OF A GLUTAMATE-1-SEMIALDEHYDE AMINOTRANSFERASE FROM BACILLUS ANTHRACIS WITH BOUND PYRIDOXAL 5'PHOSPHATE
128	PDB 3N0S (PDBSUM) (Profunc) (GPSS) (MMDB)	Target IDP00044	Resolution: 2.15Å	Title: Crystal structure of BA2930 mutant (H183A) in complex with AcCoA
129	PDB 3MZZ (PDBSUM) (Profunc) (GPSS) (MMDB)	Target IDP00640	Resolution: 2Å	Title: Crystal Structure of Rhodanese-like Domain Protein from Staphylococcus aureus
130	PDB 3MSZ (PDBSUM) (Profunc) (GPSS) (MMDB)	Target IDP01801	Resolution: 2.05Å	Title: Crystal Structure of Glutaredoxin 1 from Francisella tularensis Complexed with Cacodylate
131	PDB 3MSU (PDBSUM) (Profunc) (GPSS) (MMDB)	Target IDP04634	Resolution: 1.84Å	Title: Crystal Structure of Citrate Synthase from Francisella tularensis
132	PDB 3MJD (PDBSUM) (Profunc) (GPSS) (MMDB)	Target IDP02311	Resolution: 1.9Å	Title: 1.9 Angstrom Crystal Structure of Orotate Phosphoribosyltransferase (pyrE) Francisella tularensis.
133	PDB 3MJF (PDBSUM) (Profunc) (GPSS) (MMDB)	Target IDP04490	Resolution: 1.47Å	Title: Phosphoribosylamine-glycine ligase from Yersinia pestis.
134	PDB 3MGA (PDBSUM) (Profunc) (GPSS) (MMDB)	Target IDP00118	Resolution: 2.4Å	Title: 2.4 Angstrom Crystal Structure of Ferric Enterobactin Esterase (fes) from Salmonella typhimurium.
135	PDB 3M84 (PDBSUM) (Profunc) (GPSS) (MMDB)	Target IDP04643	Resolution: 1.7Å	Title: Crystal Structure of Phosphoribosylaminoimidazole Synthetase from Francisella tularensis
136	PDB 3M5P (PDBSUM) (Profunc) (GPSS) (MMDB)	Target IDP02733	Resolution: 1.65Å	Title: Glucose-6-phosphate isomerase from Francisella tularensis complexed with fructose-6-phosphate.
137	PDB 3M49 (PDBSUM) (Profunc) (GPSS) (MMDB)	Target IDP02454	Resolution: 2Å	Title: Crystal Structure of Transketolase Complexed with Thiamine Diphosphate from Bacillus anthracis
138	PDB 3M3H (PDBSUM) (Profunc) (GPSS) (MMDB)	Target IDP04423	Resolution: 1.75Å	Title: 1.75 Angstrom resolution crystal structure of an orotate phosphoribosyltransferase from Bacillus anthracis str. 'Ames Ancestor'
139	PDB 3M07 (PDBSUM) (Profunc) (GPSS) (MMDB)	Target IDP00968	Resolution: 1.4Å	Title: 1.4 Angstrom Resolution Crystal Structure of Putative alpha Amylase from Salmonella typhimurium.
140	PDB 3LYL (PDBSUM) (Profunc) (GPSS) (MMDB)	Target IDP00334	Resolution: 1.95Å	Title: Structure of 3-oxoacyl-acylcarrier protein reductase, FabG from Francisella tularensis
141	PDB 3LUS (PDBSUM) (Profunc) (GPSS) (MMDB)	Target IDP01325	Resolution: 1.96Å	Title: Crystal structure of a putative organic hydroperoxide resistance protein with molecule of captopril bound in one of the active sites from Vibrio cholerae
142	PDB 3LU2 (PDBSUM) (Profunc) (GPSS) (MMDB)	Target IDP02710	Resolution: 2.2Å	Title: Structure of lmo2462, a Listeria monocytogenes amidohydrolase family putative dipeptidase
143	PDB 3LQN (PDBSUM) (Profunc) (GPSS) (MMDB)	Target IDP02609	Resolution: 1.8Å	Title: Crystal Structure of CBS Domain-containing Protein of Unknown Function from Bacillus anthracis str. 'Ames Ancestor'
144	PDB 3LNO (PDBSUM) (Profunc) (GPSS) (MMDB)	Target IDP02795	Resolution: 2.1Å	Title: Crystal Structure of Domain of Unknown Function DUF59 from Bacillus anthracis
145	PDB 3LJK (PDBSUM) (Profunc) (GPSS) (MMDB)	Target IDP02733	Resolution: 1.48Å	Title: Glucose-6-phosphate isomerase from Francisella tularensis.
146	PDB 3LHQ (PDBSUM) (Profunc) (GPSS) (MMDB)	Target IDP02616	Resolution: 1.56Å	Title: DNA-binding transcriptional repressor AcrR from Salmonella typhimurium.
147	PDB 3LGC (PDBSUM) (Profunc) (GPSS) (MMDB)	Target IDP01801	Resolution: 2.77Å	Title: Crystal Structure of Glutaredoxin 1 from Francisella tularensis
148	PDB 3OO2 (PDBSUM) (Profunc) (GPSS) (MMDB)	Target IDP00688	Resolution: 2.37Å	Title: 2.37 Angstrom resolution crystal structure of an alanine racemase (alr) from Staphylococcus aureus subsp. aureus COL
149	PDB 3OKF (PDBSUM) (Profunc) (GPSS) (MMDB)	Target IDP04176	Resolution: 2.5Å	Title: 2.5 Angstrom Resolution Crystal Structure of 3-Dehydroquinate Synthase (aroB) from Vibrio cholerae
150	PDB 3OJC (PDBSUM) (Profunc) (GPSS) (MMDB)	Target IDP04122	Resolution: 1.75Å	Title: Crystal structure of a putative Asp/Glu Racemase from Yersinia pestis
151	PDB 3OGA (PDBSUM) (Profunc) (GPSS) (MMDB)	Target IDP01880	Resolution: 1.75Å	Title: 1.75 Angstrom resolution crystal structure of a putative NTP pyrophosphohydrolase (yfaO) from Salmonella typhimurium LT2
152	PDB 3OF5 (PDBSUM) (Profunc) (GPSS) (MMDB)	Target IDP02289	Resolution: 1.52Å	Title: Crystal Structure of a Dethiobiotin Synthetase from Francisella tularensis subsp. tularensis SCHU S4
153	PDB 3O7M (PDBSUM) (Profunc) (GPSS) (MMDB)	Target IDP01634	Resolution: 1.98Å	Title: 1.98 Angstrom resolution crystal structure of a hypoxanthine-guanine phosphoribosyltransferase (hpt-2) from Bacillus anthracis str. 'Ames Ancestor'
154	PDB 3O6V (PDBSUM) (Profunc) (GPSS) (MMDB)	Target IDP04345	Resolution: 1.7Å	Title: Crystal structure of Uridine Phosphorylase from Vibrio cholerae O1 biovar El Tor
155	PDB 3O1K (PDBSUM) (Profunc) (GPSS) (MMDB)	Target IDP01380	Resolution: 1.95Å	Title: CRYSTAL STRUCTURE OF PUTATIVE DIHYDRONEOPTERIN ALDOLASE (FOLB) FROM VIBRIO CHOLERAE O1 BIOVAR EL TOR STR N16961
156	PDB 3O04 (PDBSUM) (Profunc) (GPSS) (MMDB)	Target IDP05301	Resolution: 1.85Å	Title: Crystal structure of the beta-keto-acyl carrier protein synthase II (lmo2201) from Listeria monocytogenes
157	PDB 3NZT (PDBSUM) (Profunc) (GPSS) (MMDB)	Target IDP01828	Resolution: 2Å	Title: 2.0 Angstrom Crystal structure of Glutamate-Cysteine Ligase (gshA) ftom Francisella tularensis in Comlex with AMP.
158	PDB 3NZ2 (PDBSUM) (Profunc) (GPSS) (MMDB)	Target IDP01515	Resolution: 2.35Å	Title: Crystal Structure of Hexapeptide-Repeat Containing-Acetyltransferase, VCA0836 from Vibrio cholerae O1 biovar eltor
159	PDB 3NX4 (PDBSUM) (Profunc) (GPSS) (MMDB)	Target IDP00076	Resolution: 1.9Å	Title: Crystal structure of the yhdH oxidoreductase from Salmonella enterica in complex with NADP
160	PDB 3NVT (PDBSUM) (Profunc) (GPSS) (MMDB)	Target IDP00348	Resolution: 1.95Å	Title: 1.95 Angstrom crystal structure of a bifunctional 3-deoxy-7-phosphoheptulonate synthase/chorismate mutase (aroA) from Listeria monocytogenes EGD-e
161	PDB 3NUA (PDBSUM) (Profunc) (GPSS) (MMDB)	Target IDP04560	Resolution: 1.4Å	Title: Crystal Structure of Phosphoribosylaminoimidazole-Succinocarboxamide Synthase from Clostridium perfringens
162	PDB 3NTX (PDBSUM) (Profunc) (GPSS) (MMDB)	Target IDP04466	Resolution: 1.9Å	Title: Crystal Structure of L-asparaginase I from Yersinia pestis
163	PDB 3NAV (PDBSUM) (Profunc) (GPSS) (MMDB)	Target IDP04160	Resolution: 2.1Å	Title: CRYSTAL STRUCTURE OF AN ALPHA SUBUNIT OF TRYPTOPHAN SYNTHASE FROM VIBRIO CHOLERAE O1 BIOVAR EL TOR STR N16961
164	PDB 3N8H (PDBSUM) (Profunc) (GPSS) (MMDB)	Target IDP02771	Resolution: 2Å	Title: Crystal Structure of Pantoate-beta-alanine Ligase Comlexed with AMP fromFrancisella tularensis
165	PDB 3N77 (PDBSUM) (Profunc) (GPSS) (MMDB)	Target IDP01880	Resolution: 1.86Å	Title: CRYSTAL STRUCTURE OF IDP01880, PUTATIVE NTP PYROPHOSPHOHYDROLASE OFSALMONELLA TYPHIMURIUM LT2
166	PDB 3N5M (PDBSUM) (Profunc) (GPSS) (MMDB)	Target IDP01705	Resolution: 2.05Å	Title: Crystals structure of a Bacillus anthracis aminotransferase
167	PDB 3N0M (PDBSUM) (Profunc) (GPSS) (MMDB)	Target IDP00044	Resolution: 2.4Å	Title: Crystal structure of BA2930 mutant (H183G) in complex with AcCoA
168	PDB 3P7M (PDBSUM) (Profunc) (GPSS) (MMDB)	Target IDP02372	Resolution: 2.2Å	Title: Structure of putative lactate dehydrogenase from Francisella tularensis subsp. tularensis SCHU S4
169	PDB 3N2A (PDBSUM) (Profunc) (GPSS) (MMDB)	Target IDP90784	Resolution: 1.9Å	Title: CRYSTAL STRUCTURE OF BIFUNCTIONAL FOLYLPOLYGLUTAMATE SYNTHASE/DIHYDROFOLATE SYNTHASE COMPLEXED WITH ADP FROM YERSINIA PESTIS C092
170	PDB 3P54 (PDBSUM) (Profunc) (GPSS) (MMDB)	Target IDP00254	Resolution: 2.1Å	Title: Crystal Structure of the Japanese Encephalitis Virus Envelope Protein, strain SA-14-14-2.
171	PDB 3P2L (PDBSUM) (Profunc) (GPSS) (MMDB)	Target IDP02821	Resolution: 2.3Å	Title: Crystal Structure of ATP-dependent Clp Protease Subunit P from Francisella tularensis
172	PDB 3P2A (PDBSUM) (Profunc) (GPSS) (MMDB)	Target IDP04482	Resolution: 2.2Å	Title: Crystal Structure of Thioredoxin 2 from Yersinia pestis
173	PDB 3P0R (PDBSUM) (Profunc) (GPSS) (MMDB)	Target IDP04035	Resolution: 1.8Å	Title: Crystal structure of azoreductase from Bacillus anthracis str. Sterne
174	PDB 3P09 (PDBSUM) (Profunc) (GPSS) (MMDB)	Target IDP02545	Resolution: 1.9Å	Title: Crystal Structure of Beta-Lactamase from Francisella tularensis
175	PDB 3OZH (PDBSUM) (Profunc) (GPSS) (MMDB)	Target IDP00523	Resolution: 1.91Å	Title: Crystal Structure of Beta-Lactamase/D-Alanine Carboxypeptidase from Yersinia pestis
176	PDB 3OYT (PDBSUM) (Profunc) (GPSS) (MMDB)	Target IDP00577	Resolution: 1.84Å	Title: 1.84 Angstrom resolution crystal structure of 3-oxoacyl-(acyl carrier protein) synthase I (fabB) from Yersinia pestis CO92
177	PDB 3OXP (PDBSUM) (Profunc) (GPSS) (MMDB)	Target IDP04293	Resolution: 1.2Å	Title: Structure of phosphotransferase enzyme II, A component from Yersinia pestis CO92 at 1.2 A resolution
178	PDB 3OWA (PDBSUM) (Profunc) (GPSS) (MMDB)	Target IDP04432	Resolution: 1.97Å	Title: Crystal Structure of Acyl-CoA Dehydrogenase complexed with FAD from Bacillus anthracis
179	PDB 3OUT (PDBSUM) (Profunc) (GPSS) (MMDB)	Target IDP00335	Resolution: 1.65Å	Title: Crystal structure of glutamate racemase from Francisella tularensis subsp. tularensis SCHU S4 in complex with D-glutamate.
180	PDB 3OUG (PDBSUM) (Profunc) (GPSS) (MMDB)	Target IDP02730	Resolution: 1.55Å	Title: Crystal structure of cleaved L-aspartate-alpha-decarboxylase fromFrancisella tularensis
181	PDB 3OT5 (PDBSUM) (Profunc) (GPSS) (MMDB)	Target IDP01656	Resolution: 2.2Å	Title: 2.2 Angstrom Resolution Crystal Structure of putative UDP-N-acetylglucosamine 2-epimerase from Listeria monocytogenes
182	PDB 3OT1 (PDBSUM) (Profunc) (GPSS) (MMDB)	Target IDP04323	Resolution: 1.16Å	Title: Crystal structure of VC2308 protein
183	PDB 3OSU (PDBSUM) (Profunc) (GPSS) (MMDB)	Target IDP05774	Resolution: 1.9Å	Title: Crystal structure of the 3-oxoacyl-acyl carrier protein reductase, FabG, from Staphylococcus aureus
184	PDB 3OS6 (PDBSUM) (Profunc) (GPSS) (MMDB)	Target IDP01205	Resolution: 2.4Å	Title: Crystal structure of putative 2,3-dihydroxybenzoate-specific isochorismate synthase, DhbC from Bacillus anthracis.
185	PDB 3OQ3 (PDBSUM) (Profunc) (GPSS) (MMDB)	Target IDP05773	Resolution: 2.1Å	Title: Structural Basis of Type-I Interferon Sequestration by a Poxvirus Decoy Receptor
186	PDB 3OPK (PDBSUM) (Profunc) (GPSS) (MMDB)	Target IDP01083	Resolution: 1.9Å	Title: Crystal structure of divalent-cation tolerance protein CutA from Salmonella enterica
187	PDB 3OPQ (PDBSUM) (Profunc) (GPSS) (MMDB)	Target IDP04072	Resolution: 2Å	Title: Phosphoribosylaminoimidazole carboxylase with fructose-6-phosphate bound to the central channel of the octameric protein structure.
188	PDB 3OOW (PDBSUM) (Profunc) (GPSS) (MMDB)	Released: 2010-11-17	Resolution: 1.75Å	Title: Octameric structure of the phosphoribosylaminoimidazole carboxylasecatalytic subunit from Francisella tularensis subsp. tularensis SCHU S4.
189	PDB 3QM2 (PDBSUM) (Profunc) (GPSS) (MMDB)	Target IDP00945	Resolution: 2.25Å	Title: 2.25 Angstrom Crystal Structure of Phosphoserine Aminotransferase (SerC)from Salmonella enterica subsp. enterica serovar Typhimurium
190	PDB 3QJG (PDBSUM) (Profunc) (GPSS) (MMDB)	Target IDP00629	Resolution: 2.04Å	Title: Epidermin biosynthesis protein EpiD from Staphylococcus aureus.
191	PDB 3QFH (PDBSUM) (Profunc) (GPSS) (MMDB)	Target IDP00624	Resolution: 2.05Å	Title: 2.05 Angstrom Resolution Crystal Structure of Epidermin Leader Peptide Processing Serine Protease (EpiP) from Staphylococcus aureus.
192	PDB 3QDN (PDBSUM) (Profunc) (GPSS) (MMDB)	Target IDP01793	Resolution: 2.09Å	Title: Putative thioredoxin protein from Salmonella typhimurium.
193	PDB 3QAO (PDBSUM) (Profunc) (GPSS) (MMDB)	Target IDP02505	Resolution: 1.87Å	Title: THE CRYSTAL STRUCTURE OF THE N-TERMINAL DOMAIN OF A MERR-LIKE TRANSCRIPTIONAL REGULATOR FROM LISTERIA MONOCYTOGENES EGD-E
194	PDB 3Q94 (PDBSUM) (Profunc) (GPSS) (MMDB)	Target IDP01865	Resolution: 2.3Å	Title: THE CRYSTAL STRUCTURE OF FRUCTOSE 1,6-BISPHOSPHATE ALDOLASE FROM BACILLUS ANTHRACIS STR. 'AMES ANCESTOR'
195	PDB 3Q88 (PDBSUM) (Profunc) (GPSS) (MMDB)	Target IDP02733	Resolution: 1.7Å	Title: Glucose-6-phosphate isomerase from Francisella tularensis complexed with ribose 1,5-bisphosphate.
196	PDB 3Q7H (PDBSUM) (Profunc) (GPSS) (MMDB)	Target IDP02028	Resolution: 2.5Å	Title: Structure of the ClpP subunit of the ATP-dependent Clp Protease from Coxiella burnetii
197	PDB 3Q7I (PDBSUM) (Profunc) (GPSS) (MMDB)	Target IDP02733	Resolution: 1.54Å	Title: Glucose-6-phosphate isomerase from Francisella tularensis complexed with 6-phosphogluconic acid.
198	PDB 3Q6D (PDBSUM) (Profunc) (GPSS) (MMDB)	Target IDP01139	Resolution: 1.97Å	Title: Xaa-Pro dipeptidase from Bacillus anthracis.
199	PDB 3Q58 (PDBSUM) (Profunc) (GPSS) (MMDB)	Target IDP04524	Resolution: 1.8Å	Title: Structure of N-acetylmannosamine-6-Phosphate Epimerase from Salmonella enterica
200	PDB 3Q1K (PDBSUM) (Profunc) (GPSS) (MMDB)	Target IDP00919	Resolution: 2.2Å	Title: The Crystal Structure of the D-alanyl-alanine Synthetase A from Salmonella enterica Typhimurium Complexed with ADP

201	PDB 3PZS (PDBSUM) (Profunc) (GPSS) (MMDB)	Target IDP04384	Resolution: 1.89Å	Title: Crystal Structure of a pyridoxamine kinase from Yersinia pestis CO92
202	PDB 3PP8 (PDBSUM) (Profunc) (GPSS) (MMDB)	Target IDP00951	Resolution: 2.1Å	Title: 2.1 Angstrom Crystal Structure of Putative Oxidoreductase (ycdW) from Salmonella typhimurium.
203	PDB 3PP9 (PDBSUM) (Profunc) (GPSS) (MMDB)	Target IDP00047	Resolution: 1.6Å	Title: 1.6 Angstrom resolution crystal structure of putative streptothricin acetyltransferase from Bacillus anthracis str. Ames in complex with acetyl coenzyme A
204	PDB 3PNS (PDBSUM) (Profunc) (GPSS) (MMDB)	Target IDP04345	Resolution: 2Å	Title: Crystal Structure of Uridine Phosphorylase from Vibrio cholerae Complexed with Uracil
205	PDB 3PGY (PDBSUM) (Profunc) (GPSS) (MMDB)	Target IDP00749	Resolution: 1.92Å	Title: Serine hydroxymethyltransferase from Staphylococcus aureus, S95P mutant.
206	PDB 3PFI (PDBSUM) (Profunc) (GPSS) (MMDB)	Target IDP00067	Resolution: 2.69Å	Title: 2.7 Angstrom resolution crystal structure of a probable holliday junction DNA helicasae (ruvB) from Campylobacter jejuni subsp. jejuni NCTC 11168 in complex with adenosine-5'-diphospahte
207	PDB 3PEI (PDBSUM) (Profunc) (GPSS) (MMDB)	Target IDP02398	Resolution: 2.7Å	Title: Crystal Structure of Cytsol Aminopeptidase fromFrancisella tularensis
208	PDB 3PEA (PDBSUM) (Profunc) (GPSS) (MMDB)	Target IDP04689	Resolution: 1.82Å	Title: Crystal structure of enoyl-CoA hydratase from Bacillus anthracis str. 'Ames Ancestor'
209	PDB 3RJ4 (PDBSUM) (Profunc) (GPSS) (MMDB)	Target IDP01750	Resolution: 1.75Å	Title: Crystal Structure of 7-cyano-7-deazaguanine Reductase, QueF from Vibrio cholerae O1 biovar El Tor
210	PDB 3RJU (PDBSUM) (Profunc) (GPSS) (MMDB)	Target IDP00523	Resolution: 1.5Å	Title: Crystal Structure of Beta-lactamase/D-alanine Carboxypeptidase from Yersinia pestis complexed with citrate
211	PDB 3RHI (PDBSUM) (Profunc) (GPSS) (MMDB)	Target IDP04601	Resolution: 2.48Å	Title: DNA-binding protein HU from Bacillus anthracis.
212	PDB 3RF0 (PDBSUM) (Profunc) (GPSS) (MMDB)	Target IDP02550	Resolution: 1.8Å	Title: Crystal Structure of Exopolyphosphatase from Yersinia pestis
213	PDB 3RE3 (PDBSUM) (Profunc) (GPSS) (MMDB)	Target IDP00331	Resolution: 2.65Å	Title: Crystal Structure of 2-C-Methyl-D-Erythritol 2,4-Cyclodiphosphate Synthase from Francisella tularensis
214	PDB 3RDW (PDBSUM) (Profunc) (GPSS) (MMDB)	Target IDP00581	Resolution: 2.2Å	Title: Putative arsenate reductase from Yersinia pestis.
215	PDB 3ROI (PDBSUM) (Profunc) (GPSS) (MMDB)	Target IDP01842	Resolution: 2.2Å	Title: 2.20 Angstrom resolution structure of 3-phosphoshikimate 1-carboxyvinyltransferase (AroA) from Coxiella burnetii
216	PDB 3R8Y (PDBSUM) (Profunc) (GPSS) (MMDB)	Target IDP01143	Resolution: 1.7Å	Title: Structure of the Bacillus anthracis tetrahydropicolinate succinyltransferase
217	PDB 3R5X (PDBSUM) (Profunc) (GPSS) (MMDB)	Target IDP01207	Resolution: 2Å	Title: Crystal Structure of D-alanine--D-Alanine Ligase from Bacillus anthracis complexed with ATP
218	PDB 3R3S (PDBSUM) (Profunc) (GPSS) (MMDB)	Target IDP01048	Resolution: 1.25Å	Title: Structure of the YghA Oxidoreductase from Salmonella enterica
219	PDB 3R3R (PDBSUM) (Profunc) (GPSS) (MMDB)	Target IDP01056	Resolution: 1.2Å	Title: Structure of the YrdA ferripyochelin binding protein from Salmonella enterica
220	PDB 3R3T (PDBSUM) (Profunc) (GPSS) (MMDB)	Target IDP04069	Resolution: 2.3Å	Title: Crystal Structure of 30S Ribosomal Protein S from Bacillus anthracis
	*those in blue font denote structures selected for further investigation			

Figure S2. List of Protein Crystal Structures Examined. The first 220 crystal structures deposited into the Protein Database (PDB) from the Center for Structural Genomics of Infectious Diseases (CSGID) are listed.

Supplementary Table. Rank order binding of compounds to protein pockets

	Dxr1			Dxr2		
Rank	ZINC ID*	Smiles**	Score	ZINC ID	Smiles	Score
1	ZINC20136854	Cc1c(c2c(cn1)CN(CC2)c3ccnc(n3)C)c4nc(on4)C5CC5	-112.851	ZINC08275376	Cc1c(snn1)C(=O)OCCCn2c3ccccc3[nH]c2=O	-71.031
2	ZINC17071832	c1ccc-2c(c1)CCc3c2sc(c3C(=O)N)NC(=O)CCC(=O)[O-]	-104.029	ZINC07126183	COc1ccccc1C=CC2[NH+]=C(N=C3N2c4ccccc4N3)N	-70.369
3	ZINC08582280	c1cc(ccc1C(=O)[O-])NCCC2CNc3c(c(nc(n3)N)O)N2	-103.413	ZINC14125480	c1ccc2c(c1)[nH]c(=O)n2CCCOC(=O)c3ccoc3	-69.707
4	ZINC12561276	c1ccc2c(c1)[nH]c3n2c(nn3)SCc4ccc(o4)C(=O)[O-]	-103.201	ZINC12508018	CCn1c2ccc(cc2nc1CSc3nc([nH]n3)N)C(=O)[O-]	-69.576
5	ZINC26472880	c1ccc(cc1)c2nc([nH]n2)SCc3nc(nc(n3)N)N	-103.117	ZINC23144933	Cc1c2cc(ccc2on1)CN3CCCC(C3)c4cc(=O)[nH]cn4	-69.299
6	ZINC19782199	c1ccc(cc1)C(CC(=O)NCc2ccncn2)c3ccco3	-102.520	ZINC20906985	c1cc(cc(c1)C(=O)N2CCN(C(=O)C2)C3CCCC3)c4c[nH]nc4	-69.278
7	ZINC19224863	CC(Cn1cncn1)NC(=O)C2CCc3ccccc3C2	-101.869	ZINC22012024	c1cc(ccc1CCc2c[nH]c3c2c(=O)[nH]c(n3)N)C(=O)[O-]	-68.767
8	ZINC20820275	Cc1cc(on1)CC(=O)NCCc2nc(c3c(n2)CCCC3)C	-101.352	ZINC31285082	Cc1c(c(=O)[nH]c(n1)C)CC(=O)Nc2ccc3c(c2)[nH]c(=O)o3	-68.472
9	ZINC32540617	CC(C)n1cc(cn1)CNc2c(ncc(n2)c3cc4ccccc4o3)N	-99.204	ZINC12323067	c1cc2c(cc[nH]2)c(c1)OCC(=O)NCc3ccco3	-67.981
10	ZINC19228137	Cc1cc(nc(n1)CCNC(=O)Nc2[n-]nnn2)C(F)(F)F	-99.146	ZINC21512422	CCc1c(c(on1)C)C(=O)OCCCn2c3ccccc3[nH]c2=O	-67.955
11	ZINC05762780	c1cc(c(cc1Cl)Cl)c2[nH]nc(n2)Cn3cncn3	-98.961	ZINC32511762	Cc1ccc2c(c1)c(c[nH]2)c3cnc(c(n3)N4CCCC(C4)C#N)N	-67.626
12	ZINC12146860	Cc1nccn1CCNC(=O)c2cc([nH]n2)c3ccncc3	-98.932	ZINC04761086	c1c2c(cc(c1N3CCCCC3)F)nc(c(n2)N)C(=O)N	-67.556
13	ZINC32501669	c1ccc2c(c1)[nH]c(n2)SCc3[nH]c(nn3)c4ccccn4	-98.894	ZINC19238547	Cc1ccsc1C(=O)N2CCC(CC2)c3c4c([nH]n3)nccn4	-67.462
14	ZINC20136854	Cc1c(c2c(cn1)CN(CC2)c3ccnc(n3)C)c4nc(on4)C5CC5	-98.471	ZINC23399754	c1c2c(sc1C(=O)N3CCCC(C3)c4cc(=O)[nH]cn4)CCCC2	-67.367
15	ZINC23465885	c1ccc(cc1)Nc2ncc(cn2)C(=O)NCc3cc(=O)[nH]cn3	-98.090	ZINC17312810	C(CSN=Nc1[nH]c2c(n1)c(=O)[nH]c(n2)N)O	-67.306
16	ZINC20136854	Cc1c(c2c(cn1)CN(CC2)c3ccnc(n3)C)c4nc(on4)C5CC5	-97.447	ZINC17312810	C(CSN=Nc1[nH]c2c(n1)c(=O)[nH]c(n2)N)O	-67.306
17	ZINC14988217	C(CC(=O)[O-])c1nc(no1)c2nc(on2)CCC(=O)[O-]	-97.352	ZINC12792251	c1ccc2c(c1)nc(s2)c3ccc(o3)CNc4ccc5nnnn5n4	-67.037
18	ZINC20136854	Cc1c(c2c(cn1)CN(CC2)c3ccnc(n3)C)c4nc(on4)C5CC5	-97.281	ZINC16638552	CCCCCOC1[NH+]=C(N=C2N1c3ccccc3N2)N	-66.834
19	ZINC19744740	c1cc(cc(c1)F)c2c3c([nH]n2)CCN(C3)c4ccnc5c4cn[nH]5	-97.038	ZINC16638552	CCCCCOC1[NH+]=C(N=C2N1c3ccccc3N2)N	-66.834
20	ZINC29021867	c1ccc(cc1)c2cc(on2)COC(=O)c3cccnc3n4cncn4	-96.959	ZINC14768202	c1ccc2c(c1)c(c[nH]2)c3c[nH]c(n3)C(=O)c4c[nH]c5c4cccc5	-66.799
21	ZINC29036339	c1ccc(cc1)n2c(nnn2)NCc3ccc(nc3)n4ccnc4	-96.844	ZINC12147225	CCc1c(nc2cc(ccc2n1)C(=O)NCc3cc([nH]n3)C4CC4)CC	-66.792
22	ZINC23465967	Cc1ccc(s1)CNC(=O)Nc2nc([nH]n2)c3ccccc3F	-96.801	ZINC19939112	c1ccc(cc1)c2ccccc2Cn3cnc4c3c(=O)nc([nH]c4=O)N	-66.750
23	ZINC05135171	CCC(C(=O)[O-])n1c(=O)c2cc3c(n2cn1)cco3	-96.734	ZINC32741542	c1cc2cccc3c2c(c1)N(S3(=O)=O)CCSc4nc([nH]n4)N	-66.563
24	ZINC04772496	c1cc2c(cc1C=Cc3cnc4c(n3)c(nc(n4)N)N)OCO2	-96.616	ZINC06448865	c1ccc(cc1)c2nnc(o2)SCc3c(=O)[nH]c4ccccc4n3	-66.337
25	ZINC07719377	COc1ccc(c(c1)CC(=O)Nc2c(nc(nc2N)N)N)OC	-96.579	ZINC23250905	c1cc2c(cc1C(=O)N3CCCC(C3)c4cc(=O)[nH]cn4)ncs2	-66.324
26	ZINC23380475	Cc1[nH]c(=O)cc(n1)CCNC(=O)c2cc(n[nH]2)c3cccn3C	-96.496	ZINC14268197	c1cc(oc1)CCC(=O)Nc2ccc3c(c2)[nH]c(=O)[nH]3	-66.185
27	ZINC32547811	c1cc(ccc1c2ccc(cc2)C(=O)N)C(=O)c3[nH]ccn3	-96.328	ZINC12945191	c1ccc2c(c1)nc(o2)COC(=O)C3CC(=O)Nc4c3ccc(c4)F	-66.024
28	ZINC12360046	c1ccc2c(c1)C(=CS2(=O)=O)COC(=O)NCC(=O)[O-]	-96.267	ZINC14315130	c1ccc(cc1)n2cc(cn2)CNc3ccc4nnnn4n3	-65.909
29	ZINC32916933	c1ccc(cc1)c2c3c(c(ncn3)N4CCC(CC4)C(=O)[O-])on2	-95.963	ZINC35683323	c1ccc2c(c1)c(cc(=O)[nH]2)C(=O)OCn3cnc4c3cccc4	-65.829
30	ZINC19113065	Cc1nc(on1)c2ccc(nc2)c3cccc(c3)c4ccn(n4)CC=C	-95.698	ZINC19798798	c1cc(cc(c1)F)C2(CCOC2)CNc3ccc4c(c3)cn[nH]4	-65.342
31	ZINC19782117	Cc1cc(n(c(=O)c1)NC(=O)CCn2nc(nn2)c3ccccc3)C	-95.662	ZINC04810508	c1ccc(cc1)N(Cc2cc3ccccc3[nH]c2=O)C(=O)c4nc[nH]n4	-65.152
32	ZINC16951305	c1cc(ccc1C=Cc2cnc3c(n2)c(nc(n3)N)O)C(=O)[O-]	-95.651	ZINC26774818	c1ccc2c(c1)CCN2CCCNc3ccc4nnnn4n3	-64.981
33	ZINC15783075	CC(c1nc(no1)c2ccncc2)NC(=O)OCc3ccccc3	-95.589	ZINC05360588	COC(=O)c1ccc(o1)COC(=O)c2c3ccccc3[nH]n2	-64.972
34	ZINC10844543	c1cc(ccc1c2cnc(o2)SCc3nc(nc(n3)N)N)F	-95.525	ZINC05581275	c1cc2c(=O)c3cc4c(cc3[nH]c2nc1)[nH]c(=O)[nH]4	-64.964
35	ZINC20995358	c1ccc(cc1)N2CCC(C2)CNC(=O)c3cc(=O)[nH]c(n3)[O-]	-95.489	ZINC19771170	Cc1c(sc(n1)C)CC(=O)NCc2ccc3c(c2)[nH]c(=O)[nH]3	-64.944
36	ZINC14163274	c1ccc(cc1)n2c(c3c(n2)NC(=O)C(C3c4ccco4)C#N)O	-95.410	ZINC20915477	c1cc(c2cc[nH]c2c1)c3nc(on3)CCn4cncn4	-64.536
37	ZINC16689462	Cc1ccc2nc(nc(=O)n2c1)SCC(=O)NC3CCCCC3	-95.233	ZINC14123481	Cc1ccc2c(c1)occ2CC(=O)N3CC(=O)Nc4c3cccc4	-64.503
38	ZINC22002208	c1cc(ccc1C(=O)[O-])NCCCn2cnc3c2nc[nH]c3=O	-95.060	ZINC12146876	Cc1c(c(n[nH]1)C)CNC(=O)c2c3c(n[nH]2)CCCCC3	-64.430
39	ZINC20998451	c1ccc2c(c1)cnn2Cc3nc(no3)Cc4cccnc4	-95.044	ZINC09644478	c1ccc2c(c1)c3nc(nn3c(=O)[nH]2)Cn4cnc5c4cccc5	-64.141
40	ZINC30991478	Cn1ccsc1=NC(=O)CCC(=O)N2CC(=O)Nc3c2cccc3	-94.954	ZINC20982583	Cc1nn2c(=O)cc(nc2s1)CSc3nc([nH]n3)N	-64.030
41	ZINC32087639	Cc1c(c(=O)cco1)OCc2ccc(cc2)c3nc(on3)C(=O)N	-94.860	ZINC23580734	c1ccc(cc1)c2c([nH]cn2)c3cccc(c3)c4cc[nH]n4	-63.947
42	ZINC16079956	c1ccc2c(c1)ncc(n2)c3cc(c(nc3c4ccncc4)O)C#N	-94.834	ZINC19719481	Cn1cc(cn1)c2cncn2C3CC(=O)N(C3)c4ccc(cc4)OC	-63.822
43	ZINC30028008	c1ccc(cc1)c2cc(n[nH]2)C(=O)OCc3nnnn3C4CC4	-94.694	ZINC07625220	Cc1cc(nc(n1)SCc2nc(nc(n2)N)N)c3ccccc3	-63.708
44	ZINC12561239	Cc1nc2ccc(cc2s1)Nc3ccc4nnnn4n3	-94.692	ZINC20452692	c1cc(ccc1CCNCc2cc(ccc2O)c3cnccn3)F	-63.645
45	ZINC23311107	c1cc(oc1)c2nnc(s2)NC(=O)NCc3c4c([nH]n3)CCC4	-94.663	ZINC23214524	CC1Cc2cc(ccc2O1)C(=O)N3CCC(CC3)c4cc(=O)[nH]cn4	-63.539
46	ZINC19117721	Cc1cc(nc(n1)C2CCCN(C2)C(=O)Cc3ccccn3)O	-94.431	ZINC32741542	c1cc2cccc3c2c(c1)N(S3(=O)=O)CCSc4nc([nH]n4)N	-63.339
47	ZINC26421097	CC1Cc2cc(ccc2O1)c3cc(no3)C(=O)[O-]	-94.370	ZINC22002208	c1cc(ccc1C(=O)[O-])NCCCn2cnc3c2nc[nH]c3=O	-63.290
48	ZINC19655651	Cc1c(c(nc(n1)N)C)CCC(=O)N(C)CC2(COC2)C	-94.327	ZINC11616082	c1cc(cnc1)c2ccc(c(n2)SCc3cc(=O)[nH]c(=O)[nH]3)C#N	-63.256
49	ZINC05957007	COC(=O)c1ccc(cc1)COC(=O)N2C=CCC2C(=O)[O-]	-94.240	ZINC32598229	c1ccc(c(c1)CNc2cnccn2)n3cncn3	-63.172
50	ZINC11492191	Cc1ccc(cc1n2cccc2)C(=O)OCc3nc(nc(n3)N)N	-94.192	ZINC23145872	Cc1[nH]c(=O)cc(n1)CCNC(=O)c2cn(c3c2cccc3)C	-63.046
51	ZINC21982687	c1ccc(cc1)C(=O)OCC2OCC(O2)C(=O)[O-]	-93.701	ZINC20166597	c1ccc2c(c1)-c3ccccc3C2NC(=O)C4CCCN(C4)C(=O)N	-62.907
52	ZINC29020405	CCc1nnc(o1)COC(=O)c2ccc(cc2)c3nc(no3)C	-93.670	ZINC15225635	c1cc2c(cc1Oc3ccc4c(c3)nc(c(n4)O)O)nc(c(n2)O)O	-62.690
53	ZINC14768202	c1ccc2c(c1)c(c[nH]2)c3c[nH]c(n3)C(=O)c4c[nH]c5c4cccc5	-93.668	ZINC08625737	CCCCOC(=O)c1cc(=O)[nH]c2c1cccc2	-62.642
54	ZINC13312152	Cc1cc(oc(=O)c1c2cc(cc(c2C(=O)[O-])O)OC)C(=O)[O-]	-93.614	ZINC09264647	c1ccc(cc1)Nc2[nH]c(=O)cc(n2)CSc3ncccn3	-62.608
55	ZINC04830524	c1cc(ccc1N)SCCCc2c(nc(nc2N)N)N	-93.613	ZINC32561768	CCc1c2c([nH]n1)CCN(C2)C(=O)c3cccc(c3)c4ccc(o4)C	-62.512
56	ZINC13693309	Cc1cc(n(c(=O)c1c2nc(on2)C(=O)[O-])c3cccc(c3)OC)C	-93.567	ZINC08586194	c1ccc2c(c1)c(cc(=O)[nH]2)CSc3ccccn3	-62.436
57	ZINC05531203	c1cc2c3cc4c(=O)[nH]c(=O)nc4oc3cc5c2c(c1)c(=O)oc5=O	-93.552	ZINC30005705	CC(c1cc2ccccc2o1)N(C)Cc3nnnn3Cc4ccccc4	-62.346
58	ZINC19124373	Cc1cc(on1)c2cncnc2c3ccc(nc3)N4CCOCC4	-93.339	ZINC11658316	Cc1nc([nH]n1)SCC(=O)c2ccc3c(c2)-c4ccccc4C3	-62.301
59	ZINC20136854	Cc1c(c2c(cn1)CN(CC2)c3ccnc(n3)C)c4nc(on4)C5CC5	-93.323	ZINC19112822	Cc1c[nH]c(n1)CN(C)C(=O)c2cccc(c2)c3c[nH]nc3	-62.253
60	ZINC19111859	CC(=C)Cn1cnc2c1c(=O)nc([nH]c2=O)Nc3ccc(cc3)Cl	-93.297	ZINC32937714	c1cc(ccc1CNC(=O)NC2CCCC2)c3ccn[nH]3	-62.215
61	ZINC04997834	Cc1ccc(cc1)C(=O)CC(C(=O)[O-])n2cncn2	-93.277	ZINC19744740	c1cc(cc(c1)F)c2c3c([nH]n2)CCN(C3)c4ccnc5c4cn[nH]5	-62.197
62	ZINC14805447	c1ccc(cc1)n2cnc(n2)SCc3nc(nc(n3)N)N	-93.168	ZINC20530255	c1ccc(cc1)c2c3ccccc3[nH]c(=O)c2SCCO	-62.188
63	ZINC35426082	c1cnc(nc1)N2CCCC2c3nc(no3)c4cncnc4N	-93.156	ZINC32511762	Cc1ccc2c(c1)c(c[nH]2)c3cnc(c(n3)N4CCCC(C4)C#N)N	-62.036
64	ZINC27820520	c1cc(nnc1n2ccnc2)N3CCC(CC3)C(=O)[O-]	-93.141	ZINC11107116	c1cc(sc1)CCn2cnnc2SCc3nc(nc(n3)N)N	-61.943
65	ZINC20779847	Cc1ccccc1SCC(=O)NC(C)c2ccncn2	-93.041	ZINC17024390	CCc1c2c3ccccc3[nH]c2nc(n1)O	-61.934
66	ZINC04963604	c1cc(ccc1C(=O)[O-])NCC(=O)Nc2c(nc(nc2N)N)N	-92.988	ZINC19455592	CC(CCCc1ccccc1)NC(=O)c2cc3c([nH]c2=O)CCC3	-61.891
67	ZINC32587203	c1cc2c(cc1c3ccc4c(c3)OCCO4)c(=O)[nH]cn2	-92.904	ZINC23141629	c1ccc2c(c1)cc(o2)CCC(=O)Nc3[n-]cnn3	-61.834
68	ZINC07373202	Cc1cc(no1)COC(=O)c2ccc3c(c2)NC(=O)C4N3CCC4	-92.899	ZINC22009138	CNS(=O)(=O)Cc1ccc2c(c1)c(c[nH]2)Sc3ccccc3	-61.828
69	ZINC09117564	c1ccc(cc1)n2cc(cn2)C(=O)OCc3nc4ccccc4c(n3)N	-92.875	ZINC25562121	c1cc(cc(c1)SCCOC(=O)c2cccnc2N)C(F)(F)F	-61.828
70	ZINC19337867	Cc1cc(nc(n1)CCNC(=O)C(C)n2ccnc2C)C	-92.837	ZINC23429798	Cc1c(c(n(n1)c2ccccc2)C)Cc3cc(n4c(n3)nc(n4)C)[O-]	-61.809
71	ZINC14119639	c1ccc(cc1)Cn2cc(c(n2)c3ccc4c(c3)OCCCO4)CO	-92.829	ZINC20865973	c1cc(sc1)c2nc(cs2)CN3CCc4c(cn[nH]4)C3	-61.806
72	ZINC19369061	c1ccc(cc1)C(=O)NCCn2c(=O)cc3c(n2)CCCCC3	-92.825	ZINC19938818	c1ccc(cc1)C2CCCN(CC2)C(=O)c3c[nH]c(=O)[nH]c3=O	-61.731
73	ZINC17058506	c1ccc(cc1)CCC2=Nc3c(nc(nc3OC2)N)O	-92.780	ZINC05661495	COc1ccc(cc1)C(=O)c2cc([nH]c2)C(=O)NCc3ccco3	-61.701
74	ZINC06411783	COc1ccc-2cc-3n(c(c2c1=O)O)CCc4c3cc5c(c4)OCO5	-92.762	ZINC01620723	Cn1c(nc2c1c(=O)[nH]c(=O)n2C)Cc3ccccc3	-61.689
75	ZINC12195325	Cc1ccc(cc1)c2nc(no2)CNC(=O)c3c(onc3C(C)C)C	-92.759	ZINC23380348	Cc1cccc(c1)n2cc(cn2)c3ccnc(n3)NCc4cc(on4)C	-61.680
76	ZINC15729907	c1ccc2c(c1)cc([nH]2)c3nc(no3)CN4c5ccccc5OCC4=O	-92.751	ZINC20534310	c1ccc2c(c1)c(n[nH]2)CC(=O)NC3CC4(CCCC4)OC3	-61.625
77	ZINC09437918	COc1cc(ccc1OCc2cc(=O)oc3c2cc4c(c3)CCC4)C#N	-92.742	ZINC12815164	Cc1cc(n2c(n1)ncn2)NCc3ccc(s3)c4csc(n4)C	-61.530
78	ZINC32587099	Cc1ncc(cn1)CNc2ccc(cc2)N3CCOCC3	-92.742	ZINC32582029	CCc1c(nco1)C(=O)N2CCC(CC2)c3cc(=O)[nH]c(n3)C	-61.513
79	ZINC20233833	c1cc(c(cc1C(F)(F)F)C(=O)NCCCc2[n-]nnn2)Cl	-92.599	ZINC32541020	Cc1cccc2c1ncn(c2=O)CCSc3[nH]ncn3	-61.481
80	ZINC20536424	CCC(C)(C)c1nnc(s1)NC(=O)NC(C)Cn2cncn2	-92.555	ZINC05090674	COC(=O)c1ccc(o1)COC(=O)c2c[nH]c3c2cccc3	-61.363
81	ZINC06448865	c1ccc(cc1)c2nnc(o2)SCc3c(=O)[nH]c4ccccc4n3	-92.530	ZINC08773480	Cn1cccc1C(=O)OCC(=O)c2ccc3c(c2)[nH]c(=O)[nH]3	-61.296
82	ZINC08733302	c1cc2c(cc1c3cc(no3)C(=O)[O-])OCCCO2	-92.449	ZINC08770056	Cc1c(c2cc(ccc2[nH]1)F)CCCNC(=O)c3cccs3	-61.285
83	ZINC29752537	Cc1nc(on1)COc2cccc(c2)c3nnco3	-92.406	ZINC12446906	Cc1cc2c(c(=O)[nH]1)C(=O)OC2=Cc3c[nH]nc3c4cccs4	-61.267
84	ZINC32511656	CC(c1cnn(c1)c2ccc(cc2)OC)NC(=O)Nc3nncs3	-92.364	ZINC19339616	c1cc(cnc1)CN2CC(C(C2)c3cc[nH]n3)c4ccc(cc4)F	-61.222
85	ZINC09290290	Cc1cnc(o1)NC(=O)CSc2[nH]c3ccc(cc3n2)OC	-92.320	ZINC30998725	CN(CCCNC(=O)NC1CCCC1)c2ccccc2	-61.095
86	ZINC27660342	c1ccc(cc1)c2c[nH]c(n2)SCc3nc(nc(n3)N)N	-92.290	ZINC19835578	c1ccc2c(c1)c(n[nH]2)c3nc(no3)Cc4cccnc4	-61.089
87	ZINC20943066	Cn1c2ccc(cc2oc1=O)Nc3nc4ccccc4c5n3cnn5	-92.231	ZINC17885173	c1ccc2c(c1)ccc(n2)Cc3c(=O)[nH]c4ccccc4n3	-61.049
88	ZINC20638614	c1ccc2c(c1)CCC(C2)C(=O)NCCc3[nH]c(=O)cc(n3)N	-92.213	ZINC07844423	Cc1cc(c(n1Cc2ccc3c(c2)OCO3)C)c4csc(n4)NC	-60.906
89	ZINC09167821	Cc1nnc(s1)NC(=O)c2ccc3c(c2)[nH]c(=O)c(=O)[nH]3	-92.177	ZINC32741542	c1cc2cccc3c2c(c1)N(S3(=O)=O)CCSc4nc([nH]n4)N	-60.805
90	ZINC35182979	c1ccc2c(c1)c(c[nH]2)CC(C(=O)[O-])NC(=O)c3[n-]ncn3	-92.110	ZINC24990210	c1ccc(cc1)c2nc(cs2)CNc3ccc4nnnn4n3	-60.717
91	ZINC09337388	c1ccc(cc1)Cn2cc(nn2)C=CC3Nc4ccccc4C(=O)N3	-92.092	ZINC23144715	c1ccc(cc1)c2[nH]cc(n2)C(=O)NCCc3cc(=O)[nH]cn3	-60.716
92	ZINC16976456	CCOC(=O)c1ccc(cc1)NCc2nc3c(nc(nc3O)N)nn2	-92.074	ZINC28334853	c1cc2c(cc1c3csc(n3)C4CC4)[nH]c(=O)[nH]2	-60.691
93	ZINC23153533	c1ccc2c(c1)nc(o2)CNC(=O)Nc3[n-]c(nn3)C4CCCCC4	-92.060	ZINC08830788	Cc1cc(=O)[nH]c2c1cc3c4ccccc4oc3c2	-60.684
94	ZINC12955905	c1cc(ccc1n2cnc(n2)C(=O)[O-])OC(F)F	-92.003	ZINC07998246	c1ccc(cc1)c2cnc(o2)c3ccccc3C(=O)OCC(=O)N	-60.662
95	ZINC05195095	c1cc(ccc1N2CC(CC2=O)NC(=O)Nc3nccs3)F	-91.977	ZINC14143248	c1ccc-2c(c1)Cc3c2cc(cc3)C(=O)Cn4cnc(n4)C#N	-60.647
96	ZINC16044906	Cc1c2cc(sc2n(n1)Cc3ccccc3)C(=O)N=c4[n-][nH]nn4	-91.960	ZINC29585261	c1ccc2c(c1)nc(s2)NCc3cccc(c3)Cn4ccnc4	-60.643
97	ZINC32507802	c1ccc(cc1)C2(CCN(C2)c3c(ncc(n3)c4cc[nH]n4)N)O	-91.905	ZINC31732826	COc1ccc(cc1O)C=Cc2c(ccc(n2)C(=O)[O-])C#N	-60.636
98	ZINC20869223	c1cc(sc1)c2ccc(o2)CCC(=O)NCc3ccncn3	-91.816	ZINC11872275	CC(=O)c1ccc(s1)c2cc(cc3c2OC(C3)CNC(=O)C)F	-60.627
99	ZINC20999362	CCc1nc(nn1C)NC(=O)Cn2c(=O)cc(cn2)N3CCCC3	-91.755	ZINC12404618	c1cc2c(cc1S(=O)(=O)N3CCCC3)c4c([nH]2)nc(nn4)N	-60.553
100	ZINC12859203	Cc1[nH]c(nn1)SCc2csc(n2)COc3ccc(cc3)F	-91.753	ZINC08341374	c1cc(sc1)C2CCCN2C(=O)c3cnccn3	-60.524

*ZINC ID: ZINC library compund identification

**SMILES: simplified molecular-input line-entry system

	EPSP			FabG		
Rank	ZINC ID	Smiles	Score	ZINC ID	Smiles	Score
1	ZINC04934094	c1c([nH]c(=O)[nH]c1=O)C(C(C(C(CO)O)O)O)O	-95.902	ZINC25049626	Cn1c2c(cn1)c(ncn2)Sc3nnc(n3c4ccccc4)C5CC5	-84.795
2	ZINC16951493	CC(=O)Nc1nnc(s1)Sc2c(nc(nn2)O)O	-82.306	ZINC31147705	Cc1c(c2ccccc2[nH]1)CC(=O)OCc3nnnn3c4ccccc4	-84.167
3	ZINC05368651	C1C2(CC1(C(=O)[O-])S(=O)(=O)[O-])CC(C2)(C(=O)[O-])S(=O)(=O)[C	-79.841	ZINC20866409	CCCn1c(c(cn1)c2ccnc(n2)NCc3cnc4n3cccc4)C	-83.177
4	ZINC08615340	CSc1nc(cc(n1)Cl)Sc2c3c([nH]cn3)nc(n2)N	-79.543	ZINC23337892	Cc1c(c(on1)C)c2c3c([nH]n2)CCN(C3)C(=O)c4ccc(nc4)C#N	-81.424
5	ZINC20267753	c1cc2c(cc1S(=O)(=O)NCC(=O)[O-])nnn2[O-]	-78.254	ZINC16847028	c1ccc(cc1)c2c(c[nH]n2)c3nc(no3)c4ccccn4	-81.189
6	ZINC32500135	c1nnnn1CCCCCC(=O)[O-]	-76.243	ZINC12603896	CSc1[nH]c(=O)cc(n1)COC(=O)c2c3ccccc3[nH]n2	-81.183
7	ZINC26807968	C(CC(=O)Nc1nc([nH]n1)C(=O)[O-])CC(=O)[O-]	-74.921	ZINC12192754	Cc1c(c(nc(n1)OC)OC)CC(=O)Nc2c[nH]c3c2cccc3	-80.997
8	ZINC12395294	Cc1cc(c2c(nn(c2n1)CC(=O)[O-])C)C(F)F	-74.884	ZINC14240853	c1ccc2c(c1)c(n[nH]c2=O)CC(=O)Nn3cnc4c3cccc4	-79.899
9	ZINC17128094	c1nc(c2c(n1)[nH]c(n2)C(C(C(CO)O)O)O)N	-74.753	ZINC01642034	Cc1c(c(c2cc(ccc2n1)Cl)n3ccnc3)CCn4ccnc4	-78.992
10	ZINC12139034	c1cnc(nc1SCC(=O)[O-])SCC(=O)[O-]	-74.667	ZINC23369462	CC1CNC(=O)CCN1C(=O)Nc2nnc(s2)Cc3ccccc3	-78.694
11	ZINC16136382	C(Nc1c(non1)C(=O)[O-])Nc2c(non2)C(=O)[O-]	-74.389	ZINC23380336	CC(C)(C)c1c2c([nH]n1)CCN(C2)C(=O)CCn3cccn3	-78.690
12	ZINC12397865	Cn1cc(cn1)S(=O)(=O)n2ccc(n2)C(=O)[O-]	-73.965	ZINC19553306	COc1ccc(cc1)C(c2c3cccnc3cc(n2)c4ccncc4)O	-78.515
13	ZINC21587944	CCc1nc([nH]n1)SCc2nc(nc(n2)N)N	-72.969	ZINC23145963	CCCSc1ncc(cn1)CN2CCc3c(c(n[nH]3)C4CCC4)C2	-78.335
14	ZINC19702666	COCCn1c2c(c(=O)[nH]c1=O)C(CC(=O)N2)C(=O)[O-]	-72.857	ZINC12937612	c1ccc2c(c1)c(nc(n2)c3ccncc3)Sc4nc([nH]n4)C5CC5	-78.330
15	ZINC15572960	c1cc(=O)n(cc1C(F)(F)F)Cc2nc(nc(n2)N)N	-72.805	ZINC30795010	c1ccc2c(c1)c(=O)n(nn2)CC(=O)N3CCn4c3nc5c4cccc5	-77.805
16	ZINC17046269	c1c2c(nnnc2O)n(n1)C3C(C(C(O3)CO)O)O	-72.614	ZINC21351769	Cc1cc(c(=O)[nH]n1)Cc2ccc(cc2)S(=O)(=O)NC3CC3	-77.797
17	ZINC04715143	CC(c1ccccc1)Oc2c3c(nc(n2)N)n[nH]n3	-72.534	ZINC05394157	Cc1c(c2nc(c(c(n2n1)c3cccnc3)C#N)N)c4ccccc4	-77.576
18	ZINC08651763	CC1C(C(C(C(O1)Sc2c3c(nc[nH]3)ncn2)O)O)O	-72.326	ZINC12148420	c1ccc2c(c1)CCN2CCNC(=O)COc3c4ccccc4ncn3	-77.526
19	ZINC05605106	c1ccc2c(c1)nnn2CNc3c(non3)CC(=O)[O-]	-72.108	ZINC26480220	CCCn1c(nnn1)COC(=O)Cn2c(=O)c3ccccc3nn2	-77.480
20	ZINC20760149	c1cc2c(cc1S(=O)(=O)CCC(=O)[O-])oc(n2)[O-]	-71.303	ZINC20536625	c1cnc(cn1)c2nc(on2)c3c[nH]nc3C4CCCCC4	-77.404
21	ZINC27444369	Cc1nc(no1)CSc2c3ccccc3ncn2	-70.946	ZINC13115225	CC1=CC(N(c2c1cccc2)C(=O)CSc3[nH]ncn3)(C)C	-77.333
22	ZINC16957304	c1c2n(c(=O)[nH]c1=O)C3C(C(C(C2CCO)O3)O)O	-70.547	ZINC15729907	c1ccc2c(c1)cc([nH]2)c3nc(no3)CN4c5ccccc5OCC4=O	-77.252
23	ZINC18240389	Cc1c([nH]c(=O)[nH]1)C(=O)CCCCC(=O)[O-]	-70.243	ZINC23159102	COc1c(cnc(n1)OC)CN2CCc3c(c(n[nH]3)C4CCCC4)C2	-77.113
24	ZINC18240389	Cc1c([nH]c(=O)[nH]1)C(=O)CCCCC(=O)[O-]	-70.243	ZINC09594751	Cc1ccccc1n2cnc3c2ccc(c3)NCc4ccccn4	-77.032
25	ZINC12653947	c1cc(oc1Cc2c(nc(nc2O)N)O)C(=O)[O-]	-70.107	ZINC23145963	CCCSc1ncc(cn1)CN2CCc3c(c(n[nH]3)C4CCC4)C2	-76.941
26	ZINC15230584	c1c(c(nc(=O)n1CC(C(=O)[O-])O)N)Br	-69.636	ZINC04632203	Cc1c(nc2ccccc2n1)CSc3c4c([nH]cn4)ncn3	-76.896
27	ZINC20411131	c1[nH]c2c(n1)nc(nc2[O-])NC(CC(=O)[O-])C(=O)[O-]	-69.619	ZINC32895845	CCOC(=O)c1cn[nH]c1S(=O)(=O)N2CCCc3c2ccc(c3)C	-76.731
28	ZINC13598349	C(COCCc1[n-]nnn1)c2[n-]nnn2	-69.609	ZINC23399754	c1c2c(sc1C(=O)N3CCCC(C3)c4cc(=O)[nH]cn4)CCCC2	-76.712
29	ZINC13683478	c1csc(c1N)c2nc(c(c(n2)O)O)C(=O)[O-]	-69.578	ZINC08318008	Cn1c2ccccc2nc1Sc3nnnn3c4ccccc4	-76.600
30	ZINC07996477	CCOC(=O)C1CCN(CC1)c2cn[nH]c(=O)n2	-69.432	ZINC08295621	Cc1ccc(c(c1)C(=O)Cn2c(nnn2)c3ccccc3)C	-76.596
31	ZINC12949651	CN(C)c1c2c(ncn1)Sc3c(c(ncn3)OC)C=N2	-69.151	ZINC19340918	c1cc(cnc1)c2[nH]c(=O)cc(n2)CNC(=O)c3cccnc3	-76.590
32	ZINC05059888	Cc1c(non1)NC(=O)CSc2nnnn2C	-68.974	ZINC23506685	c1cc(sc1)c2c(cn[nH]2)C(=O)NCCc3cc(=O)[nH]cn3	-76.504
33	ZINC26684381	Cc1cc(on1)C(=O)OCc2nc(nc(n2)N)N	-68.835	ZINC13247129	c1cc(cc(c1)S(=O)(=O)NCCCc2c(c(n[nH]2)N)C#N)C(=O)[O-]	-76.482
34	ZINC17189243	c1cc2c(c(c1)NC3C(C(C(O3)CO)O)O)ncnc2O	-68.799	ZINC19719581	CC(C)(CCCn1cncc1c2c[nH]nc2c3ccccc3)CO	-76.428
35	ZINC19795061	CN(Cc1ccncn1)C(=O)CCc2c3c(n[nH]2)CCCC3	-68.791	ZINC12903140	c1ccc(cc1)n2c(nnn2)COC(=O)c3c4ccccc4c(=O)[nH]n3	-76.409
36	ZINC17176208	C1CC(CC1CO)n2c3c(c(=O)nc([nH]3)N)nn2	-68.659	ZINC09117564	c1ccc(cc1)n2cc(cn2)C(=O)OCc3nc4ccccc4c(n3)N	-76.400
37	ZINC06062827	c1ccn(c1)c2c(non2)NC(=O)CSc3[nH]ncn3	-68.556	ZINC12559916	c1cc2c(cc1C(=O)NCCCc3c(c([nH]n3)N)C#N)CCCC2	-76.317
38	ZINC05009408	c1[nH]c2c(n1)c(ncn2)Sc3c(=O)[nH]c(=O)[nH]n3	-68.418	ZINC20209921	Cc1ccc2c(c1)CC(N2)C(=O)NCCc3[nH]nc(n3)c4cccnc4	-76.281
39	ZINC13892931	Cn1c2c(c(=O)n(c1=O)C)n(cn2)Cc3[n-]nnn3	-68.334	ZINC23541961	CC(C)c1c2c([nH]n1)CCN(C2)Cc3ccc(cc3)n4cncn4	-76.256
40	ZINC12397546	Cn1cc(c(n1)C(=O)Nc2ncn(n2)CC(=O)[O-])C(=O)[O-]	-68.163	ZINC26482738	c1ccc(cc1)n2c(nnn2)COC(=O)c3ccc4c(c3)nccn4	-76.215
41	ZINC21992231	c1cc(c(cc1N)NCCC(=O)[O-])S(=O)(=O)[O-]	-68.152	ZINC23250739	COCc1c2c([nH]n1)CCN(C2)C(=O)c3cc(n[nH]3)c4ccsc4	-76.215
42	ZINC25454593	CCc1nc([nH]n1)Sc2c3c(ccs3)ncn2	-68.045	ZINC33082933	Cc1ccc(cc1n2cnnn2)NC(=O)Cn3ccc(=O)[nH]c3=O	-76.203
43	ZINC14983655	c1(c(c(nc(c1Cl)O)C(=O)[O-])Cl)Cl	-68.017	ZINC14768202	c1ccc2c(c1)c(c[nH]2)c3c[nH]c(n3)C(=O)c4c[nH]c5c4cccc5	-76.188
44	ZINC23142787	c1ccc2c(c1)c(n[nH]c2=O)C(=O)Nc3nc[nH]n3	-67.995	ZINC12433934	c1ccc(cc1)CCn2cc(nn2)C(=O)N3CCc4c(cn[nH]4)C3	-76.186
45	ZINC05674936	CN(C1C(C(C(C(O1)CO)O)O)O)C(=O)c2[nH]ncn2	-67.785	ZINC19222149	CN(CCc1ccccc1)Cc2nc(no2)c3cnccn3	-76.095
46	ZINC06982402	c1cc(oc1)C(=O)CSc2[nH]c(cc(=O)n2)N	-67.761	ZINC30773513	c1cc(c(nc1)N2CCC(CC2)C(=O)c3ccc4c(c3)OCCO4)C#N	-76.063
47	ZINC04710712	c1ccc(c(c1)c2[n-]nnn2)NCc3cccnc3	-67.682	ZINC20599645	Cc1nc([nH]n1)CNC(=O)C(C)(C)C(c2ccccc2)O	-75.986
48	ZINC14982340	CCCCc1ccnc(c1)C(=O)[O-]	-67.298	ZINC20096313	c1ccc(c(c1)C(=O)Nc2ccc3c(c2)c(n[nH]c3=O)[O-])C(=O)[O-]	-75.981
49	ZINC06553415	Cc1nnc(o1)Cn2c(=O)c3ccccc3c(n2)C(=O)[O-]	-67.262	ZINC19846638	c1ccc(cc1)n2c(nnn2)Sc3c(cccn3)C#N	-75.963
50	ZINC04681107	COCCNC(=O)CCC(=O)[O-]	-67.054	ZINC08135845	c1ccc2c(c1)c(c[nH]2)c3csc(n3)NC(=O)c4ccc(=O)[nH]c4	-75.851
51	ZINC32617587	Cc1ccc(cc1)CNc2cc(nn3c2nnc3)C	-66.951	ZINC32561768	CCc1c2c([nH]n1)CCN(C2)C(=O)c3cccc(c3)c4ccc(o4)C	-75.731
52	ZINC17111814	Cn1c2c(c(=O)n(c1=O)C)nc(c(n2)O)C(=O)[O-]	-66.837	ZINC16363974	Cn1c2ccc(cc2n(c1=O)C)Nc3c4nncn4c5ccccc5n3	-75.650
53	ZINC04940015	c1ccc2c(c1)n(c(=O)o2)CC(=O)Nc3[n-]nnn3	-66.830	ZINC12116229	Cc1c(c[nH]n1)C(=O)N2CCCC(C2)c3[nH]c(=O)c4ccccc4n3	-75.638
54	ZINC05594786	c1nc2c(c(n1)N)snc2C3C(C(C(O3)CO)O)O	-66.798	ZINC05027683	c1ccc(cc1)S(=O)(=O)NCCSc2c3c([nH]cn3)ncn2	-75.624
55	ZINC05466807	CCCCn1c(nnn1)CSc2[nH]ncn2	-66.797	ZINC08201425	c1csc2c1c(ncn2)Sc3nnc(o3)CN4CCCC4=O	-75.602
56	ZINC06403608	COc1cc(cc(c1OC)OC)C(Cc2ccnnc2)O	-66.772	ZINC23465852	CCOC(=O)c1c([nH]c(=O)[nH]1)CN2CCc3c(c(n[nH]3)C(C)C)C2	-75.544
57	ZINC20385514	CSc1ncc(c(n1)NCc2ccccc2)C(=O)[O-]	-66.623	ZINC23145963	CCCSc1ncc(cn1)CN2CCc3c(c(n[nH]3)C4CCC4)C2	-75.436
58	ZINC05004726	CC1C(NC(=O)N1)CCCCC(C)C(=O)[O-]	-66.591	ZINC12203826	c1ccc(c(c1)CNc2nc(cnn2)c3cccc(c3)F)n4cncn4	-75.368
59	ZINC17146836	Cn1c2c(c(=O)n(c1=O)C)[nH]c(n2)CNC(=O)N	-66.527	ZINC23465852	CCOC(=O)c1c([nH]c(=O)[nH]1)CN2CCc3c(c(n[nH]3)C(C)C)C2	-75.317
60	ZINC05416409	Cc1ccc(cc1)C(c2ccnn2Cn3cccn3)O	-66.511	ZINC27228886	c1ccc2c(c1)[nH]c(n2)CCC(=O)Nc3cccc(c3)n4cnnn4	-75.288
61	ZINC21455652	c1[nH]c(nn1)SCc2c(nnn2c3c(non3)N)C(=O)[O-]	-66.447	ZINC19722847	c1ccc(cc1)c2c(cn[nH]2)CN3CCc4ccccc4C3	-75.276
62	ZINC08745690	Cc1nnc(o1)CSc2c3c(ccs3)ncn2	-66.364	ZINC23145963	CCCSc1ncc(cn1)CN2CCc3c(c(n[nH]3)C4CCC4)C2	-75.275
63	ZINC12476511	Cc1cc(nc(c1C#N)SCc2nc(nc(n2)N(C)C)N)C	-66.348	ZINC05422580	CCOC(=O)c1ccc(cc1)NCC2=Nc3c(nc(nc3NC2)N)N	-75.264
64	ZINC05178463	C(CC(=O)[O-])C=CCCC(=O)[O-]	-66.282	ZINC23232249	c1cc2c(cc1Cc3cc(n4c(n3)cc(n4)C5CCC5)[O-])NC(=O)CO2	-75.232
65	ZINC23140934	CCCc1nc(on1)CCC(=O)Nc2[n-]cnn2	-66.174	ZINC32934781	c1ccc2c(c1)[nH]c(n2)CCC(=O)Nc3[nH]nc(n3)c4ccccn4	-75.084
66	ZINC06485842	c1cc2c(c(c1)S(=O)(=O)NCc3cccnc3)non2	-66.132	ZINC23159102	COc1c(cnc(n1)OC)CN2CCc3c(c(n[nH]3)C4CCCC4)C2	-75.035
67	ZINC12722599	CN(C)c1nc(nc(n1)N)CSc2c3ccsc3ncn2	-66.124	ZINC13214435	CC(=Cc1c[nH]c2c1cc(cc2)OC)c3ccncc3	-74.945
68	ZINC15224539	COc1ccccc1Cc2c(c(=O)[nH]c(=O)[nH]2)Br	-66.040	ZINC23145963	CCCSc1ncc(cn1)CN2CCc3c(c(n[nH]3)C4CCC4)C2	-74.931
69	ZINC12880450	c1ccc2c(c1)c(c[nH]2)C(=O)Cn3cnc(n3)C#N	-65.988	ZINC12196682	Cc1c(cnc(n1)c2ccncc2)C(C)NC(=O)Cc3cccs3	-74.930
70	ZINC06591280	c1cn(c(=O)[nH]c1=O)C2(C=C(C(C2O)O)CO)CO	-65.909	ZINC23159102	COc1c(cnc(n1)OC)CN2CCc3c(c(n[nH]3)C4CCCC4)C2	-74.908
71	ZINC05544208	c1c2c(c(nn1)N)n(cn2)C3C(C(C(O3)CO)O)O	-65.789	ZINC23337581	CCc1c2c([nH]n1)CCN(C2)Cc3cnc(nc3)c4ccccn4	-74.844
72	ZINC08608094	c1cc2c(nc1)n(cn2)Cc3ccc(cc3)C(=O)[O-]	-65.734	ZINC23368304	CC(C)CCc1c2c([nH]n1)CCN(C2)C(=O)c3nnc4n3CCCC4	-74.817
73	ZINC24508885	CCn1c(nnn1)SCc2nc(nc(n2)N)N	-65.595	ZINC19731956	c1ccc(cc1)N2CCC(CC2)NC(=O)CCc3ccncc3	-74.798
74	ZINC16983116	c1nc2c(n1C3CC(C(O3)CO)O)nnnc2O	-65.509	ZINC20405067	Cn1c2c(cn1)c(c(cn2)CO)NCc3ccc(cc3)OC	-74.752
75	ZINC19872949	C=CCOc1ccc(nc1Br)C(=O)[O-]	-65.468	ZINC28560418	CNC(=O)c1ccc(o1)Cn2c(=O)c3cc4c(cc3nn2)OCCO4	-74.720
76	ZINC26640365	Cc1nc(on1)Cn2c(=O)n(c(=O)n(c2=O)CC=C)CC=C	-65.435	ZINC23337581	CCc1c2c([nH]n1)CCN(C2)Cc3cnc(nc3)c4ccccn4	-74.706
77	ZINC21298278	COc1c(ccnc1C(=O)[O-])OCC(F)(F)F	-65.376	ZINC33380076	c1ccc2c(c1)c(c[nH]2)C(C(=O)[O-])NC(=O)c3cc(=O)[nH]c(n3)[O-]	-74.678
78	ZINC13739513	C1(C(N(C(C(N1S(=O)(=O)O)O)O)O)S(=O)(=O)O)O)O	-65.376	ZINC06188436	c1ccc2c(c1)cnnc2SCc3c(=O)[nH]c4ccccc4n3	-74.645
79	ZINC05437940	c1c(c(n(n1)C2C(C(C(O2)CO)O)O)C(=O)N)N	-65.255	ZINC06720024	c1ccc2c(c1)CCN2S(=O)(=O)NC(=O)c3c[nH]c4c3cccc4	-74.632
80	ZINC32534861	CC1c2cccn2CCN1c3c4cn[nH]c4nc(n3)N	-65.026	ZINC23145963	CCCSc1ncc(cn1)CN2CCc3c(c(n[nH]3)C4CCC4)C2	-74.621
81	ZINC05798584	CC1Cc2c3c(cccc3N1)ncn2	-65.026	ZINC20909120	CCCc1c2c([nH]n1)CCN(C2)S(=O)(=O)c3cc4ccccc4o3	-74.554
82	ZINC12652408	C(C1C(C(C(C(O1)Sc2c(=O)[nH]c(=O)[nH]n2)O)O)O)O	-64.990	ZINC24618083	c1cnc(cn1)N2CCN(CC2)C(=O)CSc3[nH]ncn3	-74.544
83	ZINC32916887	c1ccc(cc1)S(=O)(=O)Cc2nc(on2)CCC(=O)[O-]	-64.929	ZINC14020223	c1cocc1C(=O)OCC(=O)c2cc([nH]c2)C(=O)N3CCCC3	-74.525
84	ZINC06750295	c1cc2c(nc1)SCCN2C(=O)CCC(=O)[O-]	-64.891	ZINC32511762	Cc1ccc2c(c1)c(c[nH]2)c3cnc(c(n3)N4CCCC(C4)C#N)N	-74.500
85	ZINC21984102	c1coc2c1cc3c(c2OCC(=O)[O-])OC(=O)CC3	-64.875	ZINC04772201	c1ccc(cc1)CSc2c3cc4c(cc3ncn2)nc[nH]4	-74.458
86	ZINC06751468	Cc1c2c(c3ccccc3s2)c(=O)n(n1)CC(=O)[O-]	-64.846	ZINC31809161	c1ccc(cc1)CN(Cc2ccccc2)c3c4cn[nH]c4ncn3	-74.456
87	ZINC05313446	CCCOc1ccc(cc1)Cc2[nH]c(=O)cc(n2)C	-64.727	ZINC12148420	c1ccc2c(c1)CCN2CCNC(=O)COc3c4ccccc4ncn3	-74.408
88	ZINC05811919	c1cc(cc(c1)C(=O)N)C2C(C(C(O2)CO)O)O	-64.664	ZINC29585225	Cc1c(nc2ccccc2n1)NCc3cccc(c3)Cn4ccnc4	-74.385
89	ZINC05424071	CCN(CC)C(=O)C1CCc2c(c(nc(n2)N)N)C1	-64.591	ZINC09109524	CCOC(=O)c1cnc(nc1C)NC2Nc3cc(ccc3C(=N2)C)C	-74.370
90	ZINC16951479	CSc1c(nnc(n1)O)Sc2ccccc2	-64.376	ZINC23192459	c1ccc2c(c1)cc(o2)c3c(c[nH]n3)CN4CCc5c(ccs5)C4	-74.366
91	ZINC26964118	Cc1nc(no1)CSc2c3c(ccs3)ncn2	-64.296	ZINC31820917	CC(C)c1nnc2n1nc(s2)CCc3nc(no3)c4ccccn4	-74.345
92	ZINC05575416	CCOC(=O)C1(C(=O)N(c2cncnc2N1)C)O	-64.279	ZINC05332107	c1ccc(cc1)c2cn(nn2)CCc3ccncc3	-74.345
93	ZINC05844289	CCCc1c(c(c(o1)CCC(=O)[O-])C(=O)[O-])C	-64.273	ZINC15181452	c1ccc2c(c1)c(n[nH]2)C(=O)OCCCn3c4ccccc4[nH]c3=O	-74.250
94	ZINC28163972	Cc1cc2c(s1)nc(nc2Sc3c([nH]nc3[O-])C)C	-64.199	ZINC19340843	c1cc(cnc1)c2nc([nH]n2)CNC(=O)c3cccnc3	-74.234
95	ZINC27751197	CSc1c(c(c2c(n1)CCCC2)C(F)(F)F)C(=O)[O-]	-64.165	ZINC26878831	c1ccc(cc1)C(c2ccccn2)NCc3nnnn3c4ccccc4	-74.192
96	ZINC12653216	Cc1c(c(nc([nH+]1)C)[NH2+]C(CO)C(=O)[O-])CC=C	-64.136	ZINC14805447	c1ccc(cc1)n2cnc(n2)SCc3nc(nc(n3)N)N	-74.191
97	ZINC12395274	Cc1c2c(ccnc2n(n1)CC(=O)[O-])c3ccco3	-63.791	ZINC20135628	Cc1c2c(nc(n1)CNc3nccc(n3)c4ncnn4C)CCCC2	-74.169
98	ZINC10119632	C=Cn1nc(nn1)NS(=O)(=O)c2ccccc2	-63.788	ZINC20862434	Cn1c(cc(n1)c2ccccn2)NC(=O)c3c4ccccc4[nH]n3	-74.158
99	ZINC04928164	c1ccc(c(c1)CNC(=O)Cc2[n-]nnn2)Cl	-63.710	ZINC09548646	c1ccc(cc1)n2c(nnn2)CN3CCCSc4c3cccc4	-74.133
100	ZINC34049542	c1c(c(cc(c1O)S(=O)(=O)[O-])S(=O)(=O)[O-])O	-63.701	ZINC23232170	c1cc(c(nc1)N2CCc3c(c(n[nH]3)C4CCC4)C2)C(=O)NC5CC5	-74.107

Rank	FolC1			FolC2		
	ZINC ID	Smiles	Score	ZINC ID	Smiles	Score
1	ZINC20513436	COCCc1nc(on1)c2ccccc2n3cccn3	-74.776	ZINC30793724	c1ccc2c(c1)nc3n2CCN3C(=O)Cn4c5ccccc5oc4=O	-106.852
2	ZINC20869430	CCC(c1ccccc1)c2c3c([nH]n2)CCN(C3)Cc4ccccn4	-73.044	ZINC20205182	c1ccc(cc1)C2(CC2)c3nc(on3)CCc4c[nH]nc4	-101.490
3	ZINC17885173	c1ccc2c(c1)ccc(n2)Cc3c(=O)[nH]c4ccccc4n3	-72.454	ZINC14768202	c1ccc2c(c1)c(c[nH]2)c3c[nH]c(n3)C(=O)c4c[nH]c5c4cccc5	-101.006
4	ZINC22521194	Cc1cc(no1)COc2ccccc2OCCN3CCOCC3	-69.877	ZINC19799817	CC(C)c1nc(on1)C2CCCN2C(=O)CCc3cccnc3	-100.614
5	ZINC25626217	c1cc(oc1)c2nc(cs2)Cn3cnc(n3)C#N	-69.639	ZINC32997889	c1ccc2c(c1)c(c[nH]2)CCC(=O)N3CCSc4ccccc4	-97.947
6	ZINC19112326	Cc1cc([nH]n1)CNC(=O)c2ccccc2c3[nH]ncn3	-69.074	ZINC16976456	CCOC(=O)c1ccc(cc1)NCc2nc3c(nc(nc3O)N)nn2	-97.942
7	ZINC32565278	Cc1nnn(n1)C23CC4CC(C2)CC(C4)(C3)Nc5cnccn5	-68.979	ZINC14123481	Cc1ccc2c(c1)occ2CC(=O)N3CC(=O)Nc4c3cccc4	-97.937
8	ZINC16524996	c1ccc2c(c1)OCC(O2)CSc3cccc4c3nccc4	-68.830	ZINC23339519	Cc1c(c(on1)C)c2c3c([nH]n2)CCN(C3)c4nc5ccccc5o4	-97.898
9	ZINC14986585	Cc1cc2c(c(c1)c3ccccn3)OC(C2)CNC(=O)C	-68.748	ZINC15225635	c1cc2c(cc1Oc3ccc4c(c3)nc(c(n4)O)O)nc(c(n2)O)O	-97.808
10	ZINC20272664	c1ccc(c(c1)NC2CCOCC2)OCc3ccccn3	-68.476	ZINC13212165	c1cc(ccc1CSc2c(nc3c(n2)c(nc(n3)N)N)N)Cl	-97.603
11	ZINC11049269	c1ccc2c(c1)CCN2Cc3csc(n3)c4ccccn4	-68.073	ZINC09668773	Cc1ccc(cc1)NC(=O)C2CC(=O)Nc3n2c4ccccc4n3	-97.503
12	ZINC29403079	Cc1nc(cs1)c2ccc(o2)CNc3nc4ccccc4o3	-68.027	ZINC20286408	COc1ccc2cc3c(nn(c3nc2c1)C(=O)CN4CCOCC4)N	-97.035
13	ZINC04770233	C=CC1(Cc2ccccc2C1)c3c[nH]cn3	-67.964	ZINC23399445	c1ccc(cc1)C=Cc2c3c([nH]n2)CCN(C3)c4ncc(c(n4)N)F	-96.871
14	ZINC20205182	c1ccc(cc1)C2(CC2)c3nc(on3)CCc4c[nH]nc4	-67.779	ZINC24978409	CCCCCn1ccc(=NC(=O)c2ccccc2C(=O)[O-])cc1	-96.735
15	ZINC15020395	c1ccc(cc1)Oc2cccc3c2c([nH][nH+]3)N	-67.661	ZINC12576364	c1ccc(cc1)C(=O)CC(c2[nH]ccn2)C(=O)c3ccccc3	-96.673
16	ZINC17148483	Cc1ccc(cc1)Sc2c3c([nH]cn3)ncn2	-67.336	ZINC20997929	CCc1nc(on1)CN(C(C)C(=O)c2ccccc2c3[nH]ncn3	-96.347
17	ZINC20917089	Cc1c(c(on1)C)CCN2CCCC2c3[nH]c4ccc(cc4n3)F	-67.267	ZINC19898699	c1ccnc(c1)NC(c2ccc3cccnc3c2[O-])c4ccco4	-96.337
18	ZINC32551051	Cc1c(nccn1)c2cccc(c2C(=O)N(C(C)C)C(C)C)OC	-67.250	ZINC20256480	Cc1c(cccn1)c2nc(no2)C3(CCOC3)c4ccc(cc4)F	-96.305
19	ZINC19938873	Cc1cc(n(n1)C)CN2CCc3c(c([nH]n3)c4cc(on4)C)C2	-66.985	ZINC16583070	c1ccc2c(c1)c(=O)n(cn2)Cn3c(=O)c4ccccc4nn3	-96.070
20	ZINC12545208	Cc1cc(on1)COC(=O)c2ccccc2NCc3ccco3	-66.754	ZINC19938818	c1ccc(cc1)C2CCCN(CC2)C(=O)c3c[nH]c(=O)[nH]c3=O	-96.069
21	ZINC12545628	Cc1c(nc2ccccc2n1)CSc3ccccn3	-66.737	ZINC20601465	c1ccnc(c1)c2nc(on2)CN3CCc4c(cn[nH]4)C3	-95.889
22	ZINC22015370	CCC(C#N)(c1ccccc1)c2ccccn2	-66.614	ZINC32923591	c1ccc2c(c1)nc(o2)C3CCCN(C3)c4ccc5nnnn5n4	-95.825
23	ZINC32541007	c1ccc(cc1)Oc2ccccc2c3cnc(cn3)N	-66.603	ZINC31147705	Cc1c(c2ccccc2[nH]1)CC(=O)OCc3nnnn3c4ccccc4	-95.792
24	ZINC26102943	CCc1nnc(o1)CN2Cc3ccccc3C(C2)c4ccccc4	-66.592	ZINC23310836	c1ccn2c(c1)ncc2CC(=O)N3CCc4c(c(n[nH]4)C5CCC5)C3	-95.559
25	ZINC12421660	c1ccc(c(c1)Nc2cc(nc(n2)N)Cl)F	-66.366	ZINC12902053	Cc1cc(no1)NC(=O)COC(=O)c2ccc(nc2)n3cncn3	-95.410
26	ZINC27595943	c1ccc(cc1)OC(=O)C2CCCN(C2)c3ncccn3	-66.351	ZINC23399546	Cc1c(c(on1)C)c2c3c([nH]n2)CCN(C3)c4c(nccn4)C#N	-95.188
27	ZINC19781559	c1cc(c2cc[nH]c2c1)c3nc(on3)CC4CCC(=O)N4	-66.257	ZINC20821591	Cc1nc2cc(ccc2o1)c3cccc(c3)c4ccn(n4)CCCO	-95.045
28	ZINC20864533	Cc1ccccc1c2nc(c(o2)C)CN3c4ccnn4CCC3=O	-66.176	ZINC13283769	Cc1nc(no1)c2ccc3ccc4cccnc4c3n2	-94.957
29	ZINC32561862	COc1ccc2c(c1)NC(=O)C2Cc3nc(no3)c4ccccn4	-66.140	ZINC06712858	COCCNC(=O)c1ccc2c(c1)[nH]c-3c4ccccc4nc3n2	-94.940
30	ZINC19403455	c1ccnc(c1)c2nnc(o2)CNC(=O)C3CCCC3	-66.100	ZINC12239258	CC1=CC2=NC(=O)CC(N2C=C1)C(C(=O)N3CCC3(CC=C)CC=C	-94.712
31	ZINC08048411	c1ccc(cc1)CCc2nc([nH]n2)c3ccccn3	-66.060	ZINC09479045	c1ccc(cc1)n2c(=O)n(nn2)CCCn3c4ccccc4[nH]c3=O	-94.662
32	ZINC12939885	c1ccc(cc1)C(Cc2ccccc2C#N)(c3ccccn3)O	-65.879	ZINC12574652	COc1ncc(cn1)C(=O)N2CC3(C2)CCCN(C3=O)CC4CC4	-94.626
33	ZINC32529853	c1cc(sc1)Cc2nc(on2)CCc3c[nH]nc3	-65.707	ZINC23144257	CN1C(Nc2c(ccc(n2)c3ccs3)C1=O)C(=O)NCCOC	-94.503
34	ZINC32751344	CN(CCOc1ccccc1)Cc2nc(nc(n2)N)N	-65.702	ZINC19145978	Cc1[nH]c2ccc(ccc2n1)NCC3(CCOC3)c4ccc(cc4)F	-94.482
35	ZINC20601743	c1ccc(c(c1)F)CC2CN(CCO2)Cc3nccs3	-65.659	ZINC30811049	Cc1cc(n2c(n1)c(cn2)C(=O)OCC(=O)Nc3nc(cs3)C)C	-94.361
36	ZINC19740391	c1ccc(cc1)Oc2c(ccn2)c3nc(on3)C4CCCO4	-65.545	ZINC20208336	COc1ccc(cc1)CCN(CC2CCCO2)C(=O)c3cnc[nH]3	-94.338
37	ZINC20509329	c1ccc(cc1)c2c(c(=O)c2=O)NCc3ccccn3	-65.540	ZINC12023651	CC(=O)NCc1cn(nc1c2ccccc2)c3ccccc3	-94.213
38	ZINC05753699	c1c(cnc(c1Cl)SCc2[nH]nc(n2)CC#N)C(F)(F)F	-65.507	ZINC19744740	c1cc(cc(c1)F)c2c3c([nH]n2)CCN(C3)c4ccnc5c4cn[nH]5	-94.188
39	ZINC19452874	CC(C)c1cc(on1)C(=O)N2CCCC(C2)c3cnccn3	-65.436	ZINC30773513	c1cc(c(nc1)N2CCC(CC2)C(=O)c3ccc4c(c3)OCCO4)C#N	-94.004
40	ZINC32576679	Cc1nc(cs1)C(=O)N2CCc3c(c(n[nH]3)C(C)C)C2	-65.225	ZINC08343112	CC(C)n1c(=[NH+]C(=O)c2ccc(=O)n(n2)C)cc[nH]1	-93.971
41	ZINC28914348	c1ccc2c(c1)OCC(O2)CNS(=O)(=O)c3ccc(cc3)F	-65.140	ZINC32911897	Cn1cnnc1C2CCCN(C2)S(=O)(=O)c3ccc(cc3)C#N	-93.922
42	ZINC22988228	CCc1nc(cs1)COC(=O)c2cn3c(cccc3n2)C	-65.087	ZINC32825021	c1cnn(c1)c2cc(ccn2)CNc3ccc4nnnn4n3	-93.804
43	ZINC20864587	c1ccc(c(c1)c2[nH]ccn2)c3nc(no3)c4ccncc4	-65.031	ZINC05808496	CN1CCc2ccccc2C(C1=O)C(=O)c3ccc4c(c3)OCO4	-93.773
44	ZINC06309687	c1ccc2c(c1)CCN2c3c4c([nH]cn4)ncn3	-65.009	ZINC25764343	CCN(CC)S(=O)(=O)c1ccc(nc1)N2CCc3ccccc3C2	-93.747
45	ZINC20773986	Cc1cc(=O)c(c[nH]1)C(=O)N2CCCCC2c3ccccn3	-64.891	ZINC12894834	c1ccc2c(c1)c(c[nH]2)CCn3cc(ccc3=O)C(=O)[O-]	-93.721
46	ZINC35528029	c1ccc(cc1)n2ccc(=O)c(n2)c3nc(no3)c4ccccn4	-64.857	ZINC04810508	c1ccc(cc1)N(Cc2cc3ccccc3[nH]c2=O)C(=O)c4nc[nH]n4	-93.694
47	ZINC21367648	CCCN1Cc2cccc(c2C1=O)c3nc(c(o3)C(=O)OCC)C	-64.836	ZINC20586874	Cc1ccc(n1)C(F)(F)F)c2c3c([nH]n2)CCN(C3)CC4CC4	-93.682
48	ZINC29209298	CN(C1CCc2c1cccc2)C(=O)c3ccccn3	-64.730	ZINC20255965	COc1cccc(c1)CN2CCN(CC2=O)C(=O)c3cncnc3	-93.669
49	ZINC20086532	Cc1c2c([nH]n1)NC3=C(C2c4c(nc5n4ccs5)C)C(=O)OC3	-64.720	ZINC23580732	Cc1nccc(n1)N2CCc3c(c(n[nH]3)CSc4ccccc4)C2	-93.649
50	ZINC32922935	c1ccc(c(c1)Cn2cccn2)NC(=O)c3ccccn3	-64.673	ZINC31616206	COCCn1c(=O)c2cccnc2nc1SCc3ccccc3F	-93.635
51	ZINC32937984	Cc1ccccnc1N2C(=O)CN=C2c3ccc(o3)Br	-64.658	ZINC32511762	Cc1ccc2c(c1)c(c[nH]2)c3cnc(c(n3)N4CCCC(C4)C#N)N	-93.509
52	ZINC26730324	c1ccc(cc1)OCCNc2cnc3ccccc3n2	-64.577	ZINC23214524	CC1Cc2cc(ccc2O1)C(=O)N3CC(CC3)c4cc(=O)[nH]cn4	-93.462
53	ZINC05263201	c1ccnc(c1)SCc2[nH]c(=O)c3c(n2)ccs3	-64.562	ZINC24992248	c1ccc(cc1)n2cc(cn2)COC(=O)Cc3c[nH]c4c3cccc4	-93.279
54	ZINC22145508	c1ccc(cc1)C(CN2CCOCC2)(c3ccccn3)O	-64.484	ZINC06659107	Cc1c(c2cc(ccc2o1)NS(=O)(=O)c3ccc(s3)C(=O)[O-]	-93.014
55	ZINC20907968	c1ccnc(c1)c2nc(on2)CCNC(=O)C3CCOC3	-64.480	ZINC23187563	c1ccc2c(c1)C(=O)N(C2(CC(=O)[O-])O)CCCN3CCOCC3	-92.965
56	ZINC05004551	c1ccc(cc1)C2=NC(=O)C(O2)C(=O)c3ccccc3	-64.476	ZINC32829159	c1ccc(c(c1)COC(=O)CCn2c3ccccc3c(=O)cn2)C#N	-92.929
57	ZINC17527075	c1ccc2c(c1)c(c[nH]2)CC(=O)OCc3nc(nc(n3)N)N	-64.379	ZINC19222149	CN(CCc1ccccc1)Cc2nc(no2)c3cnccn3	-92.850
58	ZINC32519387	COc1cccc(c1)c2cc(ncn2)n3cccn3	-64.336	ZINC32601131	c1ccc(cc1)SC(CN2CCOCC2)n3c4ccccc4nn3	-92.839
59	ZINC32533054	CN(CCc1ccccn1)C(=O)c2ccccc2c3[nH]ncn3	-64.272	ZINC23447612	Cc1ccc(cc1)SCC(=O)c2cc([nH]c2C)C	-92.726
60	ZINC32771221	Cc1c(oc(n1)Sc2c(nc3ccccc3n2)C)C	-64.259	ZINC20993055	CC(C)c1nc(on1)CC2(C(=O)c3ccccc3C2=O)c4ccccc4	-92.713
61	ZINC32577787	COc1c(cccn1)c2cc(ncn2)n3ccn3	-64.171	ZINC23106269	c1cc2c(cc1C(=O)CSc3ncc(c(n3)N)C#N)CCO2	-92.642
62	ZINC13229996	Cn1c2ccccc2nc1Cc3nc4ccccc4n3C	-64.123	ZINC04962987	CCOC(=O)c1c[nH]c2ccc(nc2c1=O)Oc3ccc(cc3)F	-92.588
63	ZINC24993415	Cn1cnnc1SCC(Cn2c3ccccc3c4c2cccc4)O	-64.115	ZINC23580732	Cc1nccc(n1)N2CCc3c(c(n[nH]3)CSc4ccccc4)C2	-92.563
64	ZINC13214358	CC(=O)NC1CCCC(=Cc2ccnc3c2cc(cc3)OC)C1	-64.096	ZINC23214420	CCn1cc(nn1)NC(=O)Cn2cc(cn2)c3cnn4c3nccc4	-92.539
65	ZINC31776379	CC(C)Oc1ccccc1NC(=O)c2cnccn2	-64.075	ZINC29808772	c1ccc2c(c1)cnn(c2=O)CC(=O)Nn3cnc4c3cccc4	-92.527
66	ZINC19223398	Cc1c(non1)CN(C(C)C(=O)c2ccccc2c3[nH]ccn3	-64.075	ZINC06590515	c1ccc2c(c1)cc(o2)C(=O)C(C#N)c3[nH]c4ccccc4n3	-92.519
67	ZINC12421659	COc1ccccc1Nc2cc(nc(n2)N)Cl	-64.068	ZINC19801234	c1ccc2c(c1)c(=O)c(c(o2)[O-])Sc3ccccc3C(=O)[O-]	-92.479
68	ZINC14608397	c1ccnc(c1)SCc2cc(=O)n3c(n2)SCC3	-64.047	ZINC17972412	c1ccc2c(c1)cc(o2)C(=O)CSc3[nH]ncn3	-92.431
69	ZINC12118229	Cc1nccc(n1)c2cccc(c2)NCc3cccs3	-63.965	ZINC05733839	c1ccc2c(c1)C(OC2=O)Cc3c(cccn3)C(=O)[O-]	-92.335
70	ZINC18054883	c1ccc(c1)c2c3ccccc3nc(n2)NCCCO	-63.951	ZINC19813117	c1ccc(cc1)COC2CCCN(C2)C(=O)Cc3c[nH]nc3	-92.332
71	ZINC19403993	c1cnc(nc1)c2nc(on2)C3CN(CCO3)C(=O)C4CCCC4	-63.930	ZINC21266215	c1ccc2c(c1)cnn2CC(c3ccc(cc3)F)(c4ccccn4)O	-92.330
72	ZINC32551079	c1cnc(cn1)NC23CC4CC(C2)CC(C4)(C3)n5cncn5	-63.923	ZINC19782292	c1cc(sc1)c2c3c([nH]n2)CCN(C3)Cc4cnc5nccn5c4	-92.298
73	ZINC20996927	CN(Cc1[nH]c2ccccc2n1)Cc3nc(no3)CC4CC4	-63.883	ZINC23466233	CCC(c1ccnn1C)Nc2nccc(n2)c3cnn(c3)C	-92.289
74	ZINC32513781	Cc1cccc2n1c(nn2)c3ccccc3Cn4cccn4	-63.745	ZINC23399445	c1ccc(cc1)C=Cc2c3c([nH]n2)CCN(C3)c4ncc(c(n4)N)F	-92.240
75	ZINC24247088	CCC(=O)NCc1ccc(o1)c2csc(n2)C	-63.667	ZINC19113452	Cc1ccc(nc1)N2CCC(C2)C3CCN(CC3)c4ncccn4	-92.147
76	ZINC09421229	c1ccc2c(c1)[nH]c(n2)CCc3nc(no3)c4ccccn4	-63.636	ZINC11339061	c1ccc(cc1)n2c(nnn2)COC(=O)c3c(nccn3)N	-92.103
77	ZINC06549070	c1ccnc(c1)Nc2nc(cs2)c3ccncc3	-63.635	ZINC16844102	Cc1ccc(cc1)CSc2n[nH]c(=O)c3n2nc(c3)c4ccco4	-92.103
78	ZINC32541379	c1ccc(c(c1)CNc2nccn2)n3cccn3	-63.622	ZINC12788324	c1cc(ccc1NC(=O)Cc2[nH]c(=O)c3ccc(cc3n2)C(=O)[O-])F	-92.093
79	ZINC22317383	Cc1cc(n(n1)CCC(=O)N=c2ccccn2Cc3ccccc3)C	-63.550	ZINC20859279	c1ccc(cc1)c2c(c[nH]n2)CN3CCCC(C3)c4ccn[nH]4	-92.091
80	ZINC12475431	c1ccnc(c1)c2nc(cs2)CSc3ccc4c(c3)OCCO4	-63.508	ZINC32918541	c1ccc(c1)c2cc3n(c2)C(CNC3=O)CC(=O)[O-])F	-92.053
81	ZINC15109455	c1ccc(cc1)CN(c2ccccn2)c3ccccn3	-63.504	ZINC32576561	CCn1c(c(c(n1)C)CCn2cnc3c(c2=O)cccn3)C	-92.009
82	ZINC32587265	CC(C)N(C(C)C)C(=O)c1cccc1c2nccn2	-63.389	ZINC23128396	Cc1nc(c(n1)N2CCc3c(c(n[nH]3)C4CCCC4)C2)n5cccn5	-91.961
83	ZINC23231364	Cc1c(c(on1)C)Cc2cc(n3c(n2)c(cn3)c4ccccn4)[O-]	-63.321	ZINC23310809	c1c(ncnc1N2CCc3c(c(n[nH]3)C4CCCC4)C2)NCCO	-91.919
84	ZINC31820917	CC(C)c1nnc2n1nc(s2)CCc3nc(no3)c4ccccn4	-63.282	ZINC12873917	c1nc(s1)CC(=O)N)C23CC4CC(C2)CC(C4)C3	-91.862
85	ZINC16847028	c1ccc(cc1)c2c(c[nH]n2)c3nc(no3)c4ccccn4	-63.263	ZINC19558926	COc1ccc(cc1)n2c3c(cn2)CN(CCC3)C(=O)c4cnccn4	-91.697
86	ZINC09142708	c1ccc2c(c1)C(=O)NC(O2)CCc3ccccn3	-63.242	ZINC32814917	c1ccc(cc1)n2ncc(n2)C(=O)NCc3nc(no3)c4ccccn4	-91.667
87	ZINC31785599	Cc1cc(no1)NC(=O)CNc2cccc(c2)c3cnc(n3)C	-63.242	ZINC23128396	Cc1nc(c(n1)N2CCc3c(c(n[nH]3)C4CCCC4)C2)n5cccn5	-91.641
88	ZINC33373941	c1ccc(cc1)CN2Cc3ccccc3C(C2)F	-63.203	ZINC04939931	COc1ccc2cc3c(nn(c3nc2c1)C(=O)c4ccco4)N	-91.633
89	ZINC32511854	Cc1csc(n1)CN(C)c2c3cn[nH]c3nc(n2)N	-63.170	ZINC32006099	c1ccc2c(c1)CC(O2)CNC(=O)C3CC(=O)Nc4c3cccc4	-91.609
90	ZINC32103121	CC(C)c1nnc2n1nc(s2)Cc3csc(n3)c4ccccn4	-63.133	ZINC19339616	c1cc(cnc1)CN2CC(C(C2)c3cc[nH]n3)c4ccc(cc4)F	-91.567
91	ZINC20996044	CN(CCn1ccnc1)Cc2[nH]c3ccc(cc3n2)Cl	-62.993	ZINC06486110	c1cnc(cn1)C(=O)OCC(=O)C23CC4CC(C2)CC(C4)C3	-91.512
92	ZINC32825889	Cc1nc(cs1)Cc2nc(cs2)c3ccccc3OC	-62.968	ZINC27444096	CCCn1c(nnn1)COC(=O)C2CC(=O)N(C2)c3ccc(cc3)C	-91.493
93	ZINC26420718	c1cc(ccc1c2cc(ncn2)Cl)F	-62.918	ZINC17527075	c1ccc2c(c1)c(c[nH]2)CC(=O)OCc3nc(nc(n3)N)N	-91.473
94	ZINC05331959	c1ccc(cc1)C(Cc2ccccn2)(C#N)c3ncccn3	-62.904	ZINC04705544	c1ccc(cc1)CCn2c(=O)c(c([nH]c2=O)O)Cc3cccs3	-91.442
95	ZINC29964301	CN(Cc1nc(no1)c2cccco2)Cc3nc4ccccc4s3	-62.869	ZINC31798878	Cc1nc(cs1)Cn2c(ccc(c2=O)C(=O)N)C(F)(F)F	-91.420
96	ZINC27227705	CC(c1cc2ccccc2o1)N(C)Cc3nnc(o3)C(C)(C)C	-62.836	ZINC19656689	Cc1c(non1)CN(C)Cc2c[nH]nc2c3ccc(cc3)Cl	-91.405
97	ZINC20201393	Cc1ccn(n1)CCc2nc(no2)Cc3ccsc3	-62.814	ZINC16650957	CCCc1c(cc(o1)c2ccccc2)C(=O)N3CCOCC3	-91.387
98	ZINC09365427	c1cc(sc1)c2nc(cs2)c3ccc(cc3O)O	-62.803	ZINC21157244	Cc1ccc(cc1S(=O)(=O)CCC(=O)[O-])c2ccc(=O)[nH]n2	-91.321
99	ZINC22124280	c1ccc(c(c1)C#N)c2ccc(o2)C#N	-62.780	ZINC12792251	c1ccc2c(c1)nc(s2)c3ccc(o3)CNc4ccc5nnnn5n4	-91.230
100	ZINC10973809	CCN(CC)C(=O)CN(C)c1cc(=O)n2ccccc2n1	-62.749	ZINC11559613	c1ccc(cc1)n2cc(cn2)CNC(=O)c3cc4ccccc4oc3=O	-91.171

	HGPRT			TYLT		
Rank	ZINC ID	Smiles	Score	ZINC ID	Smiles	Score
1	ZINC19940113	Cc1nnc(o1)Cn2ccc(n2)c3cccc(c3)c4ccccn4	-63.715	ZINC14240853	c1ccc2c(c1)c(n[nH]c2=O)CC(=O)Nn3cnc4c3cccc4	-75.985
2	ZINC09114820	CC(C)n1c(=[NH+]C(=O)CSc2ccc3ccccc3n2)cc[nH]1	-59.407	ZINC32741542	c1cc2cccc3c2c(c1)N(S3(=O)=O)CCSc4nc([nH]n4)N	-74.496
3	ZINC26895861	c1ccc(cc1)COC(=O)NC(CC(=O)[O-])c2c[nH]c3c2cccc3	-58.817	ZINC08747729	c1cc(sc1)C(=O)OCC(=O)c2ccc3c(c2)[nH]c(=O)[nH]3	-74.100
4	ZINC27660342	c1ccc(cc1)c2c[nH]c(n2)SCc3nc(nc(n3)N)N	-58.817	ZINC20776501	c1ccc(c(c1)C(=O)N2CCCOCC2)NCc3ccccn3	-73.548
5	ZINC17174208	c1cc(ccc1C(=O)[O-])NCc2c3c([nH]cn3)ncn2	-58.667	ZINC08748078	c1cc(oc1)C(=O)OCC(=O)c2ccc3c(c2)[nH]c(=O)[nH]3	-72.802
6	ZINC16887153	Cc1cc(nc(n1)Oc2ccc(nn2)O)Oc3ccccc3	-58.501	ZINC19124373	Cc1cc(on1)c2cncnc2c3ccc(nc3)N4CCOCC4	-72.289
7	ZINC14187761	c1cc2c(cc1Br)c(=O)[nH]c(n2)c3[nH]cnn3	-57.894	ZINC28514424	CC(C(=O)Nc1cccc2c1[nH]nc2)Nc3nc4ccccc4s3	-72.169
8	ZINC19782366	Cc1cc(n(n1)c2cccc(c2)CN(C)C(=O)Cc3[n-]nnn3)C	-57.846	ZINC23337581	CCc1c2c([nH]n1)CCN(C2)Cc3cnc(nc3)c4ccccn4	-71.194
9	ZINC24792966	Cc1cccc(n1)C(=O)NCCCc2cc(n(n2)c3ccccc3)N	-57.071	ZINC23479179	Cc1c(c2ccccc2[nH]1)CCNC(=O)Nc3[n-]nnn3	-71.157
10	ZINC16383615	c1c2c(cc3c1nc(c(n3)C(=O)[O-])C(=O)[O-])[nH]c(=O)[nH]2	-56.897	ZINC11330441	c1cc(ccc1C2CC(=O)N=C3C2=C([NH+]=C(N3)N)[O-])O	-71.033
11	ZINC12139034	c1cnc(nc1SCC(=O)[O-])SCC(=O)[O-]	-56.731	ZINC23398636	Cn1c(ccn1)C(=O)N2CCc3c(c(n[nH]3)CCc4ccccc4)C2	-70.936
12	ZINC26276285	c1cc(=O)[nH]nc1C(=O)Nc2[n-]c(c(n2)C#N)C#N	-56.517	ZINC04626582	c1ccc(c(c1)C2c3c([nH]nc3C(=O)N2)c4ccco4)F	-70.772
13	ZINC05386138	CCn1cc(c(=O)c2c1nc(cc2)c3c[nH]nc3C)C(=O)[O-]	-56.172	ZINC19722847	c1ccc(cc1)c2c(cn[nH]2)CN3CCc4ccccc4C3	-70.755
14	ZINC11903678	CCCOC(=O)c1ccc(cc1)CNc2cn[nH]c(=O)n2	-56.130	ZINC33413876	c1cc(ccc1CNc2ccc3c(n2)[nH]c(=O)[nH]3)F	-70.715
15	ZINC21964365	c1c([nH]cn1)CC(C(=O)[O-])NC(=O)C2CCCO2	-56.117	ZINC13212409	c1nc2c(n1COC(CO)CO)c(=O)[nH]c(n2)N	-70.629
16	ZINC30691545	c1ccn2c(c1)nnc2NC(=O)c3cc([nH]n3)C4CC4	-55.920	ZINC05754749	CC(C#N)OC(=O)Cc1c2ccccc2c(=O)[nH]n1	-70.300
17	ZINC08919859	c1ccc(cc1)Cc2nnc(o2)NC(=O)c3ccco3	-55.851	ZINC32587099	Cc1ncc(cn1)CNc2ccc(cc2)N3CCOCC3	-70.017
18	ZINC32276521	c1ccc(cc1)c2nnn(n2)CC(=O)NCCc3[nH]c4ccccc4n3	-55.790	ZINC19703144	Cc1ccc2c(c1)c(nc(n2)NC3=NCC(=O)N3)C	-69.918
19	ZINC04963604	c1cc(ccc1C(=O)[O-])NCC(=O)Nc2c(nc(nc2N)N)N	-55.686	ZINC07166489	Cc1c(sc2c1c(ncn2)OCC(=O)N3CCc4c3cccc4)C	-69.815
20	ZINC12308107	Cc1ccc(o1)c2cnnc(n2)NCCN3CCCc4c3cccc4	-55.589	ZINC15879123	Cc1c(c(=O)[nH]c(=O)[nH]1)S(=O)(=O)N2CCCc3c2cccc3	-69.793
21	ZINC14315072	c1ccc(cc1)n2cc(cn2)CNc3cn[nH]c(=O)c3Cl	-55.539	ZINC35683253	c1cc(cnc1)c2cc(=O)n3c(n2)C(CCC3)NC(=O)c4ccco4	-69.731
22	ZINC12788378	COC(=O)c1ccc2c(c1)nc([nH]c2=O)c3[nH]cnn3	-55.439	ZINC32547721	Cc1cccc2c1c(=O)n(cn2)CCNc3[nH]c(=O)cc(n3)C	-69.559
23	ZINC12433971	c1cc(cc(c1)F)c2cnnc(n2)N3CCc4c(cn[nH]4)C3	-55.188	ZINC12538515	c1ccc2c(c1)c(n[nH]c2=O)C(=O)NCCCc3c(c([nH]n3)N)C#N	-69.464
24	ZINC13215634	c1ccc(cc1)c2c(c(nc(n2)N)N)NCCCc3ccc(cc3)N	-54.964	ZINC16044906	Cc1c2cc(sc2n(n1)Cc3ccccc3)C(=O)N=c4[n-][nH]nn4	-69.464
25	ZINC21157244	Cc1ccc(cc1S(=O)(=O)CCC(=O)[O-])c2ccc(=O)[nH]n2	-54.877	ZINC19112326	Cc1cc([nH]n1)CNC(=O)c2ccccc2c3[nH]ncn3	-69.461
26	ZINC12429913	c1ccc(cc1)c2cnnc(n2)NCCc3nc(no3)c4cccnc4	-54.510	ZINC14983264	Cc1cn[nH]c1C2CCCN(C2)C(=O)c3cccc(c3)Cn4cccn4	-69.252
27	ZINC08973822	c1ccc2c(c1)NC3=NC(=[NH+]C(N23)c4cccnc4)N	-54.314	ZINC11866515	CCCc1cc(=O)oc2c1cc(c(c2C)O)c3ccc(c(=O)[nH]3)C#N	-69.212
28	ZINC23143328	c1ccc2c(c1)[nH]c(n2)SCc3ccc(cc3)C(=O)Nc4[n-]cnn4	-54.291	ZINC20782647	Cc1cc(nc(n1)SCC(=O)NCc2nc3c(s2)CCCC3)[O-]	-69.118
29	ZINC09111405	c1ccc(cc1)C2NC(=[NH2+])Nc3n2c4ccccc4n3	-54.061	ZINC20869223	c1cc(sc1)c2ccc(o2)CCC(=O)NCc3ccncn3	-69.055
30	ZINC23310283	Cc1c(non1)CC(=O)N2CCc3c(c(n[nH]3)C4CCC4)C2	-53.956	ZINC05799573	CCOC(=O)c1c2[nH]nc-3ccc(=O)cc3n2cn1	-69.034
31	ZINC19811667	c1ccc(c(c1)C(=O)NCCNC(=O)c2cnc[nH]2)Cl	-53.921	ZINC05687294	Cc1ccccc1Cc2c(c3c(n[nH]c3oc2=O)C)C	-68.866
32	ZINC07657512	c1ccc(cc1)OC(=O)c2ccc3c(c2)[nH]c(=O)c(=O)[nH]3	-53.871	ZINC09415873	c1csc2c1c(nc(n2)Sc3c4c(ccs4)ncn3)N	-68.777
33	ZINC27670069	CCCn1c(nnn1)CSc2nc3ccccc3c(n2)NC	-53.704	ZINC12147748	c1cc(cc(c1)NS(=O)(=O)c2[nH]ncn2)c3ccc(cc3)F	-68.700
34	ZINC35238162	CS(=O)(=O)NCCNc1c2c([nH]cn2)ncn1	-53.663	ZINC16948710	CC(=O)NC(CSNN=C1C(=[NH+]C=N1)C(=O)N)C(=O)[O-]	-68.660
35	ZINC11664508	Cc1ccc(o1)c2cnnc(n2)NCc3c4c([nH]n3)CCCCC4	-53.641	ZINC12900845	c1ccc(c(c1)C(=O)c2cc3cc4c(cc3nc2)[nH]c(=O)[nH]4)O	-68.632
36	ZINC08582280	c1cc(ccc1C(=O)[O-])NCCC2CNc3c(c(nc(n3)N)O)N2	-53.589	ZINC20909167	Cc1cnc(cn1)CNC(=O)c2ccc(cc2)c3ccn[nH]3	-68.487
37	ZINC26895896	c1ccc(cc1)COc2cc3c(c[nH]c3cn2)C(=O)[O-]	-53.589	ZINC06658266	c1ccc(cc1)c2cc(c3ccccc3n2)C(=O)C4CCCNC4=O	-68.402
38	ZINC32843658	CNC(=O)NCc1ccc(cc1)CS(=O)(=O)NC	-53.524	ZINC10339450	c1ccc(cc1)C2Cc3c(cc4c(n3)[nH]nc4O)C(=O)C2	-68.370
39	ZINC27977699	c1cn(cn1)CCCNC(=O)Cn2cnnn2	-53.503	ZINC07996995	c1ccn2cc(nc2c1)CCNC(=O)c3ccc(=O)[nH]n3	-68.249
40	ZINC23368428	c1ccnc(c1)CCC(=O)N2CCc3c(c(n[nH]3)c4ccco4)C2	-53.451	ZINC20997929	CCc1nc(on1)CN(C)C(=O)c2ccccc2c3[nH]ncn3	-68.203
41	ZINC20290586	Cc1nc([nH]n1)SC(=C2c3ccccc3NC2=O)C(=O)[O-]	-53.400	ZINC25499546	c1cc(c(c(c1)F)C#N)NCc2ccnc(c2)n3cccn3	-68.162
42	ZINC22127048	Cc1c(c2c([nH]1)CC(CC2=O)c3ccco3)C(=O)[O-]	-53.365	ZINC07990466	c1cnccc1c2[nH]nc(n2)SCC(=O)C3C=CC=[NH+]3	-68.087
43	ZINC13692120	c1ccc(cc1)c2nc(cs2)CC(=O)Nc3nc[nH]n3	-53.319	ZINC07990466	c1cnccc1c2[nH]nc(n2)SCC(=O)C3C=CC=[NH+]3	-68.087
44	ZINC12508018	CCn1c2ccc(cc2nc1CSc3nc([nH]n3)N)C(=O)[O-]	-53.288	ZINC15879123	Cc1c(c(=O)[nH]c(=O)[nH]1)S(=O)(=O)N2CCCc3c2cccc3	-68.046
45	ZINC09112474	CCOC(=O)c1c(c([nH]c1C)C(=O)Nn2cnc3c2cccc3)C	-53.273	ZINC05634281	c1cc(ccc1CSc2nc(sn2)NC(=O)c3[nH]ncn3)F	-68.025
46	ZINC08994271	c1ccc(cc1)C2[NH+]=C(N=C3N2c4ccccc4C(=O)N3)N	-53.261	ZINC23232249	c1cc2c(cc1Cc3cc(n4c(n3)cc(n4)C5CCC5)[O-])NC(=O)CO2	-67.917
47	ZINC08994271	c1ccc(cc1)C2[NH+]=C(N=C3N2c4ccccc4C(=O)N3)N	-53.261	ZINC20859279	c1ccc(cc1)c2c(c[nH]n2)CN3CCCC(C3)c4ccn[nH]4	-67.867
48	ZINC05614179	c1cc(oc1)C(=O)Nc2c3ccc(cc3[nH]n2)Cl	-53.214	ZINC04717391	Cc1c(c(=O)[nH]c(=O)[nH]1)S(=O)(=O)NCc2cccs2	-67.850
49	ZINC05818833	c1c2c(cc(c1F)N3CCOCC3)nc([nH]2)SCC(=O)[O-]	-53.165	ZINC25462264	c1ccc2c(c1)nc(n2C(F)F)CSc3[nH]ncn3	-67.796
50	ZINC32743811	Cc1c(c(=O)[nH]c(n1)C)CC(=O)NC2CCSc3c2cccc3	-53.071	ZINC20997964	CN(Cc1ccon1)C(=O)c2c[nH]nc2c3cccc(c3)F	-67.739
51	ZINC16667540	c1ccc(cc1)c2cc(c3ccccc3n2)C(=O)N=c4[n-][nH]nn4	-53.068	ZINC23231889	Cc1ccc2c(c1)nc([nH]2)CNC(=O)Nc3[n-]nnn3	-67.722
52	ZINC08625683	CCCCNC(=O)Nc1cc(c[nH]c1=O)C(F)(F)F	-52.976	ZINC32092665	c1cc(cnc1)c2nc([nH]n2)NCc3ccc4c(c3)cc[nH]4	-67.623
53	ZINC17071832	c1ccc-2c(c1)CCc3c2sc(c3C(=O)N)NC(=O)CCC(=O)[O-]	-52.921	ZINC20638614	c1ccc2c(c1)CCC(C2)C(=O)NCCc3[nH]c(=O)cc(n3)N	-67.618
54	ZINC17072689	c1csc(c1C(=O)NC2CC2)NC(=O)C3C4CCC(C3C(=O)[O-])O4	-52.900	ZINC20542465	CCn1cnc(n1)NC(=O)NCC2c3ccccc3CCO2	-67.612
55	ZINC19744740	c1cc(cc(c1)F)c2c3c([nH]n2)CCN(C3)c4ccnc5c4cn[nH]5	-52.829	ZINC27004425	Cc1cc(on1)NC(=O)C(C)Nc2cccc(c2)N3CCOC3=O	-67.568
56	ZINC25044912	c1ccc(cc1)c2nc([nH]n2)Sc3ccc4nnnn4n3	-52.822	ZINC32577652	CCc1c2c([nH]n1)CCN(C2)C(=O)c3cccc(c3)c4[nH]ccn4	-67.541
57	ZINC20537602	C1CCc2c([nH]nc2CNC(=O)C3CCC=CC3)CC1	-52.726	ZINC33229838	Cc1csc(n1)CNC(=O)CCCc2ncc(o2)c3ccccc3	-67.431
58	ZINC19576876	Cc1c([nH]c2c1cc(cc2)NC(=O)c3cc([nH]c(=O)n3)CC(C)C)C	-52.603	ZINC12561276	c1ccc2c(c1)[nH]c3n2c(nn3)SCc4ccc(o4)C(=O)[O-]	-67.390
59	ZINC19744740	c1cc(cc(c1)F)c2c3c([nH]n2)CCN(C3)c4ccnc5c4cn[nH]5	-52.585	ZINC23127240	c1ccc2c(c1)CCN2C(=O)Cc3cc(on3)c4ccco4	-67.330
60	ZINC05593993	c1ccc2c(c1)Nc3cc(ccc3S2)C(C(=O)[O-])O	-52.568	ZINC32501669	c1ccc2c(c1)[nH]c(n2)SCc3[nH]c(nn3)c4ccccn4	-67.314
61	ZINC07126183	COc1ccccc1C=CC2[NH+]=C(N=C3N2c4ccccc4N3)N	-52.535	ZINC04626198	CC1(C(N(C(=O)O1)CCc2cnc[nH]2)(C)O)CCc3ccccc3	-67.308
62	ZINC06147346	c1ccc2c(c1)[nH]c(n2)C(C#N)C(=O)CSc3c(=O)[nH]c(=O)[nH]n3	-52.505	ZINC31597823	c1cc(ccc1c2ccc(o2)C(=O)NCCNc3cnccn3)Cl	-67.263
63	ZINC16638552	CCCCCOC1[NH+]=C(N=C2N1c3ccccc3N2)N	-52.450	ZINC04834785	c1ccc(cc1)c2ccc(n2NC(=O)c3ccccc3)CCC(=O)[O-]	-67.260
64	ZINC16638552	CCCCCOC1[NH+]=C(N=C2N1c3ccccc3N2)N	-52.450	ZINC32922263	Cc1c(cnn1c2ccccc2)C(=O)n3c4ccccc4c(n3)N	-67.178
65	ZINC12433427	Cc1ccc(o1)c2cnnc(n2)NCc3cn4c(n3)SCC4	-52.410	ZINC12539590	CCCCn1c2c(c(=O)[nH]c1=O)n(c(n2)Cn3cnc(n3)C#N)CC	-67.169
66	ZINC09668025	c1ccc2c(c1)c(nc(n2)CSc3c(=O)[nH]c(=O)[nH]n3)O	-52.407	ZINC32507790	COc1ccc(c(c1)n2cccn2)c3cc(ccn3)C(=O)N	-67.157
67	ZINC23144715	c1ccc(cc1)c2[nH]cc(n2)C(=O)NCCc3cc(=O)[nH]cn3	-52.392	ZINC12580663	C=CCNc1nc(cs1)c2ccc3c(c2)[nH]c(=O)[nH]3	-67.115
68	ZINC30691145	c1ccc(c(c1)c2cc(no2)C(=O)Nc3nnc4n3cccc4)F	-52.368	ZINC11567913	c1cc2c(cc1c3ccc(=O)n(n3)CC(=O)[O-])CCCC2	-67.105
69	ZINC15974422	c1cnccc1CCNC(=O)CCC(=O)[O-]	-52.349	ZINC11658316	Cc1nc([nH]n1)SCC(=O)c2ccc3c(c2)-c4ccccc4C3	-67.052
70	ZINC05606472	c1cc2c(cc1S(=O)(=O)[O-])[nH]cc(c2=O)C(=O)[O-]	-52.326	ZINC20720131	CC(C(=O)Nc1ccccn1)NC(=O)C2COc3ccccc3O2	-67.052
71	ZINC23153533	c1ccc2c(c1)nc(o2)CNC(=O)Nc3[n-]c(nn3)C4CCCCC4	-52.298	ZINC19939112	c1ccc(cc1)c2ccccc2Cn3cnc4c3c(=O)nc([nH]c4=O)N	-67.008
72	ZINC05731338	c1ccc(cc1)C(=O)Cc2c(=O)nc-3[nH]onc3n2	-52.266	ZINC17027812	c1ccc2c(c1)OCC(O2)CCC(=O)N=c3[nH]ncs3	-66.984
73	ZINC06442642	CC(C(=O)[O-])NC(=O)c1cccc2c1Oc3ccccc3S2	-52.261	ZINC20862346	Cc1cnc(nc1c2cnn(c2)C)N3CCc4c(cnc(c4C#N)C)C3	-66.971
74	ZINC08512345	c1c(c(c(c2c1sc(=NC(=O)CSc3[nH]ncn3)[nH]2)F)F)F	-52.226	ZINC17940886	c1ccc2c(c1)c(c[nH]2)C(=O)OCc3nc(nc(n3)N)N	-66.941
75	ZINC32523728	c1cnc(nc1)NCc2c[nH]nc2c3ccc(cc3)F	-52.192	ZINC04932805	c1ccc2cc(ccc2c1)c3c(nco3)C(=O)Nc4cccnc4	-66.875
76	ZINC09205060	Cc1cc(no1)C(=O)Nc2nnc(o2)C3CCCCC3	-52.155	ZINC12979748	CN(Cc1nc2ccccc2s1)c3cn[nH]c(=O)c3Cl	-66.830
77	ZINC08577712	c1cc(c(cc1F)F)NC2=NC3(CCCCC3)[NH+]=C(N2)N	-52.129	ZINC28477561	Cc1c(c(=O)[nH]c(=O)[nH]1)CCC(=O)OCc2c(snn2)Cl	-66.784
78	ZINC20601465	c1ccnc(c1)c2nc(on2)CN3CCc4c(cn[nH]4)C3	-52.121	ZINC15879123	Cc1c(c(=O)[nH]c(=O)[nH]1)S(=O)(=O)N2CCCc3c2cccc3	-66.734
79	ZINC19974054	c1ccc(cc1)C=CC2CC(CC(=O)C2)Nc3c(non3)N	-52.086	ZINC09744013	Cc1c(c(n(n1)c2ccccc2)C)SCC(=O)Nc3nccs3	-66.682
80	ZINC19735184	Cc1cc(nc2c1c(=O)[nH]c(n2)SCC(=O)[O-])C	-52.068	ZINC14112009	CCn1cnnc1SC(C)C(=O)Nc2c3c([nH]cn3)ncn2	-66.674
81	ZINC33936291	Cc1c(c(n[nH]1)O)C(c2cn(c3c2cccc3)C)c4c([nH]nc4[O-])C	-52.055	ZINC14986539	CCC(=O)N1CCC(CC1)c2c(cn[nH]2)Cc3ccccc3	-66.665
82	ZINC23206528	Cc1cc(n(n1)CCC(=O)OCc2nc(nc(n2)N(C)C)N)C	-52.028	ZINC05753699	c1c(cnc(c1Cl)SCc2[nH]nc(n2)CC#N)C(F)(F)F	-66.631
83	ZINC04625773	Cn1ccnc1SCC(=O)Nc2cccc3c2c(=O)[nH][nH]c3=O	-51.927	ZINC22012815	C#CCC(Cc1cnc2c(n1)c(nc(n2)N)N)c3ccc(cc3)C(=O)[O-]	-66.551
84	ZINC30005705	CC(c1cc2ccccc2o1)N(C)Cc3nnnn3Cc4ccccc4	-51.903	ZINC32808506	CCOc1cc2c(cc1CNc3cn[nH]c(=O)c3Cl)OC(C2)C	-66.505
85	ZINC20211115	Cn1cc(nn1)NCc2c[nH]nc2c3cccc(c3)F	-51.844	ZINC19852920	CN1CCc2c(cc(c(n2)SCC(=O)N)C#N)C1	-66.460
86	ZINC20255187	Cc1cc(n(n1)C2CCN(C2)C(=O)Nc3nc([nH]n3)c4ccco4)C	-51.776	ZINC08746133	Cn1c(nnc1Sc2c3ccsc3ncn2)c4ccco4	-66.412
87	ZINC05730241	Cc1c2c(cc([nH+]1)C(=Nc3nccs3)[O-])c4ccccc4[nH]2	-51.737	ZINC11049269	c1ccc2c(c1)CCN2Cc3csc(n3)c4ccccn4	-66.351
88	ZINC04950695	c1ccc(cc1)c2cc(n(n2)C(CC(=O)[O-])C(=O)[O-])c3ccccc3	-51.734	ZINC20745937	c1ccc2c(c1)c(=O)n(cn2)CCNC(=O)c3ccco3	-66.298
89	ZINC10109357	c1ccc2c(c1)c(n[nH]c2=O)CC(=O)NCc3ccco3	-51.714	ZINC11738705	CC(=O)Nc1ccc(nn1)SCC(=O)Nc2nccs2	-66.281
90	ZINC16890080	C1CCC(CC1)CCCc2[nH]nnn2	-51.693	ZINC20539406	c1c(cn[nH]1)CCCC(=O)N2CCCC(C2)C(=O)N3CCOCC3	-66.248
91	ZINC07026624	c1ccc2c(c1)c(n[nH]2)c3nnc(o3)SCC(=O)NC4CC4	-51.669	ZINC18193970	c1ccc2c(c1)OCC(O2)c3csc4c3c(=O)[nH]cn4	-66.231
92	ZINC04777333	Cn1c2ccc(cc2n(c1=O)C)CNc3[n-]nnn3	-51.580	ZINC19148484	Cc1cc(on1)CN(C)C(=O)CCc2c(c(n[nH]2)C)C	-66.163
93	ZINC20601465	c1ccnc(c1)c2nc(on2)CN3CCc4c(cn[nH]4)C3	-51.564	ZINC31049595	c1ccc2c(c1)c(=O)n(nn2)CC(=O)OCc3ccccn3	-66.157
94	ZINC30795010	c1ccc2c(c1)c(=O)n(nn2)CC(=O)N3CCn4c3nc5c4cccc5	-51.547	ZINC16430737	c1cc(sc1)c2ccc3c4c(c(=O)[nH]nn4)sc3n2	-66.154
95	ZINC20601465	c1ccnc(c1)c2nc(on2)CN3CCc4c(cn[nH]4)C3	-51.539	ZINC06147346	c1ccc2c(c1)[nH]c(n2)C(C#N)C(=O)CSc3c(=O)[nH]c(=O)[nH]n3	-66.055
96	ZINC32592970	c1ccc2c(c1)c(=O)n(cn2)Cc3cc([nH]n3)C4CC4	-51.409	ZINC05757325	c1ccnc(c1)SCc2[nH]nc(n2)c3ccncc3	-66.051
97	ZINC31370784	Cc1c(c(=O)[nH]c(n1)C)CC(=O)NCC2Cc3ccccc3O2	-51.380	ZINC31555717	CSc1nncn1c2cccc(c2)NS(=O)(=O)c3ccccc3	-66.020
98	ZINC12199787	c1cc(oc1)CCNc2c(nc3c(n2)non3)NC4CC4	-51.366	ZINC15444522	CNS(=O)(=O)Cc1ccc2c(c1)c(c[nH]2)C(=O)OC	-66.000
99	ZINC32580864	CN1CCCc2c1ccc(c2)CNc3c4c([nH]cn4)nc(n3)N	-51.362	ZINC20455389	CN(Cc1cccc(c1)C(F)(F)F)C(=O)CCc2ccc(=O)[nH]n2	-65.986
100	ZINC29283930	Cc1c(nc2ccccc2n1)SCc3ccc(cc3)C(=O)N	-51.360	ZINC06420171	CN(C)S(=O)(=O)NCc1c2ccccc2c(=O)[nH]n1	-65.913

Figure S3. List of Top 100 Compounds Predicted to Bind in Each Pocket.

Dxr2-017 cancer cells 8 days

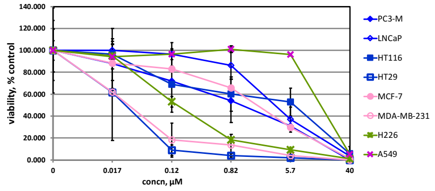

Dxr2-017 cancer cells 8 days Repeat

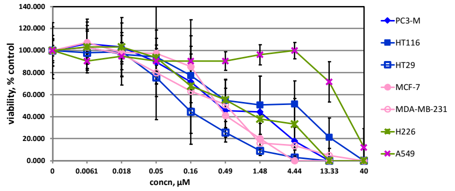

Dxr2-017 bone marrow, 8 and 14 days

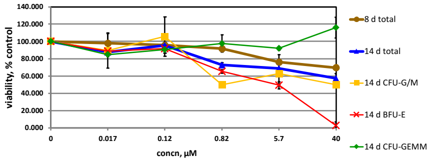

Dxr2-017 bone marrow, 8 and 14 days Repeat

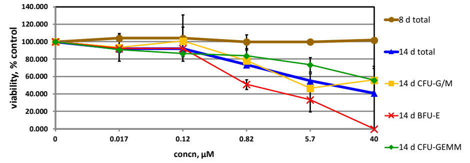

Dxr2-055 cancer cells 8 days

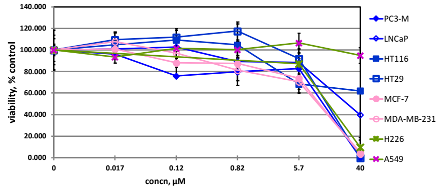

Dxr2-055 cancer cells 8 days Repeat

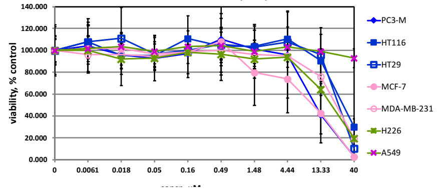

Dxr2-055 bone marrow, 8 and 14 days

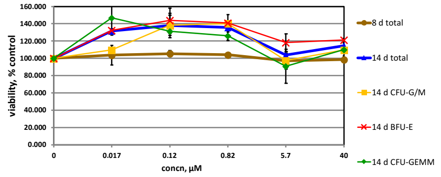

Dxr2-055 bone marrow, 8 and 14 days Repeat

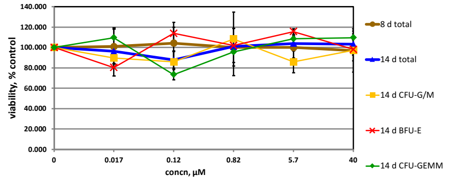

Dxr2-069 cancer cells 8 days

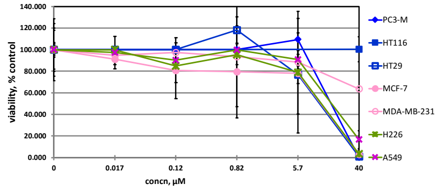

Dxr2-069 cancer cells 8 days Repeat

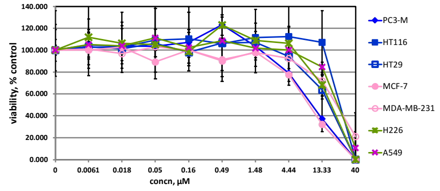

Dxr2-069 bone marrow, 8 and 14 days

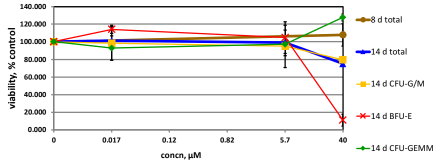

Dxr2-069 bone marrow, 8 and 14 days Repeat

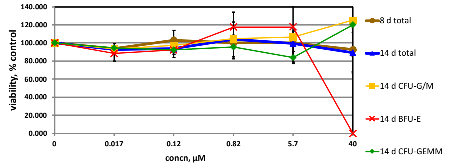

Dxr2-001 cncer cells 8 days

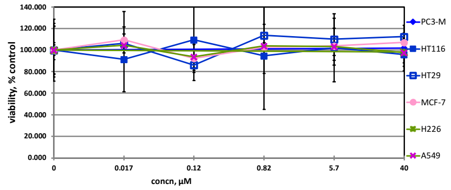

Dxr2-001 bone marrow, 8 and 14 days

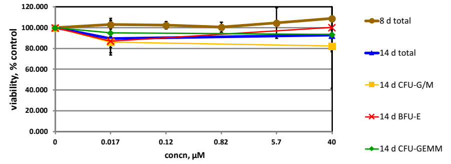

Dxr2-054 cancer cells 8 days

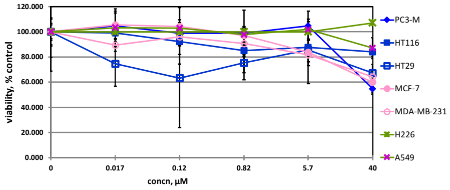

Dxr2-054 bone marrow, 8 and 14 days

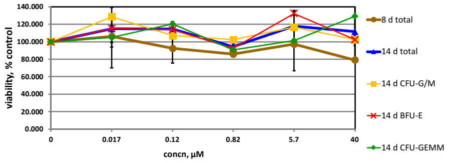

FolC2-001 cancer cells 8 days

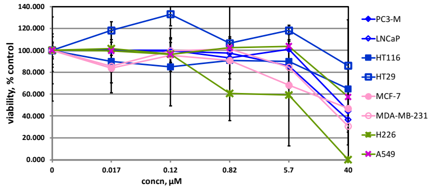

FolC2-001 cancer cells 8 days Repeat

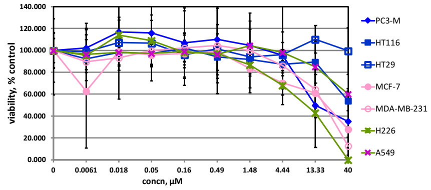

FolC2-001 bone marrow, 8 and 14 days

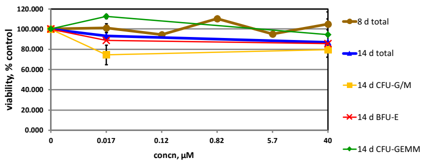

FolC2-001 bone marrow, 8 and 14 days Repeat

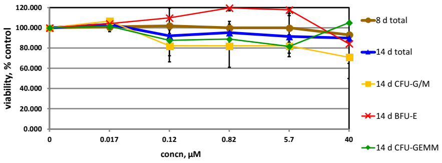

FolC2-005 cancer cells 8 days

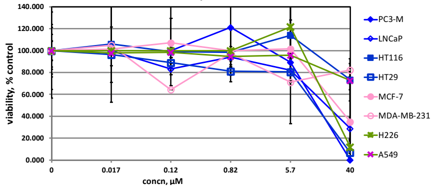

FolC2-001 cancer cells 8 days Repeat

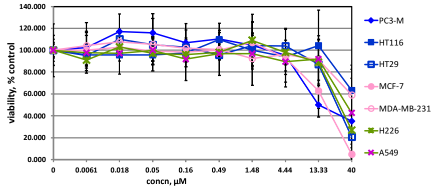

FolC2-005 bone marrow, 8 and 14 days

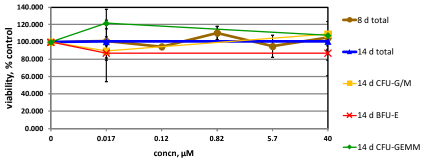

FolC2-005 bone marrow, 8 and 14 days Repeat

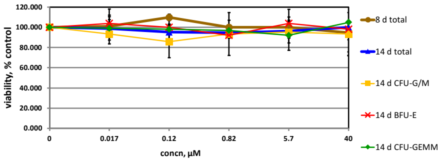

FolC2-044 cancer cells 8 days

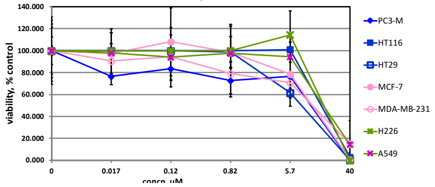

FolC2-044 cancer cells 8 days Repeat

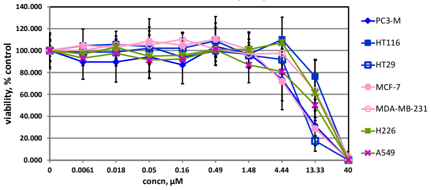

FolC2-044 bone marrow, 8 and 14 days

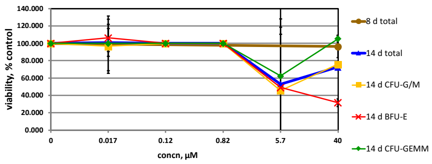

FolC2-044 bone marrow, 8 and 14 days Repeat

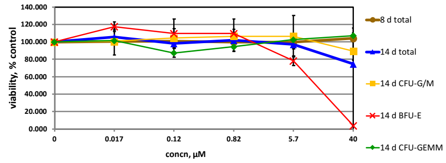

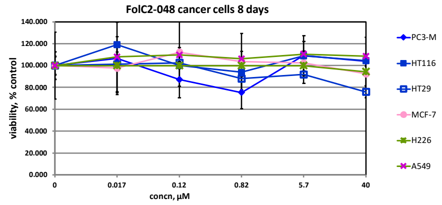
FolC2-048 cancer cells 8 days
viability, % control
concn, µM
PC3-M
HT116
HT29
MCF-7
H226
A549

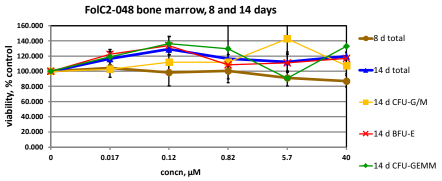
FolC2-048 bone marrow, 8 and 14 days
viability, % control
concn, µM
8 d total
14 d total
14 d CFU-G/M
14 d BFU-E
14 d CFU-GEMM

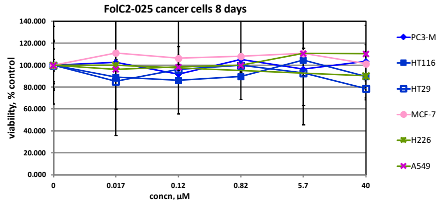
FolC2-025 cancer cells 8 days
viability, % control
concn, µM
PC3-M
HT116
HT29
MCF-7
H226
A549

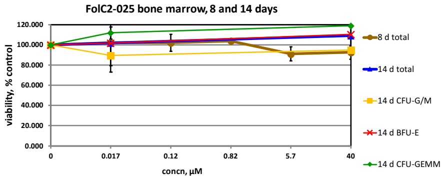
FolC2-025 bone marrow, 8 and 14 days
viability, % control
concn, µM
8 d total
14 d total
14 d CFU-G/M
14 d BFU-E
14 d CFU-GEMM

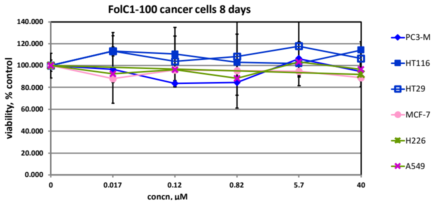
FolC1-100 cancer cells 8 days
viability, % control
concn, µM
PC3-M
HT116
HT29
MCF-7
H226
A549

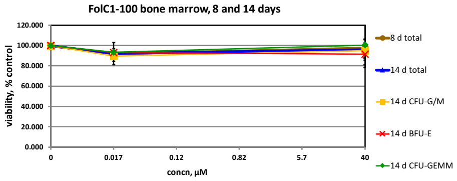
FolC1-100 bone marrow, 8 and 14 days
viability, % control
concn, µM
8 d total
14 d total
14 d CFU-G/M
14 d BFU-E
14 d CFU-GEMM

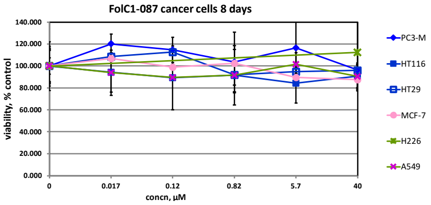
FolC1-087 cancer cells 8 days
viability, % control
concn, µM
PC3-M
HT116
HT29
MCF-7
H226
A549

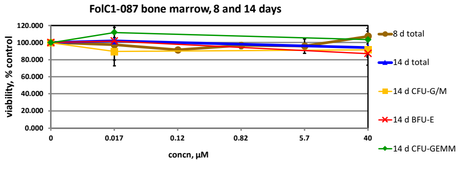
FolC1-087 bone marrow, 8 and 14 days
viability, % control
concn, µM
8 d total
14 d total
14 d CFU-G/M
14 d BFU-E
14 d CFU-GEMM

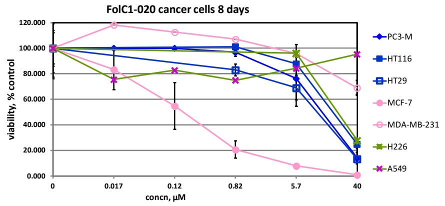
FolC1-020 cancer cells 8 days
viability, % control
concn, µM
PC3-M
HT116
HT29
MCF-7
MDA-MB-231
H226
A549

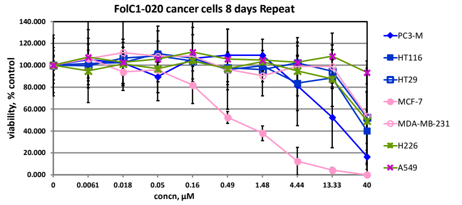
FolC1-020 cancer cells 8 days Repeat
viability, % control
concn, µM
PC3-M
HT116
HT29
MCF-7
MDA-MB-231
H226
A549

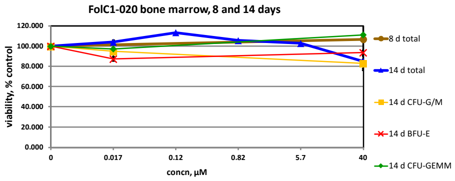
FolC1-020 bone marrow, 8 and 14 days
viability, % control
concn, µM
8 d total
14 d total
14 d CFU-G/M
14 d BFU-E
14 d CFU-GEMM

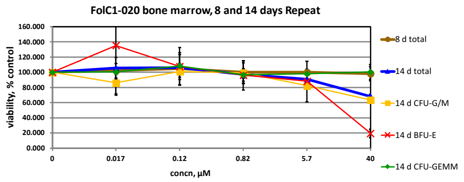
FolC1-020 bone marrow, 8 and 14 days Repeat
viability, % control
concn, µM
8 d total
14 d total
14 d CFU-G/M
14 d BFU-E
14 d CFU-GEMM

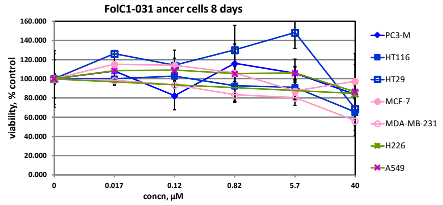
FolC1-031 ancer cells 8 days
viability, % control
concn, µM
PC3-M
HT116
HT29
MCF-7
MDA-MB-231
H226
A549

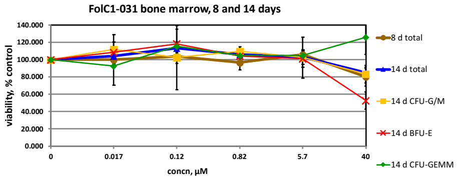
FolC1-031 bone marrow, 8 and 14 days
viability, % control
concn, µM
8 d total
14 d total
14 d CFU-G/M
14 d BFU-E
14 d CFU-GEMM

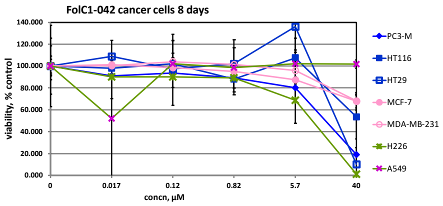
FolC1-042 cancer cells 8 days
viability, % control
concn, µM
PC3-M
HT116
HT29
MCF-7
MDA-MB-231
H226
A549

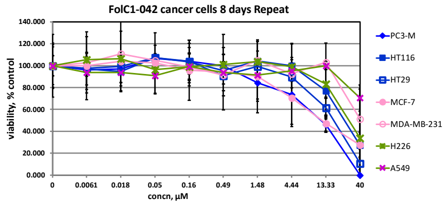
FolC1-042 cancer cells 8 days Repeat
viability, % control
concn, µM
PC3-M
HT116
HT29
MCF-7
MDA-MB-231
H226
A549

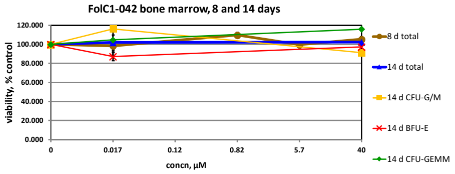
FolC1-042 bone marrow, 8 and 14 days
viability, % control
concn, µM
8 d total
14 d total
14 d CFU-G/M
14 d BFU-E
14 d CFU-GEMM

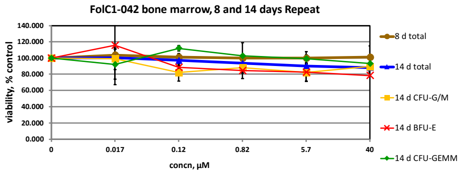
FolC1-042 bone marrow, 8 and 14 days Repeat
viability, % control
concn, µM
8 d total
14 d total
14 d CFU-G/M
14 d BFU-E
14 d CFU-GEMM

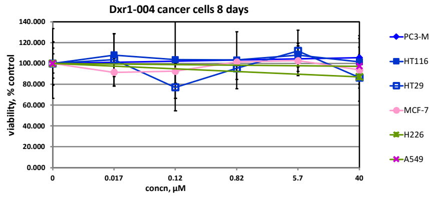
Dxr1-004 cancer cells 8 days
viability, % control
concn, µM
PC3-M
HT116
HT29
MCF-7
H226
A549

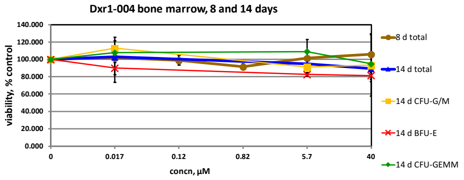
Dxr1-004 bone marrow, 8 and 14 days
viability, % control
concn, µM
8 d total
14 d total
14 d CFU-G/M
14 d BFU-E
14 d CFU-GEMM

Dxr1-026 cancer cells 8 days

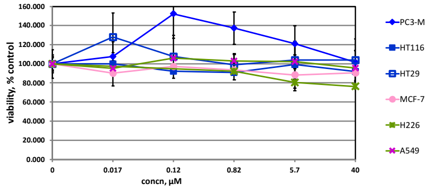

Dxr1-026 bone marrow, 8 and 14 days

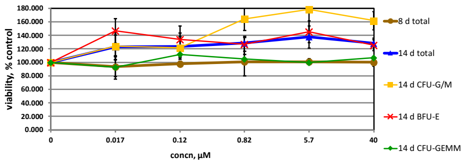

Dxr1-070 cancer cells 8 days

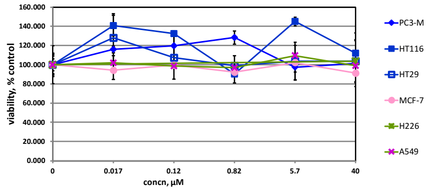

Dxr1-070 bone marrow, 8 and 14 days

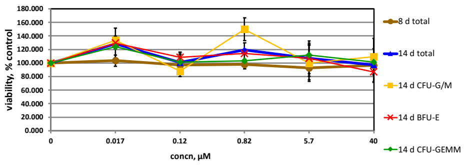

Dxr1-083 cancer cells 8 days

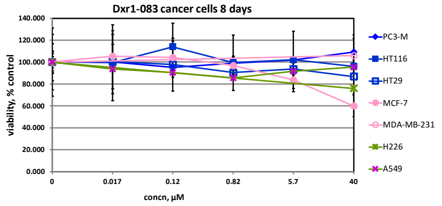

Dxr1-083 bone marrow, 8 and 14 days

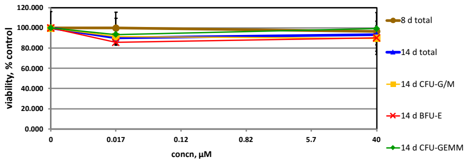

Dxr1-021 cancer cells 8 days

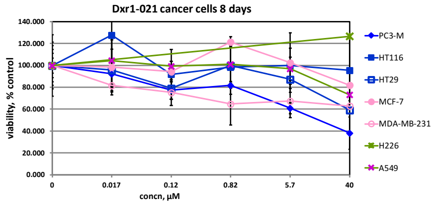

Dxr1-021 bone marrow, 8 and 14 days

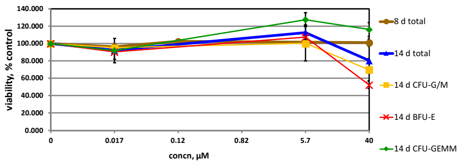

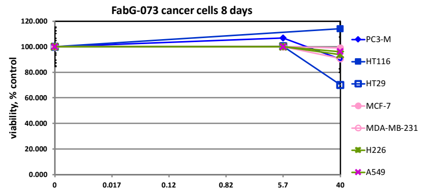
FabG-073 cancer cells 8 days
viability, % control
120.000
100.000
80.000
60.000
40.000
20.000
0.000
0
0.017
0.12
0.82
5.7
40
PC3-M
HT116
HT29
MCF-7
MDA-MB-231
H226
A549

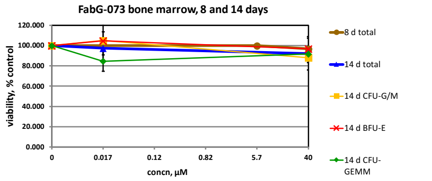
FabG-073 bone marrow, 8 and 14 days
viability, % control
120.000
100.000
80.000
60.000
40.000
20.000
0.000
0
0.017
0.12
0.82
5.7
40
concn, µM
8 d total
14 d total
14 d CFU-G/M
14 d BFU-E
14 d CFU-GEMM

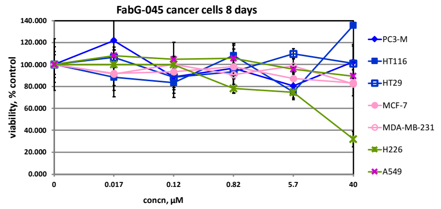
FabG-045 cancer cells 8 days
viability, % control
140.000
120.000
100.000
80.000
60.000
40.000
20.000
0.000
0
0.017
0.12
0.82
5.7
40
concn, µM
PC3-M
HT116
HT29
MCF-7
MDA-MB-231
H226
A549

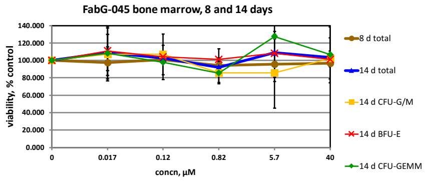
FabG-045 bone marrow, 8 and 14 days
viability, % control
140.000
120.000
100.000
80.000
60.000
40.000
20.000
0.000
0
0.017
0.12
0.82
5.7
40
concn, µM
8 d total
14 d total
14 d CFU-G/M
14 d BFU-E
14 d CFU-GEMM

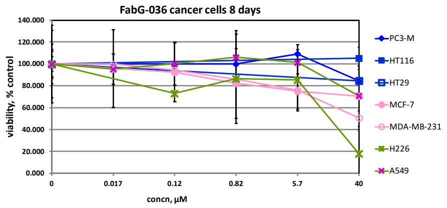
FabG-036 cancer cells 8 days
viability, % control
140.000
120.000
100.000
80.000
60.000
40.000
20.000
0.000
0
0.017
0.12
0.82
5.7
40
concn, µM
PC3-M
HT116
HT29
MCF-7
MDA-MB-231
H226
A549

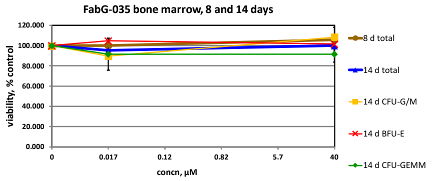
FabG-035 bone marrow, 8 and 14 days
viability, % control
120.000
100.000
80.000
60.000
40.000
20.000
0.000
0
0.017
0.12
0.82
5.7
40
concn, µM
8 d total
14 d total
14 d CFU-G/M
14 d BFU-E
14 d CFU-GEMM

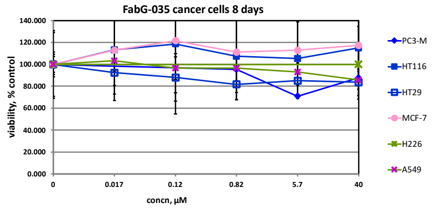
FabG-035 cancer cells 8 days
viability, % control
140.000
120.000
100.000
80.000
60.000
40.000
20.000
0.000
0
0.017
0.12
0.82
5.7
40
concn, µM
PC3-M
HT116
HT29
MCF-7
H226
A549

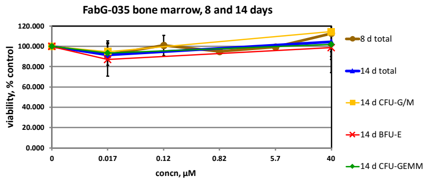
FabG-035 bone marrow, 8 and 14 days
viability, % control
120.000
100.000
80.000
60.000
40.000
20.000
0.000
0
0.017
0.12
0.82
5.7
40
concn, µM
8 d total
14 d total
14 d CFU-G/M
14 d BFU-E
14 d CFU-GEMM

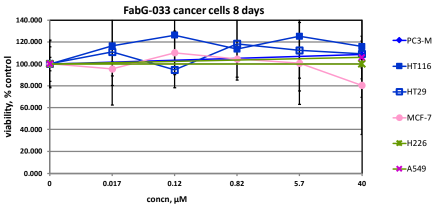
FabG-033 cancer cells 8 days
viability, % control
concn, µM
PC3-M
HT116
HT29
MCF-7
H226
A549

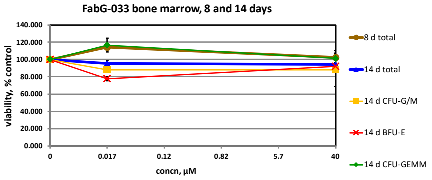
FabG-033 bone marrow, 8 and 14 days
viability, % control
concn, µM
8 d total
14 d total
14 d CFU-G/M
14 d BFU-E
14 d CFU-GEMM

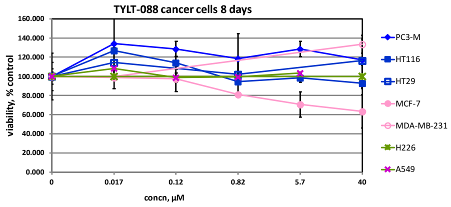
TYLT-088 cancer cells 8 days
viability, % control
concn, µM
PC3-M
HT116
HT29
MCF-7
MDA-MB-231
H226
A549

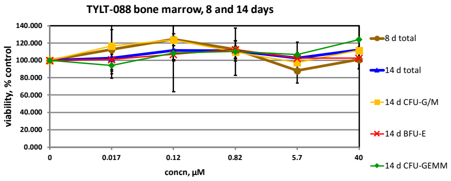
TYLT-088 bone marrow, 8 and 14 days
viability, % control
concn, µM
8 d total
14 d total
14 d CFU-G/M
14 d BFU-E
14 d CFU-GEMM

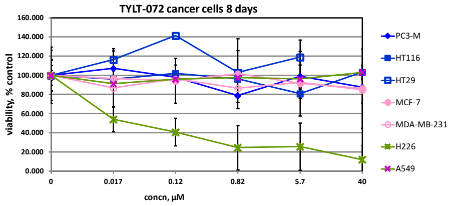
TYLT-072 cancer cells 8 days
viability, % control
concn, µM
PC3-M
HT116
HT29
MCF-7
MDA-MB-231
H226
A549

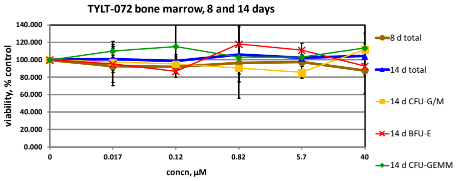
TYLT-072 bone marrow, 8 and 14 days
viability, % control
concn, µM
8 d total
14 d total
14 d CFU-G/M
14 d BFU-E
14 d CFU-GEMM

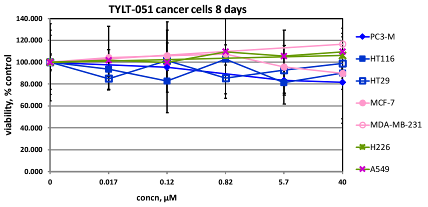
TYLT-051 cancer cells 8 days
viability, % control
concn, µM
PC3-M
HT116
HT29
MCF-7
MDA-MB-231
H226
A549

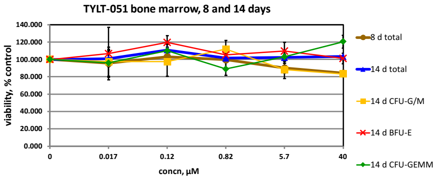
TYLT-051 bone marrow, 8 and 14 days
viability, % control
concn, µM
8 d total
14 d total
14 d CFU-G/M
14 d BFU-E
14 d CFU-GEMM

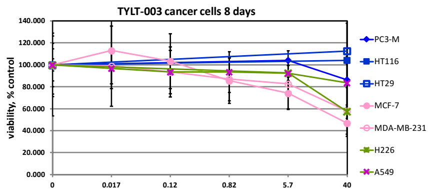
TYLT-003 cancer cells 8 days
viability, % control
PC3-M
HT116
HT29
MCF-7
MDA-MB-231
H226
A549

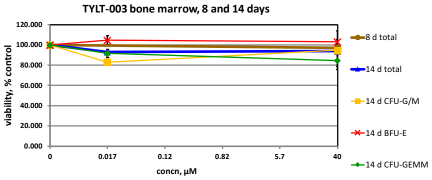
TYLT-003 bone marrow, 8 and 14 days
viability, % control
concn, µM
8 d total
14 d total
14 d CFU-G/M
14 d BFU-E
14 d CFU-GEMM

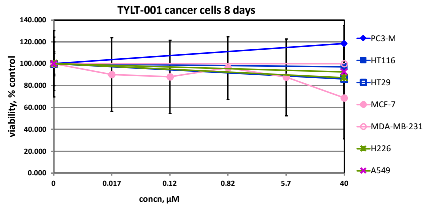
TYLT-001 cancer cells 8 days
viability, % control
concn, µM
PC3-M
HT116
HT29
MCF-7
MDA-MB-231
H226
A549

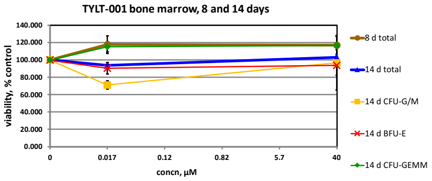
TYLT-001 bone marrow, 8 and 14 days
viability, % control
concn, µM
8 d total
14 d total
14 d CFU-G/M
14 d BFU-E
14 d CFU-GEMM

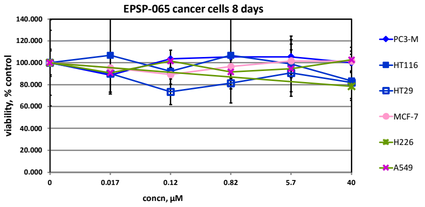
EPSP-065 cancer cells 8 days
viability, % control
concn, µM
PC3-M
HT116
HT29
MCF-7
H226
A549

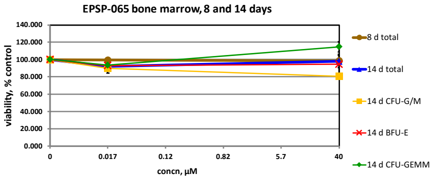
EPSP-065 bone marrow, 8 and 14 days
viability, % control
concn, µM
8 d total
14 d total
14 d CFU-G/M
14 d BFU-E
14 d CFU-GEMM

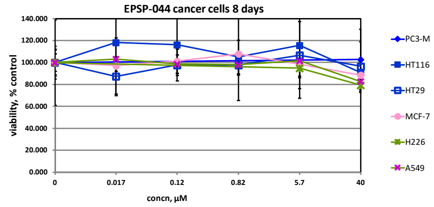
EPSP-044 cancer cells 8 days
viability, % control
concn, µM
PC3-M
HT116
HT29
MCF-7
H226
A549

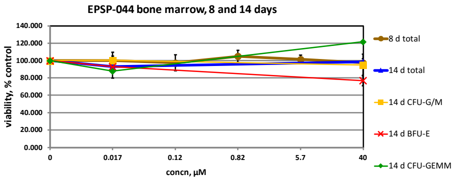
EPSP-044 bone marrow, 8 and 14 days
viability, % control
concn, µM
8 d total
14 d total
14 d CFU-G/M
14 d BFU-E
14 d CFU-GEMM

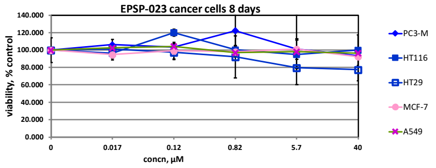
EPSP-023 cancer cells 8 days
viability, % control
concn, µM
PC3-M
HT116
HT29
MCF-7
A549

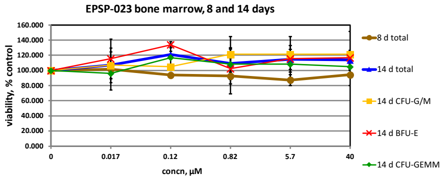
EPSP-023 bone marrow, 8 and 14 days
viability, % control
concn, µM
8 d total
14 d total
14 d CFU-G/M
14 d BFU-E
14 d CFU-GEMM

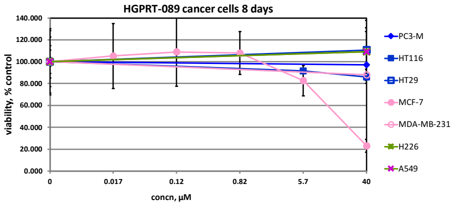
HGPRT-089 cancer cells 8 days
viability, % control
concn, µM
PC3-M
HT116
HT29
MCF-7
MDA-MB-231
H226
A549

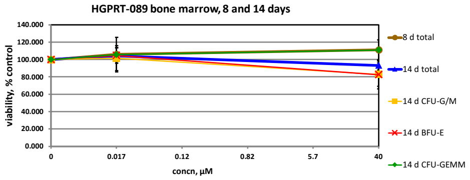
HGPRT-089 bone marrow, 8 and 14 days
viability, % control
concn, µM
8 d total
14 d total
14 d CFU-G/M
14 d BFU-E
14 d CFU-GEMM

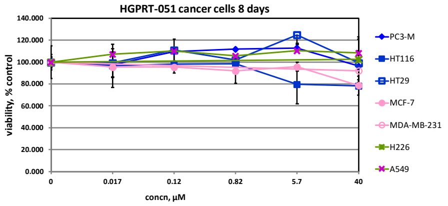
HGPRT-051 cancer cells 8 days
viability, % control
concn, µM
PC3-M
HT116
HT29
MCF-7
MDA-MB-231
H226
A549

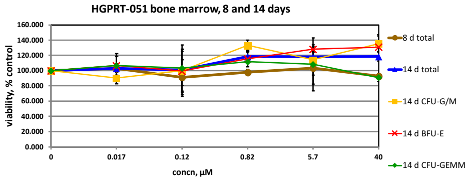
HGPRT-051 bone marrow, 8 and 14 days
viability, % control
concn, µM
8 d total
14 d total
14 d CFU-G/M
14 d BFU-E
14 d CFU-GEMM

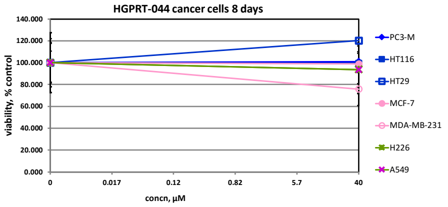
HGPRT-044 cancer cells 8 days
viability, % control
concn, µM
PC3-M
HT116
HT29
MCF-7
MDA-MB-231
H226
A549

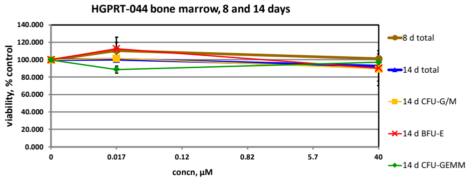
HGPRT-044 bone marrow, 8 and 14 days
viability, % control
concn, µM
8 d total
14 d total
14 d CFU-G/M
14 d BFU-E
14 d CFU-GEMM

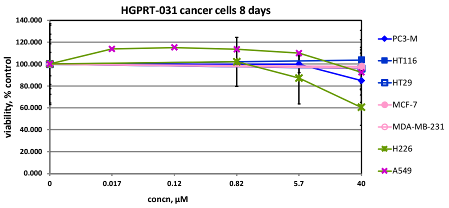
HGPRT-031 cancer cells 8 days
viability, % control
concn, µM
PC3-M
HT116
HT29
MCF-7
MDA-MB-231
H226
A549

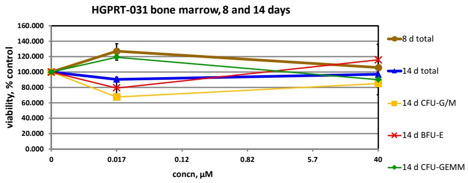
HGPRT-031 bone marrow, 8 and 14 days
viability, % control
concn, µM
8 d total
14 d total
14 d CFU-G/M
14 d BFU-E
14 d CFU-GEMM

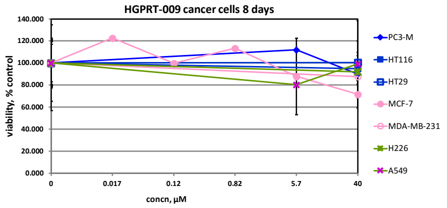
HGPRT-009 cancer cells 8 days
viability, % control
concn, µM
PC3-M
HT116
HT29
MCF-7
MDA-MB-231
H226
A549

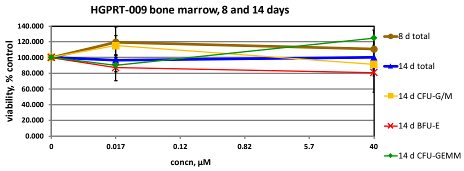
HGPRT-009 bone marrow, 8 and 14 days
viability, % control
concn, µM
8 d total
14 d total
14 d CFU-G/M
14 d BFU-E
14 d CFU-GEMM

Figure S4. Effect of Compounds on Cancer Cell Growth and Hematopoietic Stem Cell Toxicity. For human cancer cell lines, eight-day coloney formation assays were conducted. For CD34+ human cord blood hematopoietic stem cells, at eight days the total number of colonies were counted. At fourteen days, the number of colonies in the following hematopoietic lineages were counted: granulocytic (white blood cells), erythrocytic (red blood cells) and megakaryocytic (platelets), measured by the formation of CFU-GM, BFU-E and CFU-GEMM colonies, respectively. All assays were run in replicates of N=2, data expressed as the mean ± SD percentage of control. Initial findings meeting stipulated criteria were repeated, also in replicates of N=2. For initial screening, cells were treated with 0 (vehicle), 0.017, 0.12, 0.82, 5.7 and 40 µM of the denoted compound. For replicate experiments, cancer cells were treated with 0 (vehicle), 0.0061, 0.018, 0.05, 0.16, 0.49, 1.48, 4.4, 13.3 and 40 µM compound. For cancer cells, compound was added one day after plating. For hematopoietic assays, compound was introduced into semisolid media on the day of plating.

Supplementary Table 4. Efficacy ratios at 40 μM compound for all compounds*

	Dxr2 site					FolC2 site					FolC1 site					Dxr1 site				
**cell line**	**Dxr2-017**	**Dxr2-055**	**Dxr2-069**	**Dxr2-001**	**Dxr2-054**	**FolC2-001**	**FolC2-005**	**FolC2-025**	**FolC2-044**	**FolC2-048**	**FolC1-042**	**FolC1-031**	**FolC1-020**	**FolC1-087**	**FolC1-100**	**Dxr1-021**	**Dxr1-083**	**Dxr1-070**	**Dxr1-026**	**Dxr1-005**
HT116	0.08	0.63	0.93	0.88	1.06	0.60	0.70	0.97	0.03	1.20	0.51	0.82	0.23	0.84	1.17	0.95	1.00	1.16	0.92	0.96
HT29	0.00	0.00	0.01	1.03	0.85	0.79	0.06	0.85	0.00	0.87	0.10	0.86	0.12	0.89	1.09	0.59	0.90	1.08	1.04	0.81
MCF-7	0.02	0.04	0.03	0.98	0.76	0.43	0.33	1.09	0.01	1.06	0.64	1.22	0.01	0.81	0.91	0.81	0.62	0.95	0.91	0.89
MDAMB231	0.00	0.05	0.59	-	0.81	0.28	0.78	-	0.16	-	0.65	0.70	0.65	-	-	0.62	1.10	-	-	-
H226	0.02	0.10	0.03	0.90	1.35	0.00	0.11	0.98	0.00	1.09	0.01	1.06	0.26	1.05	0.94	1.26	0.79	1.08	0.76	0.82
A549	0.07	0.96	0.15	0.90	1.10	0.53	0.69	1.19	0.15	1.25	0.96	1.08	0.89	0.84	0.99	0.72	0.99	1.03	0.95	0.92
PC3-M	0.00	0.00	0.00	0.94	0.69	0.41	0.00	1.12	0.00	1.19	0.18	1.04	0.13	0.90	0.97	0.38	1.13	1.05	1.01	1.00
LNCaP	0.05	0.40	-	-	-	0.34	0.28	-	-	-	-	-	-	-	-	-	-	-	-	-
**combined data**																				
relative activity for a given compound, %¥	100	75	71	0	0	63	63	0	100	0	43	0	73	0	0	14	0	0	0	0
relative activity for a given site	3 of 5 (60%) compounds active					3 of 4 (75%) compounds active					2 of 5 (40%) compounds active					1 of 5 (20%) compounds active				

	PRT site					EPSP site			TYLT site					FabG site				
**cell line**	**PRT-009**	**PRT-031**	**PRT-044**	**PRT-051**	**PRT-P89**	**EPSP-023**	**EPSP-044**	**EPSP-065**	**TYLT-001**	**TYLT-003**	**TYLT-051**	**TYLT-072**	**TYLT-088**	**FabG-033**	**FabG-035**	**FabG-036**	**FabG-045**	**FabG-073**
HT116	0.86	0.98	0.98	0.84	0.77	1.06	0.93	0.85	0.83	1.07	1.07	1.18	0.92	1.13	1.02	0.99	1.40	1.18
HT29	0.91	0.91	1.19	1.07	1.00	0.82	0.99	0.83	0.74	1.16	1.17	2.05	1.15	1.07	0.74	0.80	1.04	0.72
MCF-7	0.65	0.93	0.98	0.85	0.21	0.98	0.91	1.03	0.59	0.48	1.07	0.98	0.63	0.79	1.04	0.67	0.86	1.02
MDAMB231	0.79	0.91	0.75	0.99	0.79	-	-	-	0.85	0.60	1.38	0.97	1.32	-	-	0.48	0.85	0.94
H226	0.83	0.57	0.93	1.11	0.57	-	0.81	0.80	0.75	0.59	1.26	0.14	0.99	0.98	0.89	0.17	0.33	0.97
A549	0.89	0.87	0.92	1.17	0.98	1.03	0.85	1.04	0.79	0.86	1.30	1.17	0.98	1.04	0.76	0.67	0.92	0.99
PC3-M	0.81	0.80	1.00	1.04	0.87	0.99	1.05	1.02	1.01	0.89	0.97	0.99	1.16	1.06	0.78	0.80	1.06	0.93
LNCaP	-	-	-	-	-	-	-	-	-	-	-	-	-	-	-	-	-	-
**combined data**																		
relative activity for a given compound, %¥	0	0	0	0	0	0	0	0	0	0	0	14	0	0	0	0	0	0
relative activity for a given site	0% active					0% active			1 of 5 (20%) compounds active					0% active				

*efficacy ratio = % viable cancer cell line colonies/% viable bone marrow colonies, at 8 days; all assays were run in replicates of N=2 and resultant means used for calculations; those with an efficacy ratio of below 0.5 are denoted in blue font

¥those with efficacy ratio <0.5 were considered active

Figure S5. The Efficacy Ratio of All Compounds and All Cancer Cell Lines Tested. Cancer cells and human bone marrow stem cells were treated with the indicated compound at 40 μM, and the resultant efficacy ratio is shown. The efficacy ratio = (percent of remaining cancer cell colonies)/(percent of remaining bone marrow colonies), at 8 days after treatment.

Developmental Therapeutics Program
One Dose Mean Graph

NSC: D-767026 / 1 | **Conc:** 1.00E-5 Molar | **Experiment ID:** 1209OS39

Dxr2-017

Panel/Cell Line	Growth Percent
**Leukemia**	
CCRF-CEM	55.96
HL-60(TB)	64.59
K-562	55.78
MOLT-4	52.98
RPMI-8226	58.72
SR	42.13
**Non-Small Cell Lung Cancer**	
A549/ATCC	35.50
HOP-62	77.10
HOP-92	76.97
NCI-H226	70.39
NCI-H23	82.27
NCI-H322M	69.31
NCI-H460	68.48
NCI-H522	59.72
**Colon Cancer**	
COLO 205	59.53
HCC-2998	46.47
HCT-116	73.42
HCT-15	59.01
HT29	74.58
KM12	49.67
SW-620	37.70
**CNS Cancer**	
SF-268	66.08
SF-539	75.29
SNB-19	75.83
SNB-75	91.72
U251	81.37
**Melanoma**	
LOX IMVI	3.23
MALME-3M	72.83
M14	38.33
MDA-MB-435	67.49
SK-MEL-2	84.52
SK-MEL-28	98.63
SK-MEL-5	29.07
UACC-257	-2.02
UACC-62	24.28
**Ovarian Cancer**	
IGROV1	72.94
OVCAR-3	81.89
OVCAR-4	112.36
OVCAR-5	62.52
OVCAR-8	59.57
NCI/ADR-RES	62.60
SK-OV-3	94.69
**Renal Cancer**	
786-0	85.25
A498	94.76
ACHN	38.64
CAKI-1	50.51
RXF 393	76.34
SN12C	69.56
TK-10	93.33
UO-31	67.72
**Prostate Cancer**	
PC-3	55.92
DU-145	56.39
**Breast Cancer**	
MCF7	58.41
MDA-MB-231/ATCC	17.19
BT-549	76.02
T-47D	58.89
MDA-MB-468	56.62
Mean	62.79
Delta	64.81
Range	114.38

Mean Growth Percent - Growth Percent

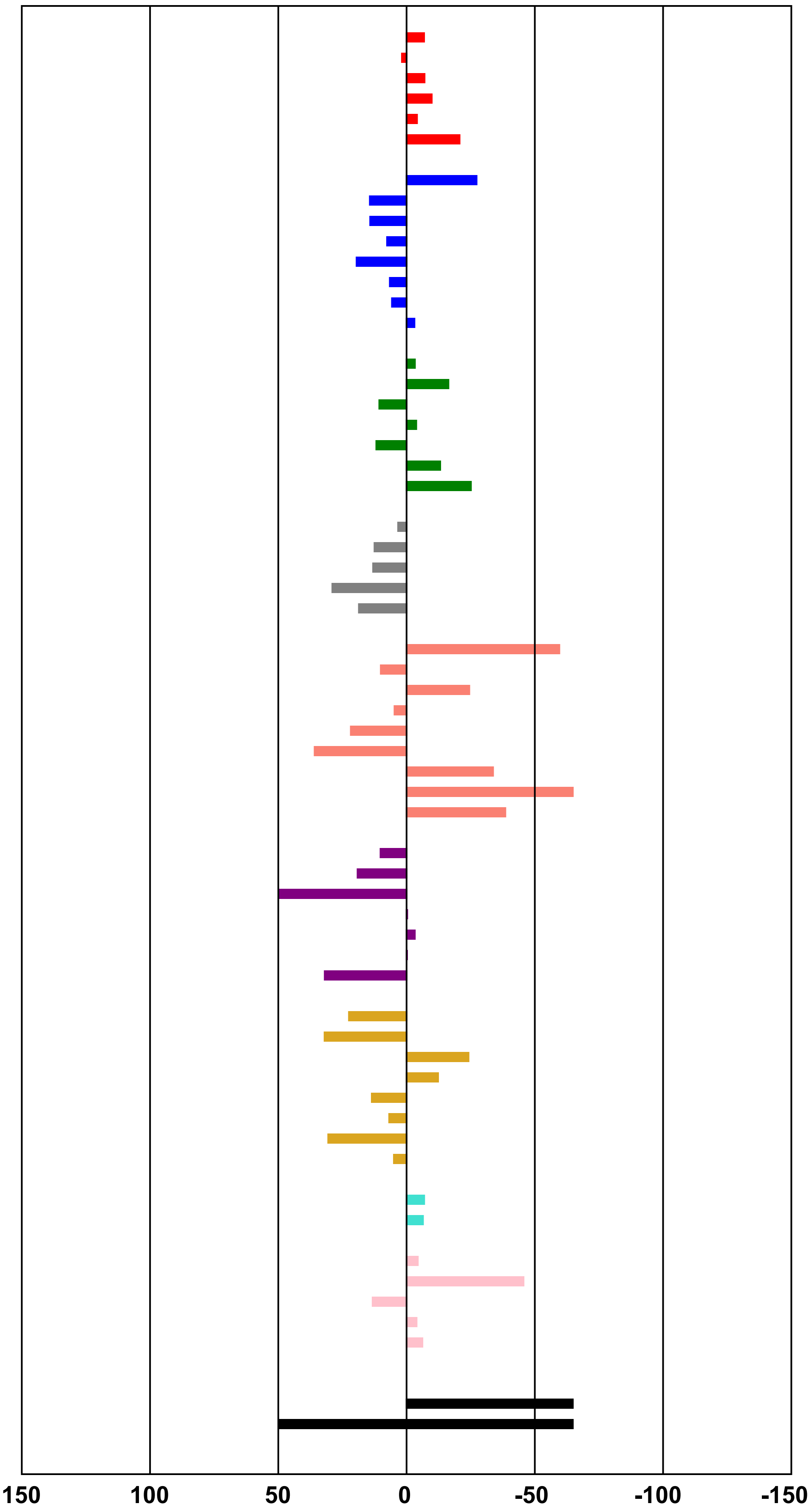

Developmental Therapeutics Program | **NSC:** D-767024 / 1 | **Conc:** 1.00E-5 Molar | FolC2-001

One Dose Mean Graph | **Experiment ID:** 1209OS39

Panel/Cell Line	Growth Percent
Leukemia	
CCRF-CEM	90.38
HL-60(TB)	100.70
K-562	104.21
MOLT-4	89.82
RPMI-8226	100.11
SR	94.33
Non-Small Cell Lung Cancer	
A549/ATCC	98.45
HOP-62	76.99
HOP-92	85.23
NCI-H226	82.12
NCI-H23	99.99
NCI-H322M	93.52
NCI-H460	99.36
NCI-H522	96.73
Colon Cancer	
COLO 205	93.33
HCC-2998	104.09
HCT-116	96.31
HCT-15	95.13
HT29	104.96
KM12	109.20
SW-620	97.57
CNS Cancer	
SF-268	99.42
SF-539	87.96
SNB-19	101.96
SNB-75	59.62
U251	104.29
Melanoma	
LOX IMVI	96.96
MALME-3M	84.51
M14	102.36
MDA-MB-435	102.52
SK-MEL-2	102.21
SK-MEL-28	95.90
SK-MEL-5	88.82
UACC-257	91.34
UACC-62	95.95
Ovarian Cancer	
IGROV1	74.24
OVCAR-3	95.24
OVCAR-4	98.71
OVCAR-5	89.76
OVCAR-8	81.30
NCI/ADR-RES	93.51
SK-OV-3	87.15
Renal Cancer	
786-0	104.32
A498	116.13
ACHN	87.06
CAKI-1	84.56
RXF 393	97.02
SN12C	102.53
TK-10	117.03
UO-31	72.50
Prostate Cancer	
PC-3	83.45
DU-145	112.53
Breast Cancer	
MCF7	87.80
MDA-MB-231/ATCC	88.22
HS 578T	88.56
BT-549	89.37
T-47D	103.25
MDA-MB-468	105.73
Mean	94.59
Delta	34.97
Range	57.41

Mean Growth Percent - Growth Percent

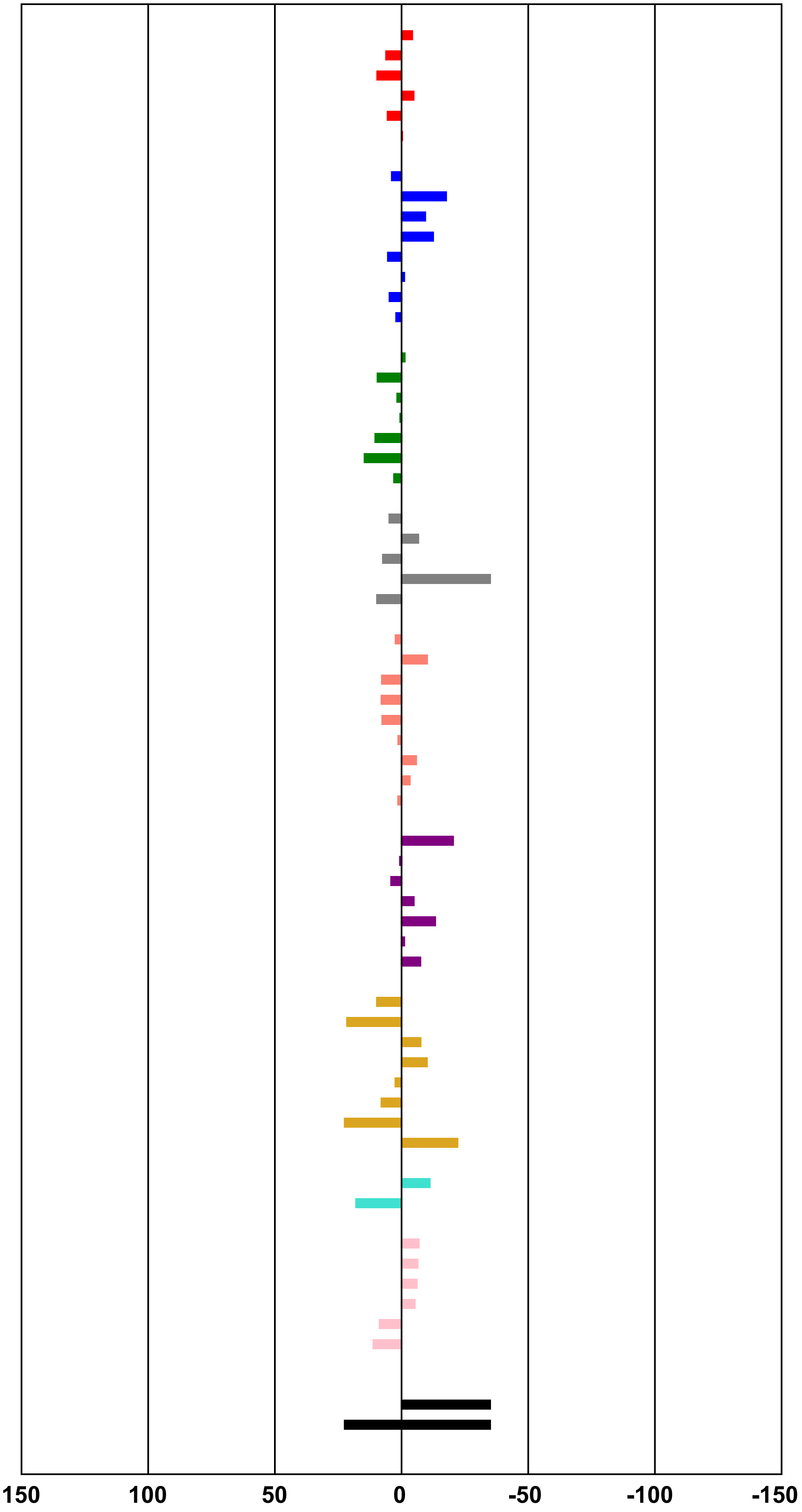

Figure S6. NCI-60 cell line screens. COMPARE plots for Dxr2-017 and FolC2-001 are shown.

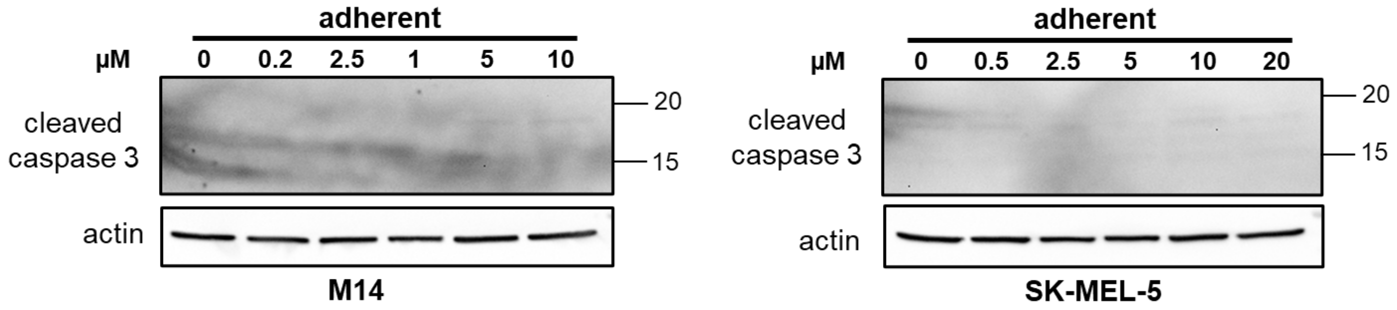

Figure S7. Dxr2-017 did not induce apoptosis in adherent cells. M14 or SK-MEL-5 cells were plated onto 6 well plates, and 24 hours later they were treated with the denoted concentrations of Dxr2-017. After 3 days, cells were washed with PBS, the remaining adherent cells were lysed and resultant lysate was probed by Western blot for cleaved caspase 3. The depicted cleaved caspase 3 Western blots were exposed for a long period of time, as denoted by the evident high background signal.

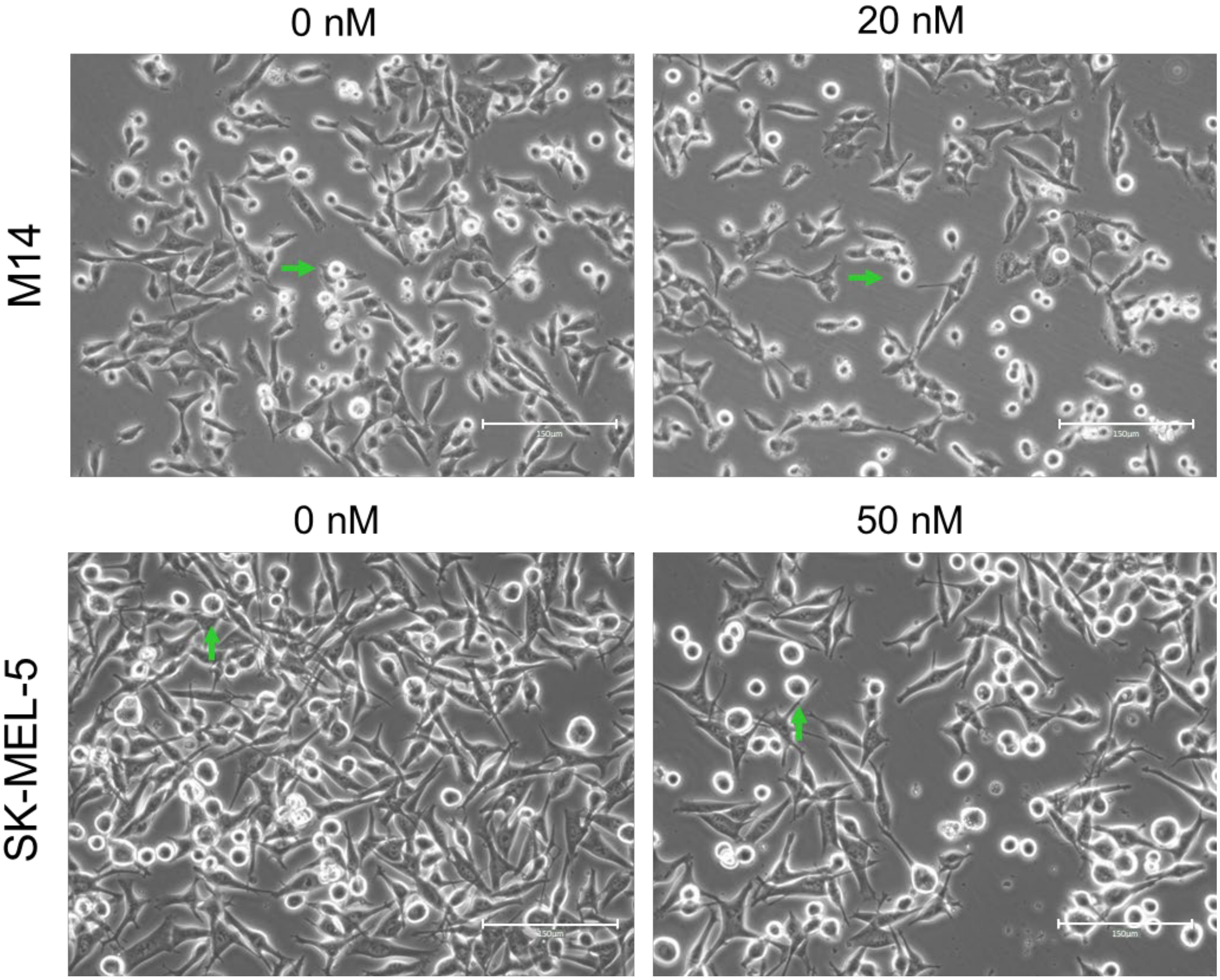

Figure S8. Dxr2-017 increases floating cells and does not alter adherent cell morphology. Cells were treated for eight days with Dxr2-017 at concentrations close to the IC50 value for each cell line examined. Depicted are representative light photomicrographs at 20X; scale bar is150 µm. Green arrows denote floating cells.
